# Synthesis of Phosphatidyl Glycerol Containing Unsymmetric Acyl Chains Using H-Phosphonate Methodology

**DOI:** 10.3390/molecules27072199

**Published:** 2022-03-28

**Authors:** Zachary J. Struzik, Shruti Biyani, Tim Grotzer, Judith Storch, David H. Thompson

**Affiliations:** 1 Bindley Bioscience Center, Department of Chemistry, Multi-Disciplinary Cancer Research Facility, Purdue University, 1203 W. State Street, West Lafayette, IN 47907, USA; zstruzik@purdue.edu (Z.J.S.); biyanis@purdue.edu (S.B.); tgrotzer@purdue.edu (T.G.); 2 Department of Nutritional Sciences and Rutgers Center for Lipid Research, Rutgers University, New Brunswick, NJ 08901, USA; storch@sebs.rutgers.edu

**Keywords:** phospholipid synthesis, phosphatidyl glycerol, H-phosphonates, phosphoramidites

## Abstract

Naturally occurring phospholipids, such as phosphatidyl glycerol (PG), are gaining interest due to the roles they play in disease mechanisms. To elucidate the metabolism of PG, an optically pure material is required, but this is unfortunately not commercially available. Our previous PG synthesis route utilized phosphoramidite methodology that addressed issues surrounding fatty acid substrate scope and glycerol backbone modifications prior to headgroup phosphorylation, but faltered in the reproducibility of the overall pathway due to purification challenges. Herein, we present a robust pathway to optically pure PG in fewer steps, utilizing H-phosphonates that features a chromatographically friendly and stable triethyl ammonium H-phosphonate salt. Our route is also amendable to the simultaneous installation of different acyl chains, either saturated or unsaturated, on the glycerol backbone.

## 1. Introduction

**Figure 1:**
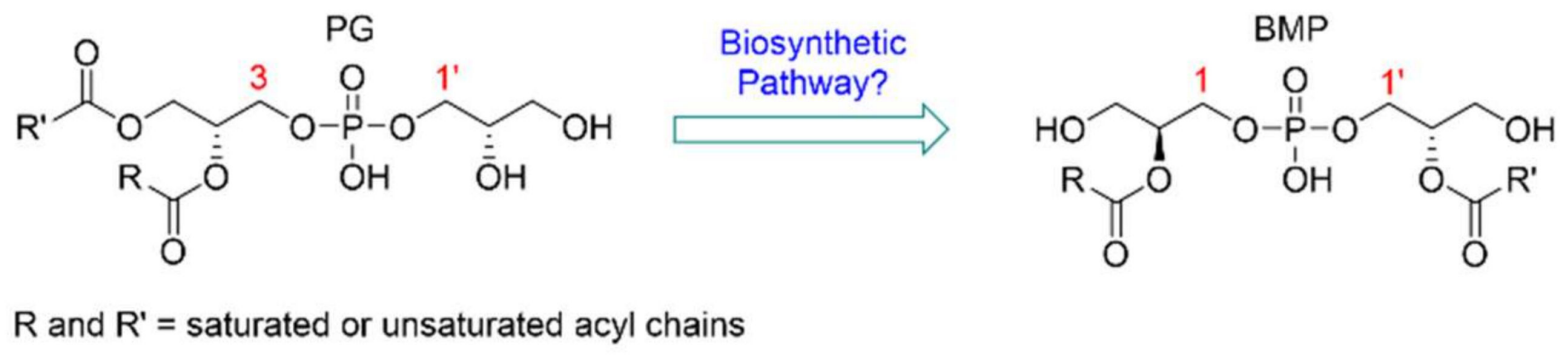
PG as a hypothesized precursor to BMP.

Naturally occurring phospholipids (PLs) are biological molecules that are a major component of cell membranes. PLs contain a polar headgroup and glycerol backbone bearing two fatty acyl chains, usually located on the *sn*-1 and *sn*-2 positions of *sn*-3-phosphoglycerol. The most common PL headgroups are choline, ethanolamine, serine, inositol, and glycerol [1]. Bismonoacylglycerophosphate (BMP), also known as lysobisphosphatidic acid (LBPA), is an isomer of phosphatidylglycerol (PG) that comprises less than 1% of the total cellular membrane PL, but about 15% of the lysosomal PL, suggesting that it is a lysosome-specific phospholipid [2,3]. BMP is an unusual PL in that the phosphoglycerol backbone is phosphorylated at the *sn*-1 position instead of the usual *sn*-3 position (Figure 1) [4,5]. Several studies have suggested that PG is the biosynthetic precursor to BMP [6,7,8]; however, the underlying metabolic pathway(s) are unknown, nor has the catabolic metabolism of PG been reported. As an initial step to elucidate the conversion of PG to BMP, we sought the preparation of optically pure PG.

**Figure 2:**
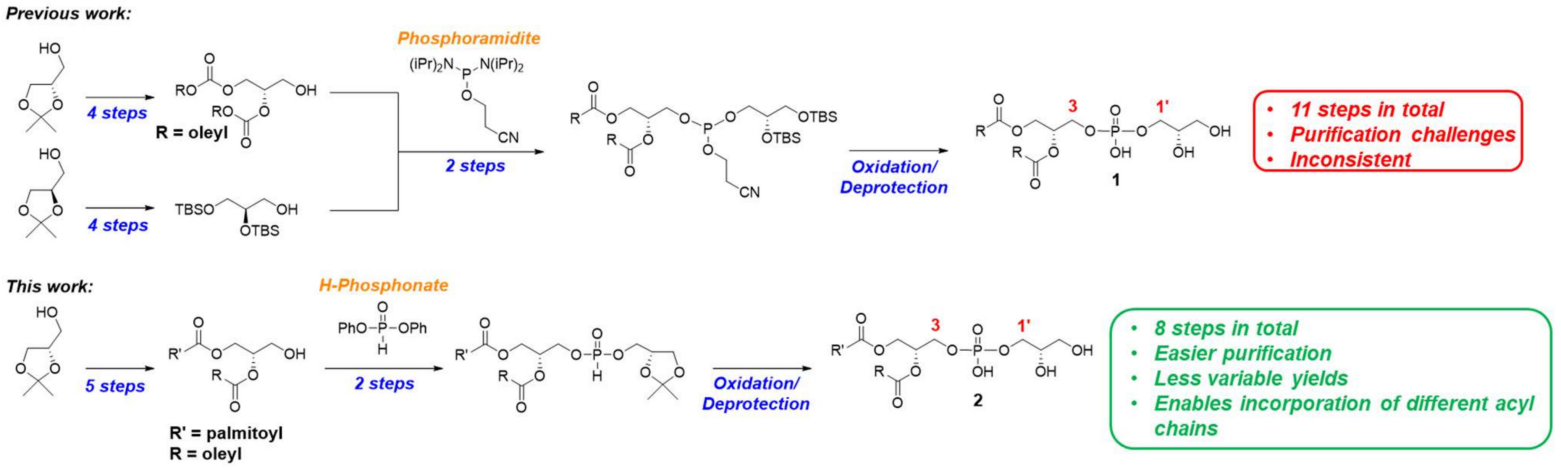
PG was previously synthesized using phosphoramidite methodology (**top**). A more efficient route was developed by incorporating H-phosphonates as the phosphonylating agent (**bottom**).

This challenge requires an efficient route to allow PG-bearing unsymmetric acyl chains to enable the chemical dissection of the BMP biosynthetic pathway. Unfortunately, optically pure derivatives of unsymmetric PG are not commercially available. We previously reported on the synthesis of diastereochemically pure PG [9] using phosphoramidite precursors that are commonly utilized in oligonucleotide synthesis (Figure 2) [10]. This strategy addressed issues regarding the previous syntheses of PG [11,12,13], such as the ability to incorporate unsaturated acyl chains without concerns regarding olefin reduction and allowing for the early modification of glycerol headgroup alcohols prior to generation of the phosphoglycerol diester. While **1** was synthesized in modest yield, the use of an aliphatic ammonium fluoride source such as TBAF in the final global deprotection step to simultaneously remove the bulky silyl ethers of the phosphoglycerol headgroup and the cyanoethyl-protecting group of the phosphate resulted in batch-to-batch inconsistencies and suboptimal yields. Our experience has shown that this can be attributed to the necessity of rigorous chromatographic conditions for final-product isolation; specifically, a highly polar (aqueous) mobile phase on silica flash columns [14], an ion exchange column [15,16], and a prep HPLC separation [17]. Additionally, this routing does not allow for the installation of different acyl chains. Given these significant limitations, a more efficient and consistent path was sought to enable the manipulation of any segment of the PG structure. To avoid the inconsistency and chromatographic burden of the previous synthesis, we report a novel route to PG that takes advantage of H-phosphonate methodology using diphenyl phosphite [18,19,20,21,22,23,24] as the phosphorylating agent. This approach features a bench- and air-stable H-phosphonate salt intermediate developed during the synthesis of phosphatidyl serine [20] that simplifies subsequent phosphorylation reactions and purification conditions. Additionally, we were able to reduce the number of synthetic steps from eleven to eight by directly installing an acetonide-protected glycerol headgroup without further modification of the primary and secondary alcohols, an effort that was previously needed to retain the desired stereochemistry of the PG product.

## 2. Results and Discussion

Initial efforts to improve the synthetic route involved substituting fluoride sources [25,26,27] in the deprotection step and substituting diphenylmethylsilyl ethers (DPMS) [28] on the phosphoglycerol headgroup instead of TBS ethers. Through a series of high-throughput experiments using desorption electrospray ionization mass spectrometry [29,30], and the modification of solketal protection from p-methoxybenzyl ether (PMB) [31,32] to a phenyl acyl ester, followed by translation to flow chemistry [33], we were able to successfully phosphorylate the protected phosphoglycerol head group on the gram scale, allowing us to upscale the synthesis of phosphoramidite intermediate in higher yields compared to the batch methods. Unfortunately, subsequent acyl chain migration and deprotection of the labile DPMS groups prevented us from moving forward with the phosphoramidite approach. For a more complete discussion of these efforts, please see the Supplementary Information. Despite multiple attempts to perform the desired transformation using phosphoramidites, we abandoned this approach and began to explore other phosphonylation strategies to achieve a robust and reproducible method of PG synthesis.

H-phosphonates are a class of phosphonylated intermediates that have been used in the syntheses of other glycerophospholipids, including phosphatidyl inositols [34,35,36,37], phosphatidyl ethanolamine [38], phosphatidyl choline [38], and phosphatidyl serine [20,38]. They have also been used in the total synthesis of glycophospholipids [39,40,41], as well as in nucleoside-based phospholipids [24] and drugs [42]. To our surprise, they have not, however, been utilized in the synthesis of PG. The attractiveness of this methodology was based on the ability to obtain a phosphonylated intermediate in the form of an organic salt that can be readily purified by chromatography on polar stationary phases such as silica or alumina. Another advantage of this strategy is that the phosphite precursor to H-phosphonates can undergo transesterifications reactions under basic conditions with alcohols, an ideal circumstance for installation of the phosphorous species on the glycerol backbone to prevent acyl chain migration from the sn-2 to the sn-1 position. A third advantage is that H-phosphonate monoesters such as **9** are less susceptible to air oxidation, as well as base- and acid-catalyzed hydrolysis, due to the high level of electron density associated with the anionic form of the phosphonate, whereas the phosphonate proton needs to be removed before undergoing a nucleophilic attack [18,43,44,45,46,47]. Once activated, H-phosphonates have exhibited high rates of condensation, with alcohols approaching 10^5^ M s^−1^ [47,48], further supporting the case for their use. In most of the previous examples, the H-phosphonate intermediates were synthesized by reacting the alcohol substrate with PCl_3_ and imidazole, followed by the introduction of the second glycerol derivative with a coupling agent such as pivaloyl chloride [38]. Due to the air and moisture sensitivity of PCl_3_, we employed a strategy by Mallik et al., utilizing a low-cost diphenyl phosphite as the phosphonylation reagent for quantitative conversion of substrate [20]. Additionally, the use of the phenolate leaving group further enhanced transesterification [47].

**Scheme 1:**
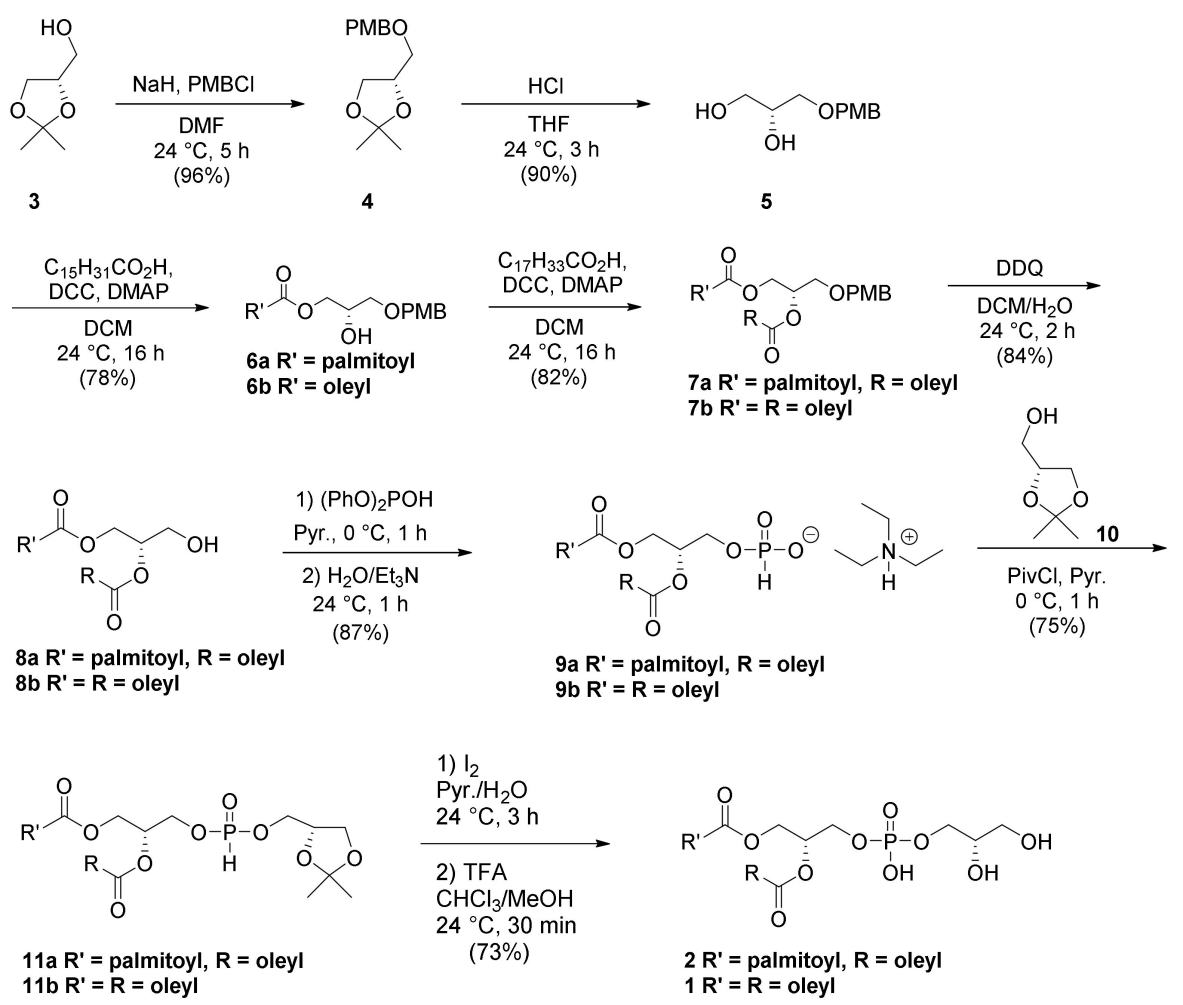
Synthesis of PG containing different acyl chains using H-phosphonates.

In our previous synthesis of PG [9], we designed the phosphoglycerol headgroup such that the final deprotection step avoided the use of functional groups that required deprotection under acidic conditions to obviate potential acyl chain migration side reactions. Additionally, it was convenient to simultaneously remove the cyanoethyl group and silyl groups under mildly basic fluoride conditions to avoid multiple purification steps after formation of the phosphate. Thus, we replaced the acetonide of solketal with silyl groups that could be removed in the presence of TBAF. Encouraged by the robustness of H-phosphonates in our hands, we were curious as to how much of a concern the use of harsh acidic conditions would be when discovering an efficient PG route. To explore this question, we decided to phosphonylate solketal precursor **10** directly after the installation of the backbone (Scheme 1). If the acetonide could be removed in the presence of acid without acyl chain migration or other major obstacles that would negatively impact overall yield, this would streamline the synthesis by four steps relative to the phosphoramidite route [9]. We also wanted to determine the scope of conditions that would enable the successful installation of asymmetric acyl chains on the *sn*-1 and *sn*-2 positions of the glycerol backbone. We were pleased to observe chemoselective control of each **5** alcohol esterification reaction simply by limiting the fatty acid stoichiometry in the reaction. The formation of the diesterified product comprised only about 5% of the yield. While the esterification of the primary hydroxyl is kinetically favorable, we were unsure how much reaction at the secondary hydroxyl group would affect the product distribution. Thus, we were able to successfully synthesize **6** and **7** using standard Steglich esterification conditions in 78% and 82% yields, respectively. Deprotection of the PMB ether with DDQ afforded **8** in 84% yield without substantial acyl chain migration of either the symmetrical or asymmetrical acyl chain versions of the target. We were able to successfully phosphorylate **8** with diphenyl phosphite (6 equivalents) in pyridine at 0 °C, and subsequent quenching in aqueous conditions, to obtain an easy-to-handle phosphonate salt **9** in 87% yield. The acetonide protected head-group was esterified with **10** using pivaloyl chloride in pyridine at 0 °C to afford **11** in 75% yield. While protocols using this chemistry generally call for 3–6 equivalents of the coupling agent, we found that using the lower end of that range (~3 equivalents) resulted in fewer by-products, a higher yield, and simplified purification, since homocoupled pyrophosphates generated by condensation of H-phosphonates and pivalic acid after transesterification with an alcohol were found to be problematic at higher equivalencies. Finally, oxidation of the H-phosphonate from P(III) to P(V) was conducted in the usual manner with I_2_ in a pyridine/water mixture. Once the solvent was removed in vacuo, the crude product was placed directly in a 5:1:0.5 CHCl_3_:TFA:MeOH mixture to remove the acetonide of **11** to provide PG **1** and **2** in 73% yield. While this reaction can be performed in this manner without a purification step between the oxidation and acetonide deprotection, we recommend a chromatographic purification between each step. The product was consistently cleaner by ^1^H and ^31^P NMR analysis, and the yield of **1** and **2** did not vary significantly, when incorporating an additional purification step.

We have demonstrated the synthesis of diastereochemically pure PG containing both symmetric and asymmetric acyl chains using H-phosphonate methodology. Due to the simpler purification and handling of the phosphonylated intermediates via the use of diphenyl phosphite, this approach gave PG in higher isolated yields with more consistent results than the phosphoramidite approach. Moving forward, we hope to be able to apply this chemistry to the investigation of PG metabolism.

## 3. Materials and Methods

### 3.1. General Information

Commercial reagents were used as purchased from TCI Chemicals (Portland, OR, USA) and MilliporeSigma (Burlington, MA, USA). Organic solvents used were reagent grade, purchased from Fisher Scientific (Hampton, NH, USA). Dry solvents were purified using a Glass Contour Solvent System from Pure Process Technology, LLC (Nashua, NH, USA), with Fisher HPLC grade DCM, Aldrich anhydrous DMF, and Fisher HPLC/ACS grade THF. Reactions were monitored by thin-layer chromatography using silica gel 60 F254 plates (Merck, Darmstadt, Germany). UV light (254 nm) and staining with aqueous KMnO_4_ was used to visualize the developed chromatograms. Flash chromatography was performed using a Biotage SP4 A2A0 with RediSep Rf silica flash columns (12 g, 60 mg–1.2 g sample size) and collected in 9 mL fraction volumes or performed via manual silica column chromatography using silica gel 60 (MilliporeSigma, Burlington, MA, USA). Compounds purified via automatic flash chromatography are accompanied by a gradient table in the Supplementary Material. All ^1^H, ^13^C and ^31^P NMR spectra were recorded on a Bruker-AV-III-500-HD instrument. Chemical shifts (δ) are reported in parts per million, relative to CDCl_3_ (^1^H NMR residual peak at δ = 7.26 ppm, ^13^C NMR residual peak at δ = 77.0 ppm), and coupling constants (*J*) are given in Hz. High-resolution mass measurements were acquired on an Agilent 6550 iFunnel LC/Q-TOF mass spectrometer (Agilent Technologies, Santa Clara, CA, USA).

### 3.2. Synthesis of (S)-4-(((4-methoxybenzyl)oxy)methyl)-2,2-dimethyl-1,3-dioxolane (4)

NaH (2.71 g, 113 mmol) was added to an oven-dried, 250-mL, multi-neck, round-bottom flask. Schlenk techniques were utilized to evacuate the flask, and then dry DMF (50 mL) was added. The flask was maintained under a continuous Ar atmosphere for the duration of the reaction. The reaction flask was lowered into an ice bath and (*S*)-solketal (5.0 g, 38 mmol) was added dropwise and stirred vigorously at 0 °C for 45 min. 4-Methoxybenzyl chloride (6.6 g, 42 mmol) was added and stirred at 20 °C for 4 h. The completed reaction was slowly quenched with saturated NH_4_Cl solution and extracted with ethyl acetate (3 × 150 mL). Combined organic extracts were washed with water (2 × 150 mL), washed with brine (2 × 150 mL), dried over anhydrous Na_2_SO_4_, and concentrated under reduced pressure. The crude product was purified on a 5 cm silica gel column (100:0 to 98:2 DCM:MeOH) to yield the desired product as a yellow oil in 99% yield; [α]_D_^27^ + 205 (*c* 0.049, CHCl_3_); R_f_ = 0.50 (DCM:MeOH); ^1^H NMR (500 MHz, CDCl_3_) δ 7.28–7.23 (m, 2H), 6.91–6.84 (m, 2H), 4.50 (q, *J* = 11.7 Hz, 2H), 4.28 (p, *J* = 6.0 Hz, 1H), 4.04 (dd, *J* = 8.3, 6.4 Hz, 1H), 3.80 (s, 3H), 3.72 (dd, *J* = 8.3, 6.3 Hz, 1H), 3.52 (dd, *J* = 9.8, 5.7 Hz, 1H), 3.44 (dd, *J* = 9.8, 5.6 Hz, 1H), 1.42 (s, 3H), 1.36 (s, 3H); ^13^C NMR (126 MHz, CDCl_3_) δ 159.3, 130.1, 129.4, 113.8, 109.4, 77.3, 77.1, 76.8, 74.8, 73.2, 70.8, 77.0, 55.3, 26.8, 25.4. QTOF-HRMS (ESI) for C_14_H_20_O_4_ [M+Na^+^]: found 275.1255, calcd 275.1254.

### 3.3. Synthesis of (R)-3-(benzyloxy)propane-1,2-diol (5)

Aqueous 1M HCl (21 mL) was added to a 100-mL, round-bottom flask containing a solution of **4** (1.46 g, 8 mmol) in 21 mL THF. The reaction mixture was allowed to react at 20 °C for 2 h. Saturated NaHCO_3_ solution was added to quench the reaction and the reaction mixture was extracted with EtOAc (3 × 100 mL). The combined organic extracts were dried with anhydrous Na_2_SO_4_ and concentrated under reduced pressure. The crude product was purified on a Biotage flash purification system (DCM:MeOH) to yield the product as a white solid in 93% yield (refer to Table S8 for gradient); [α]_D_^27^-12.1 (*c* 0.025, CHCl_3_); R_f_ = 0.13 (DCM:MeOH 98:2); ^1^H NMR (500 MHz, CDCl_3_) δ 7.25–7.20 (m, 2H), 6.89–6.83 (m, 2H), 4.45 (s, 2H), 3.84 (tt, *J* = 6.1, 4.0 Hz, 1H), 3.78 (s, 3H), 3.63 (dd, *J* = 11.5, 3.7 Hz, 1H), 3.55 (dd, *J* = 11.5, 5.9 Hz, 1H), 3.51–3.42 (m, 2H), 3.10 (s, 2H). ^13^C NMR (126 MHz, CDCl_3_) δ 159.4, 129.8, 129.7, 129.6, 129.5, 114.0, 113.9, 77.4, 77.1, 76.9, 73.2, 71.4, 70.8, 64.1, 55.3. QTOF-HRMS (ESI) for C_11_H_16_O_4_ [M+Na^+^]: found 235.0943., calcd 235.0941.

### 3.4. Synthesis of (S)-2-hydroxy-3-((4-methoxybenzyl)oxy)propyl palmitate (6a)

Palmitic acid (1.09 g, 4.8 mmol) and **5a** (1.06 g, 5 mmol) were placed in an oven-dried, 100-mL, three-neck, round-bottom flask. The contents of the flask were cycled three times with vacuum/Ar, and the flask was equipped with an Ar balloon and dry DCM (15 mL) was added. A solution of DCC (0.97 g, 4.7 mmol) and DMAP (0.58 g, 4.7 mmol) in dry DCM (15 mL) was prepared in a separate 100-mL, round-bottom flask and equipped with an Ar balloon. This solution was transferred to the three-neck reaction flask via a cannula and stirred at 20 °C for 16 h. The salt was removed by vacuum filtration through a coarse glass frit containing Celite, and the resulting filtrate was collected and concentrated under reduced pressure. A minimal amount of hexane was added to the crude product, and the mixture was sonicated to dissolve the solid. The resulting solution was purified on a Biotage flash purification system (hexane:EtOAc) to yield the desired product as a clear white solid with 78% yield (refer to Table S9 for gradient); [α]_D_^25^ +19.1 (*c* 0.038, CHCl_3_) R_f_ = 0.25 (Hexane:EtOAc, 8:2); ^1^H NMR (500 MHz, CDCl_3_) δ 7.27–7.22 (m, 2H), 6.91–6.84 (m, 2H), 4.48 (s, 2H), 4.20–4.07 (m, 2H), 4.01 (tt, *J* = 6.1, 4.4 Hz, 1H), 3.80 (s, 3H), 3.55–3.41 (m, 2H), 2.31 (t, *J* = 7.6 Hz, 2H), 1.60 (p, *J* = 7.3 Hz, 2H), 1.25 (s, 24H), 0.88 (t, *J* = 6.9 Hz, 3H); ^13^C NMR (126 MHz, CDCl_3_) δ 174.0, 159.4, 129.8, 129.4, 113.9, 77.3, 77.3, 77.1, 76.8, 73.2, 73.0, 70.6, 68.9, 68.7, 65.4, 55.3, 34.2, 31.9, 29.7, 29.7, 29.6, 29.5, 29.4, 29.3, 29.2, 24.9, 22.7, 14.1. QTOF-HRMS (ESI) for C_27_H_46_O_5_ [M+Na^+^]: found 473.3235, calcd 473.3237.

*(S)-2-Hydroxy-3-((4-methoxybenzyl)oxy)propyl oleate (6b)**.*** Clear oil in 76% yield; [α]_D_^25^ +27 (*c* 0.011, CHCl_3_) R_f_ = 0.178 (Hexane:EtOAc, 8:2); ^1^H NMR (500 MHz, CDCl_3_) δ 7.26–7.22 (m, 2H), 6.91–6.85 (m, 2H), 5.39–5.30 (m, 2H), 4.48 (s, 2H), 4.20–4.08 (m, 2H), 4.01 (tt, *J* = 6.2, 4.4 Hz, 1H), 3.80 (s, 3H), 3.55–3.42 (m, 2H), 2.31 (t, *J* = 7.6 Hz, 2H), 2.04–1.97 (m, 4H), 1.61 (p, *J* = 7.3 Hz, 2H), 1.38–1.17 (m, 26H), 0.88 (t, *J* = 6.9 Hz, 3H); ^13^C NMR (126 MHz, CDCl_3_) δ 173.9, 159.4, 130.0, 129.8, 129.4, 113.9, 77.3, 77.1, 76.8, 73.2, 70.6, 68.9, 65.4, 55.3, 34.2, 31.9, 29.8, 29.7, 29.5, 29.4, 29.3, 29.2, 29.1, 27.2, 27.2, 27.1, 24.9, 22.7, 14.14. QTOF-HRMS (ESI) for C_29_H_48_O_5_ [M+Na^+^]: found 499.3391, calcd 499.3394.

### 3.5. Synthesis of (S)-3-((4-methoxybenzyl)oxy)propane-1,2-diyl dioleate (7b)

Oleic acid (2.82 g, 10 mmol) and **6b** (2.38 g, 5 mmol) were placed in an oven-dried, 100-mL, three-neck, round-bottom flask. The contents of the flask were treated with three cycles of vacuum/Ar, the flask was equipped with an Ar balloon, and dry DCM (15 mL) was added. A solution of DCC (2.3 g, 11 mmol) and DMAP (1.3 g, 11 mmol) in dry DCM (15 mL) was prepared in a separate, 100-mL, round-bottom flask equipped with an Ar balloon. This solution was transferred to the three-neck reaction flask via a cannula and stirred at 20 °C for 16 h. The formed salt was removed by vacuum filtration through a coarse glass frit containing Celite, and the resulting filtrate was collected and concentrated under reduced pressure. A minimal amount of hexane was added to the crude product, and the mixture was sonicated to dissolve the solid. The resulting solution was purified on a 4-cm-diameter, 32-cm-long manual silica gel column (hexane:EtOAc) to yield the desired product as a clear, colorless oil in 82% yield. The use of a shorter column resulted in the co-elution of oleic acid. A Biotage method was also developed for this method (Hex:EtOAc) (refer to Table S10 for gradient); [α]_D_^25^ +51 (*c* 0.020, CHCl_3_); R_f_ = 0.63 (Hexane:EtOAc, 8:2); ^1^H NMR (500 MHz, CDCl_3_) δ 7.25–7.20 (m, 2H), 6.89–6.84 (m, 2H), 5.39–5.29 (m, 4H), 5.22 (dtd, *J* = 6.5, 5.2, 3.7 Hz, 1H), 4.53–4.41 (m, 2H), 4.33 (dd, *J* = 11.8, 3.8 Hz, 1H), 4.17 (dd, *J* = 11.9, 6.4 Hz, 1H), 3.80 (s, 3H), 3.55 (dd, *J* = 5.2, 1.9 Hz, 2H), 2.29 (dt, *J* = 20.5, 7.5 Hz, 4H), 2.05–1.96 (m, 8H), 1.66–1.57 (m, 4H), 1.38–1.21 (m, 42H), 0.88 (t, *J* = 6.9 Hz, 6H). ^13^C NMR (126 MHz, CDCl_3_) δ 173.4, 173.1, 159.3, 130.0, 129.8, 129.7, 129.3, 113.8, 77.3, 77.0, 76.8, 73.0, 70.0, 67.9, 62.7, 55.3, 34.3, 34.1, 31.9, 29.8, 29.7, 29.5, 29.3, 29.2, 29.2, 29.1, 29.1, 27.2, 27.2, 25.0, 24.9, 22.7, 14.1. QTOF-HRMS (ESI) for C_47_H_80_O_6_ [M+H^+^]: found 741.6027, calcd 741.6027.

*(S)-1-((4-Methoxybenzyl)oxy)-3-(palmitoyloxy)propan-2-yl oleate (7a).* Clear oil in 80% yield. [α]_D_^24^ +57.8 (*c* 0.017, CHCl_3_); R_f_ = 0.68 (Hexane:EtOAc, 8:2); ^1^H NMR (500 MHz, CDCl_3_) δ 7.25–7.21 (m, 2H), 6.90–6.85 (m, 2H), 5.38–5.30 (m, 2H), 5.25–5.19 (m, 1H), 4.47 (q, *J* = 11.7 Hz, 2H), 4.33 (dd, *J* = 11.9, 3.8 Hz, 1H), 4.17 (dd, *J* = 11.9, 6.4 Hz, 1H), 3.80 (s, 3H), 3.55 (dd, *J* = 5.2, 1.9 Hz, 2H), 2.29 (dt, *J* = 20.6, 7.5 Hz, 4H), 2.06–1.96 (m, 4H), 1.60 (dp, *J* = 14.4, 7.3 Hz, 4H), 1.37–1.19 (m, 47H), 0.88 (t, *J* = 6.9 Hz, 6H). ^13^C NMR (126 MHz, CDCl_3_) δ 173.4, 173.1, 159.3, 130.0, 129.8, 129.7, 129.3, 113.8, 77.3, 77.0, 76.8, 73.0, 70.0, 67.9, 62.7, 55.3, 34.3, 34.1, 32.0, 29.8, 29.7, 29.7, 29.7, 29.6, 29.5, 29.4, 29.3, 29.3, 29.2, 29.2, 29.1, 27.2, 27.2, 25.0, 24.9, 22.7, 14.1; QTOF-HRMS (ESI) for C_45_H_78_O_6_ [M+H^+^]: found 715.5872, calcd 715.5871.

### 3.6. Synthesis of (S)-3-Hydroxypropane-1,2-diyl dioleate (8b)

DCM/H_2_O (15 mL, 5% H_2_O) and **7b** (1.48 g, 2 mmol) were placed in a 100-mL, round-bottom flask. Following the addition of DDQ (0.68 g, 3 mmol), the reaction flask was completely wrapped in aluminum foil and stirred at 20 °C for 1 h. The reaction mixture turned from dark green to a dark shade of red. The mixture was diluted with DCM and vacuum-filtered through a coarse glass frit with Celite. The collected filtrate was washed with saturated NaHCO_3_ solution (40 mL), swirling gently to mix. The organic layer was washed again with NaHCO_3_ (80 mL) and swirled vigorously to mix. Again, the organic layer was washed with NaHCO_3_ (2 × 100 mL), and then shaken to combine. The resulting organic solution was dried with anhydrous Na_2_SO_4_ before concentration under reduced pressure. The crude product was purified on a Biotage flash purification system (hexane:EtOAc) to yield the desired product as a clear, colorless oil with an 84% yield (refer to Table S11 for gradient); [α]_D_^25^ −21 (*c* 0.024, CHCl_3_); R_f_ = 0.36 (Hexane:EtOAc, 8:2); ^1^H NMR (500 MHz, CDCl_3_) δ 5.39–5.29 (m, 4H), 5.08 (p, *J* = 5.0 Hz, 1H), 4.32 (dd, *J* = 11.9, 4.5 Hz, 1H), 4.23 (dd, *J* = 12.0, 5.7 Hz, 1H), 3.72 (dd, *J* = 5.0, 1.6 Hz, 2H), 2.33 (dt, *J* = 11.1, 7.5 Hz, 4H), 2.05–1.96 (m, 8H), 1.62 (h, *J* = 7.3 Hz, 5H), 1.37–1.20 (m, 42H), 0.88 (t, *J* = 6.9 Hz, 6H). ^13^C NMR (126 MHz, CDCl_3_) δ 173.8, 173.4, 130.0, 129.7, 77.3, 77.0, 76.8, 72.1, 62.0, 61.6, 34.3, 34.1, 31.9, 29.8, 29.7, 29.5, 29.3, 29.2, 29.1, 29.1, 27.2, 27.2, 24.9, 24.9, 22.7, 14.1. QTOF-HRMS (ESI) for C_39_H_72_O_5_ [M+H^+^]: found 621.5452, calcd 621.5452.

*(S)-1-Hydroxy-3-(palmitoyloxy)propan-2-yl oleate (8a).* White semi-solid in 81% yield; [α]_D_^25^ −27.2 (*c* 0.021, CHCl_3_); R_f_ = 0.36 (Hexane:EtOAc, 8:2); ^1^H NMR (500 MHz, CDCl_3_) δ 5.40–5.29 (m, 2H), 5.08 (p, *J* = 5.0 Hz, 1H), 4.32 (dd, *J* = 11.9, 4.5 Hz, 1H), 4.23 (dd, *J* = 11.9, 5.6 Hz, 1H), 3.73 (dd, *J* = 5.0, 1.6 Hz, 2H), 2.33 (dt, *J* = 11.4, 7.5 Hz, 4H), 2.01 (q, *J* = 6.3 Hz, 4H), 1.62 (h, *J* = 7.6 Hz, 4H), 1.40–1.18 (m, 46H), 0.88 (t, *J* = 6.9 Hz, 6H). ^13^C NMR (126 MHz, CDCl_3_) δ 173.8, 173.4, 130.1, 129.7, 77.3, 77.0, 76.8, 72.1, 62.0, 61.6, 34.3, 34.1, 31.9, 29.8, 29.7, 29.7, 29.6, 29.5, 29.5, 29.4, 29.3, 29.3, 29.2, 29.1, 29.1, 27.2, 27.2, 24.9, 24.9, 22.7, 14.1. QTOF-HRMS (ESI) for C_37_H_70_O_4_ [M+H^+^]: found 595.5296, calcd 595.5295.

### 3.7. Synthesis of (R)-2,3-Bis(oleoyloxy)propyl phosphonate (9b)

The synthesis of **9** was performed according to a previously reported protocol [20]. Alcohol **8b** (0.248 g, 0.4 mmol) was placed in an oven-dried, 10-mL, two-neck, round-bottom flask equipped with a stir bar. The flask was then dried using Schlenk techniques followed by the attachment of an Ar balloon. The starting material was then dissolved in dry pyridine (4 mL) and the solution was cooled to 0 °C in an ice bath. Diphenyl phosphite (0.46 mL, 2.5 mmol) was added dropwise and the solution was stirred for 1 h. The solution was then allowed to warm to room temperature where a 1:1 H_2_O:Et_3_N solution (5 mL) was added to the round-bottom flask, where it was stirred for an additional 1 h. The pyridine was removed from the solution under reduced pressure by azeotropically drying three times with toluene. The crude oil was then dissolved with DCM (30 mL) and washed with saturated NaHCO_3_ (3 × 15 mL), where it was dried with anhydrous Na_2_SO_4_ and concentrated under reduced pressure. The product was purified using a silica gel column to yield a white semi-solid with 75% yield (gradient from 100:0 to 95:5 CHCl_3_:MeOH containing 0.5% Et_3_N); [α]_D_^27^ +25.5 (*c* 0.032, CHCl_3_); R_f_ = 0.23 (Hexane:EtOAc, 8:2); ^1^H NMR (500 MHz, CDCl_3_) δ 5.37–5.28 (m, 4H), 5.20 (qd, *J* = 5.3, 3.6 Hz, 1H), 4.35 (dd, *J* = 11.9, 3.7 Hz, 1H), 4.16 (dd, *J* = 11.9, 6.3 Hz, 1H), 4.00 (dd, *J* = 8.1, 5.2 Hz, 2H), 3.06 (qd, *J* = 7.3, 4.2 Hz, 6H), 2.32–2.23 (m, 4H), 1.99 (qd, *J* = 6.2, 2.7 Hz, 8H), 1.58 (tq, *J* = 7.0, 3.6 Hz, 4H), 1.37–1.19 (m, 50H), 0.86 (t, *J* = 6.9 Hz, 6H). ^13^C NMR (126 MHz, CDCl_3_) δ 173.4, 173.0, 130.0, 129.7, 77.3, 77.1, 76.8, 70.3, 70.3, 62.4, 62.1, 62.1, 45.6, 34.3, 34.1, 31.9, 29.8, 29.7, 29.5, 29.3, 29.2, 29.2, 29.1, 27.2, 27.2, 24.9, 22.7, 14.1, 8.5. ^31^P NMR (203 MHz, CDCl_3_) δ 4.55. QTOF-HRMS (ESI) for C_39_H_72_O_7_P [M+Na^+^]: found 707.4986, calcd 707.4986.

*(R)-2-(Oleoyloxy)-3-(palmitoyloxy)propyl phosphonate (9a).* White semi-solid in 78% yield [α]_D_^27^ +18.5 (*c* 0.029, CHCl_3_); R_f_ = 0.23 (Hexane:EtOAc, 8:2); ^1^H NMR (500 MHz, CDCl_3_) δ 5.38–5.29 (m, 2H), 5.21 (qd, *J* = 5.2, 3.6 Hz, 1H), 4.36 (dd, *J* = 11.9, 3.8 Hz, 1H), 4.17 (dd, *J* = 11.9, 6.3 Hz, 1H), 4.03 (dd, *J* = 8.2, 5.1 Hz, 2H), 3.08 (qd, *J* = 7.3, 4.4 Hz, 5H), 2.30 (dt, *J* = 9.9, 7.6 Hz, 4H), 2.04–1.96 (m, 4H), 1.59 (h, *J* = 6.7 Hz, 5H), 1.40–1.19 (m, 55H), 0.87 (t, *J* = 6.9 Hz, 7H); ^13^C NMR (126 MHz, CDCl_3_) δ 173.4, 173.00, 130.0, 129.7, 77.3, 77.0, 76.8, 70.2, 62.3, 45.6, 34.3, 34.1, 32.0, 29.8, 29.8, 29.7, 29.7, 29.5, 29.4, 29.3, 29.2, 29.2, 29.1, 27.2, 27.2, 24.9, 22.7, 14.1, 8.6; ^31^P NMR (203 MHz, CDCl_3_) δ 4.59. QTOF-HRMS (ESI) for C_37_H_70_O_7_P [M+Na^+^]: found 681.4826, calcd 681.4829.

### 3.8. Synthesis of (2R)-3-(((S)-2,2-dimethyl-1,3-dioxolan-4-yl)methoxy)propane-1,2-dioleyl phosphonate (11b)

Compound **9b** (0.235 g, 0.3 mmol) was added to an oven-dried, 10-mL, two-neck, round-bottom flask equipped with a stir bar. The flask was cycled three times with vacuum/Ar, and a balloon was attached. Dry pyridine (5 mL) was then added, followed by solketal (0.05 mL, 0.4 mmol). The reaction temperature was lowered to 0 °C via an ice bath and pivaloyl chloride (0.22 mL, 1.7 mmol) was subsequently added dropwise to the reaction. The solution changed from transparent to a violet. The reaction was stirred for 1 h and then warmed slowly to room temperature. Pyridine was then azeotropically stripped from the solution with toluene under reduced pressure. The crude oil was then redissolved in DCM (30 mL), and the solution was washed with saturated NaHCO_3_ (2 × 10 mL). The organic layer was then dried with anhydrous Na_2_SO_4_ and concentrated under reduced pressure. The crude product was purified on a silica gel column to yield a colorless oil (gradient of 100:0 to 98:2 DCM:MeOH); [α]_D_^26^ +12.9 (*c* 0.023, CHCl_3_); R_f_ = 0.34 (Hexane:EtOAc, 8:2); ^1^H NMR (500 MHz, CDCl_3_) δ 5.39–5.29 (m, 4H), 5.23 (h, *J* = 5.2 Hz, 1H), 4.38–4.00 (m, 10H), 3.85–3.70 (m, 2H), 2.37–2.27 (m, 4H), 2.05–1.97 (m, 8H), 1.61 (h, *J* = 7.1 Hz, 4H), 1.44 (d, *J* = 3.5 Hz, 4H), 1.39–1.20 (m, 51H), 0.88 (t, *J* = 6.8 Hz, 6H); ^13^C NMR (126 MHz, CDCl_3_) δ 173.8, 173.4, 130.1, 129.7, 77.3, 77.0, 76.8, 72.1, 62.0, 61.6, 34.3, 34.1, 31.9, 29.8, 29.7, 29.7, 29.6, 29.5, 29.5, 29.4, 29.3, 29.3, 29.2, 29.1, 29.1, 27.2, 27.2, 24.9, 24.9, 22.7, 14.1; ^31^P NMR (203 MHz, CDCl_3_) δ 8.62, 8.52 QTOF-HRMS (ESI) for C_45_H_83_O_9_P [M+Na^+^]: found 821.5666, calcd 821.5667.

*(2R)-1-(((S)-2,2-Dimethyl-1,3-dioxolan-4-yl)methoxy)(hydroxy)-3-(palmitoyloxy)propan-2-oleyl phosphonate (11a)*. Clear oil in 72% yield; [α]_D_^26^ −1.90 (*c* 0.023, CHCl_3_); R_f_ = 0.35 (Hexane:EtOAc, 8:2); ^1^H NMR (500 MHz, CDCl_3_) δ 5.39–5.29 (m, 2H), 5.26–5.20 (m, 1H), 4.37–4.00 (m, 8H), 3.85–3.70 (m, 2H), 2.39–2.27 (m, 4H), 2.05–1.95 (m, 4H), 1.67–1.55 (m, 4H), 1.46–1.14 (m, 54H), 0.88 (t, *J* = 6.8 Hz, 6H). ^13^C NMR (126 MHz, CDCl_3_) δ 173.4, 173.0, 130.0, 129.7, 77.3, 77.1, 76.8, 70.3, 70.3, 62.4, 62.1, 62.1, 45.6, 34.3, 34.1, 31.9, 29.8, 29.7, 29.5, 29.3, 29.2, 29.2, 29.1, 27.2, 27.2, 24.9, 22.7, 14.1; ^31^P NMR (203 MHz, CDCl_3_) δ 8.63, 8.52. QTOF-HRMS (ESI) for C_43_H_81_O_9_P [M+Na^+^]: found 795.5512, calcd 795.5510.

### 3.9. Synthesis of (2R)-3-((((S)-2,3-dihydroxypropoxy)(hydroxy)phosphoryl)oxy)propane-1,2-diyl dioleate (1)

H-phosphonate **11** (0.160 g, 0.20 mmol) was added to a 10-mL, round-bottom flask. A 9:1 *v*/*v* of H_2_O/Pyr. (5 mL) was added to flask, and the temperature was lowered to 0 °C. I_2_ was then added, and the reaction was warmed to 24 °C with stirring for 3 h. The pyridine was then removed from the solution by azeotropically drying three times with toluene. The resulting oil was diluted with 45 mL of DCM and washed with a solution of saturated Na_2_SO_3_ (2 × 10 mL) and once with brine (10 mL). The organic layer was then dried with anhydrous Na_2_SO_4_ and concentrated under reduced pressure. The crude product was purified on a manual silica gel column (gradient of 98:2 CHCl_3_:MeOH to 65:25:4 CHCl_3_:MeOH:H_2_O) (refer to Table S4 for gradient). Fractions were collected in 13 × 100 mm cell culture tubes, with 72 mL intervals between each change, in a mobile phase composition (9 mL/fraction). Product appeared from fractions 25–32. The isolated product was concentrated under reduced pressure in a 20 mL scintillation vial, and then CHCl_3_ (5 mL) was added to dissolve the oil. After cooling the solution to 0 °C in an ice bath, MeOH (0.1 mL) and TFA (0.5 mL dropwise) were added to the reaction mixture. The reaction was allowed to stir for 30 min at room temperature. Saturated NaHCO_3_ was then added, and the solution was diluted with CHCl_3_ (30 mL). The mixture was transferred to a separatory funnel, where MeOH (20 mL) and then H_2_O (10 mL) were added, followed by shaking. The organic layer was collected, dried with anhydrous Na_2_SO_4_, and concentrated under reduced pressure. The crude product was then purified using a gradient of 98:2 CHCl_3_:MeOH to 65:25:4 CHCl_3_:MeOH:H_2_O to yield a clear oil in 73% yield. [α]_D_^27^ −16 (*c* 0.011, CHCl_3_); R_f_ = 0.46 (CHCl_3_:MeOH:H_2_O, 65:25:4); ^1^H NMR (500 MHz, CDCl_3_) δ 5.26 (tt, *J* = 5.7, 3.3 Hz, 4H), 5.12 (qd, *J* = 5.8, 3.0 Hz, 1H), 4.28 (dt, *J* = 12.1, 3.1 Hz, 1H), 4.10–4.00 (m, 1H), 3.93–3.75 (m, 5H), 3.66–3.50 (m, 3H), 3.43 (d, *J* = 6.0 Hz, 5H), 2.23 (td, *J* = 8.8, 4.8 Hz, 4H), 1.93 (q, *J* = 7.1 Hz, 8H), 1.51 (td, *J* = 7.9, 3.9 Hz, 4H), 1.33–1.14 (m, 42H), 0.80 (td, *J* = 6.9, 3.0 Hz, 6H). ^13^C NMR (126 MHz, CDCl_3_) δ 174.0, 173.8, 162.5, 130.0, 129.6, 117.5, 77.3, 77.1, 76.8, 70.4, 62.5, 62., 49.5, 49.4, 49.2, 49.0, 48.9, 48.7, 48.5, 34.1, 33.9, 31.8, 29.7, 29.4, 29.2, 29.2, 29.1, 29.0, 29.00, 27.1, 27.1, 24.7, 22.6, 14.0, 0.9. ^31^P NMR (203 MHz, CDCl_3_) δ -2.67. QTOF-HRMS (ESI) for C_42_H_79_O_10_P [M+Na^+^]: found 797.5300, calcd 797.5303.

*(2R)-1-((((S)-2,3-Dihydroxypropoxy)(hydroxy)phosphoryl)oxy)-3-(palmitoyloxy)propan-2-yl oleate* (2). A waxy solid in 70% yield. [α]_D_^27^ −7.4 (*c* 0.088, CHCl_3_); ^1^H NMR (500 MHz, CDCl_3_) δ 5.39–5.28 (m, 2H), 5.17 (s, 1H), 4.38 (d, *J* = 11.4 Hz, 1H), 4.11 (dd, *J* = 12.6, 6.8 Hz, 1H), 3.98–3.74 (m, 5H), 3.73–3.50 (m, 3H), 2.37–2.21 (m, 4H), 2.00 (q, *J* = 6.5 Hz, 4H), 1.66–1.49 (m, 4H), 1.38–1.14 (m, 43H), 0.88 (t, *J* = 6.9 Hz, 6H). ^13^C NMR (126 MHz, CDCl_3_) δ 174.2, 174.1, 130.0, 129.7, 117.8, 115.4, 77.3, 77.0, 76.8, 70.7, 62.8, 34.2, 34.1, 32.0, 31.9, 29.8, 29.8, 29.7, 29.7, 29.6, 29.4, 29.4, 29.3, 29.3, 29.2, 27.3, 24.9, 24.8, 22.7, 14.1, 1.0. ^31^P NMR (203 MHz, CDCl_3_) δ 0.93. QTOF-HRMS (ESI) for C_40_H_77_O_10_P [M+Na^+^]: found 771.5145, calcd 771.5146.

## Data Availability

Not applicable.

## Supplementary Materials

The following supporting information can be downloaded at: https://www.mdpi.com/article/10.3390/molecules27072199/s1, Discussions and procedures on high-throughput experimentation, flow chemical methods, all other efforts towards the synthesis of PG via phosphoramidites (Figures S1–S14) (Tables S1–S3) (Scheme S1), gradient tables for FPLC methods (Tables S4–S8), and NMR data (Figures S15–S34).

## Sample Availability

Samples of the compounds are available from the authors.
