# Supplementary material for: Synthesis of Phosphatidyl Glycerol Containing Unsymmetric Acyl Chains Using H-Phosphonate Methodology
Source: Molecules. 2022 Mar 28;27(7):2199. doi: 10.3390/molecules27072199 (PMC9000858; doi:10.3390/molecules27072199)
Supplement: Supplementary file 1 [file molecules-27-02199-s001.zip › molecules-1628039-supplementary.pdf]

molecules

MDPI

Article

# Synthesis of Phosphatidyl Glycerol Containing Unsymmetric Acyl Chains Using H-Phosphonate Methodology

**Zachary J. Struzik [1], Shruti Biyani [1], Tim Grotzer [1], Judith Storch [2] and David H. Thompson [1,*]**

1 Bindley Bioscience Center, Department of Chemistry, Multi-disciplinary Cancer Research Facility, Purdue University, 1203 W. State Street, West Lafayette, IN 47907, USA; zstruzik@purdue.edu (Z.J.S.); biyanis@purdue.edu (S.B.); tgrotzer@purdue.edu (T.G.)
2 Department of Nutritional Sciences and Rutgers Center for Lipid Research, Rutgers University, New Brunswick, NJ 08901, USA; storch@sebs.rutgers.edu
* Correspondence: davethom@purdue.edu

## Table of Contents

Initial Studies to Simultaneously Deprotect the Cyanoethyl Groups and Silyl Groups……………………………… S2
High-throughput Experimentation of Diphenylmethylsilyl Deprotection…………………………………………….. S4
Development of Phosphorylated Intermediates via High-throughput Experimentation and Flow Chemistry…… S8
Milligram Scale Headgroup Phosphorylation Reactions in Flow……………………………………………………….. S13
Gram Scale Headgroup Phosphorylation Reactions in Flow…………………………………………………………….. S16
Phosphorylation of Glycerol Backbone………………………………………………………………………………… S19
Methods………………………………………………………………………………………………………………… S20
Gradient Tables Used for FPLC……………………………………………………………………………………… S24
NMR of All Compounds……………………………………………………………………………………………… S26

**Initial studies to simultaneously deprotect the cyanoethyl groups and silyl groups**

Initial attempts to improve the phosphorylation conditions began by exploring more polar fluoride sources to deprotect the TBS ethers in an attempt to simplify the product isolation procedure (Table S1). Many of the organic and inorganic naked fluorine sources we tested led to a low conversion of the starting material (e.g., $Me_4NF$[25], $KF{\cdot}H_2O$[27], or NaF[26]). Interestingly, $^1H$ and $^{31}P$ NMR revealed that the cyanoethyl-protecting group remained intact during TBS deprotection efforts with KF·H2O and 18-crown-6 ether. Unfortunately, similar purification issues to TBAF arose, since mobile phases in phospholipid chromatography are typically aqueous ion-pairing media such as $CHCl_3$:MeOH:aq. $NH_4Cl$[14], which can lead to the streaking of compounds such as crown ethers on polar stationary phases. Since our goal was to create a route with a simplified purification scheme, we sought a different approach.

**Table S1.** Summary of attempts to perform a global deprotection step with alternative fluoride sources. [a] Conversion of starting material (SM), qualitatively determined by TLC.

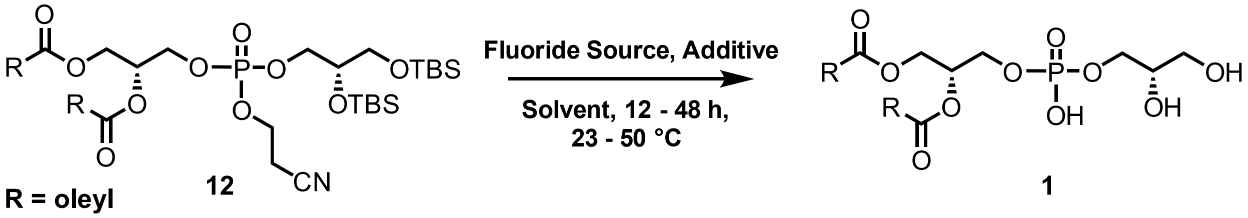


| Entry | Fluoride Source | Solvent | Additive | Time (h) | Temperature (°C) | Conversion of SM[a] |
|---|---|---|---|---|---|---|
| 1 | NaF | 3:2:1 THF:$H_2O$:MeOH | AcOH | 24 | 23 | None |
| 2 | TMAF | 3:1 THF:MeOH | AcOH | 12 | 23 | None |
| 3 | TMAF | 3:1 THF:MeOH | AcOH | 48 | 23 | None |
| 4 | KF•2$H_2O$ | 1.5:1 THF:MeOH | - | 24 | 23 | Slight |
| 5 | KF•2$H_2O$ | 1.5:1 THF:MeOH | AcOH | 12 | 23 | Slight |
| 6 | KF•2$H_2O$ | 1.5:1 THF:MeOH | AcOH | 24 | 35 | Slight |
| 7 | KF•2$H_2O$ | 1.5:1 THF:MeOH | AcOH + 18-c-6 | 36 | 23 | Slight |
| 8 | Benzylsulfonyl Fluoride | 4:1 THF:$H_2O$ | - | 12 | 23 | No |
| 9 | Benzylsulfonyl Fluoride | 4:1 THF:$H_2O$ | - | 12 | 50 | No |

**12** (0.1 g, 0.95 mmol) and fluoride source (19 mmol) were placed in a 25-mL, round-bottom flask equipped with a magnetic stir bar. The contents were dissolved in the specified solvent (10 mL) and the additive was added (19 mmol). Reactions conducted above 23 °C were heated using a temperature-controlled oil bath. Reaction times varied between 12 and 48 h. The conversion of starting material was monitored by the disappearance of **12**, which was synthesized according to a previously published protocol.[9] Upon the initial investigation of naked fluorine sources, utilized to simultaneously deprotect both the silyl groups as well as the cyanoethyl group of the phosphate, the solubility of both the substrate and the fluorine salt proved to be a challenge. For example, an inorganic salt such as NaF was not soluble in any of the tested aqueous/organic solvent systems. On the other hand, the substrate was also not soluble in a pure aqueous system. This led to no conversion of the starting material as determined by TLC. Even the addition of an organic acid in a purely aqueous system led to precipitation of the salt. Due to the risk of acyl chain migration of the phosphoglycerol backbone to the glycerol head group, we avoided heating these reaction mixtures to high temperatures (> 50 °C). However, gentle heating of the solution below this temperature still appeared to be ineffective for global deprotection. To alleviate this issue, we hypothesized that using a less-hydrophobic ammonium fluoride such as tetramethyl ammonium fluoride (TMAF), instead of TBAF, would not only improve the solubility of the fluoride source in organic solvents, but also might make

the separation of the product and TMAF more facile. Unfortunately, similar solubility issues arose between the substrate and TMAF, which forced us to explore other fluoride reagents.

While NaF has shown little solubility in organic/aqueous systems, KF·2$H_2O$ has shown moderate solubility in the same solvent ratios. We were able to observe conversion of the substrate by TLC and were able to isolate the product after column chromatography. The silyl groups were still present on our substrate, as observed by the $^1$H NMR shifts between 0.87-0.81 and 0.07 – 0.02 ppm (Figure S1). However, the $\alpha$ protons in the cyano group of the phosphotriester had disappeared. Additionally, the absence of the cyanotheyl carbon peaks corresponding to the cyano carbon, as well as the $\alpha$ carbon at ~19 and 117 ppm, respectively, indicated that cyanoethyl group was removed. Finally, $^{31}$P NMR revealed a single peak at -0.54 ppm, which can be attributed to a phosphate diester. Encouraged by these results, we attempted to remove the silyl group by adding a weak acid, such as acetic acid, to promote the hydrolysis of the silyl groups. Unfortunately, the addition of AcOH hindered the solubility, thereby limiting the hydrolysis to only one of the silyl groups. It has been reported that the use of crown ethers can help the solubility of the KF salt. While the solubility of the substrate and salt appeared to increase upon addition of the crown ether in the presence of an acid, the conversion of the substrate did not appear to increase.

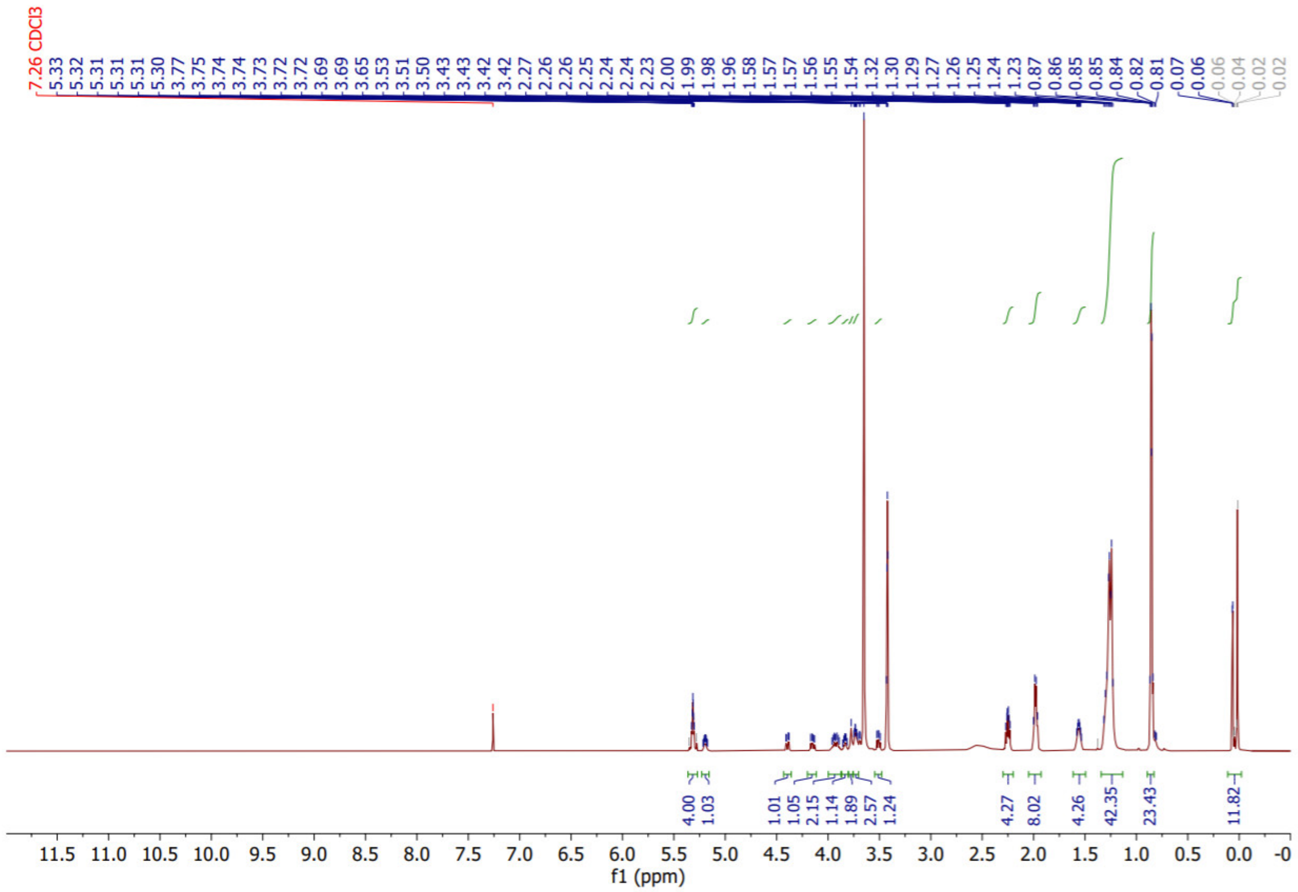


(A)

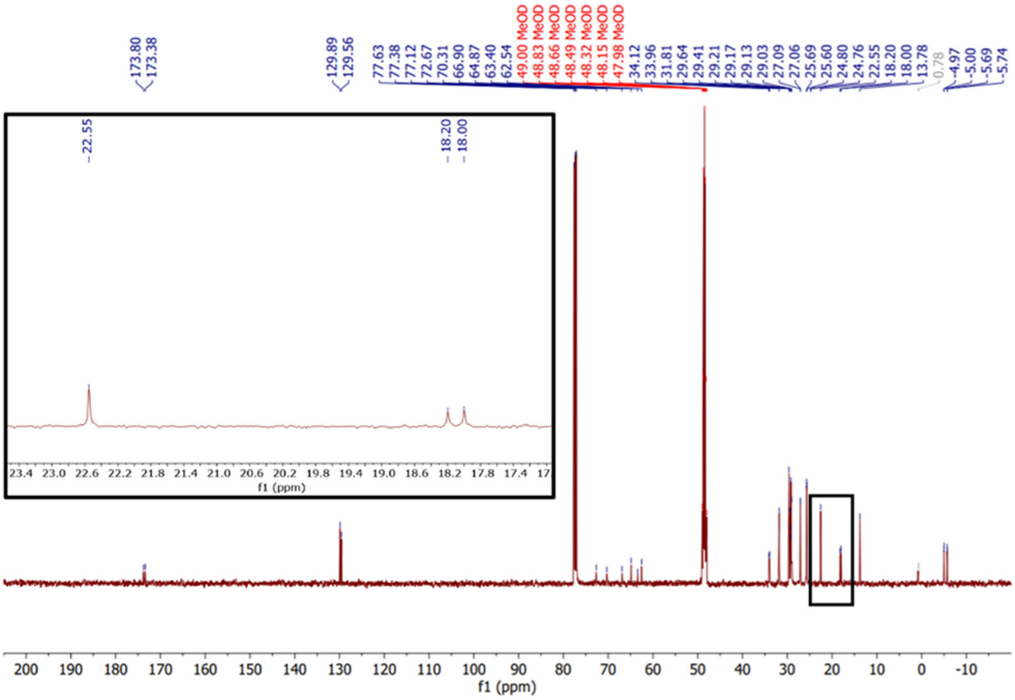


(B)

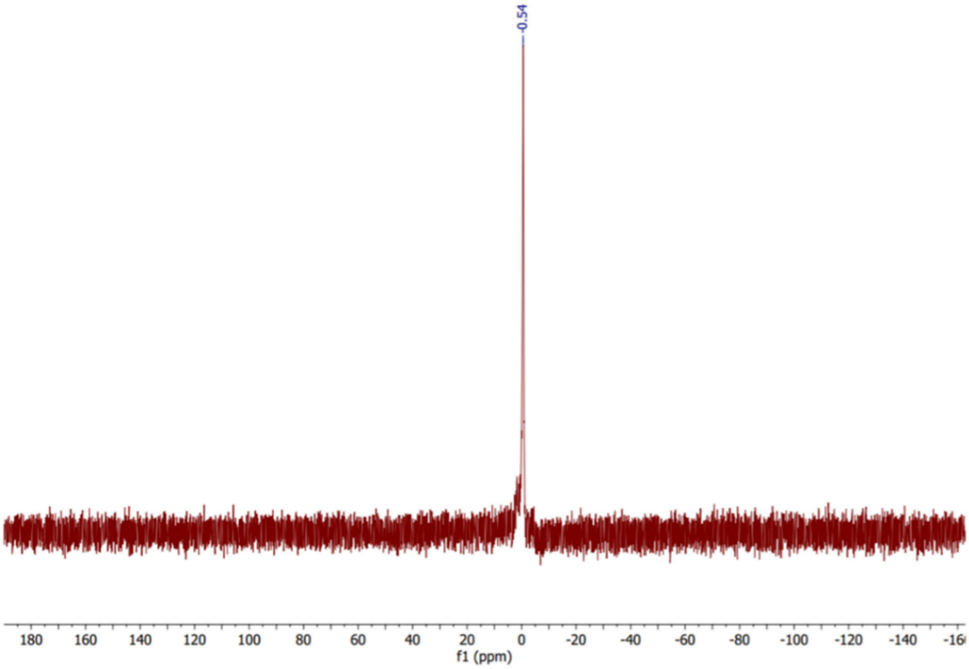


(C)

**Figure S1.** $^{1}H$ (**A**), $^{13}C$ (**B**), and $^{31}P$ (**C**) NMR of partially converted substrate by $KF{\cdot}2H_2O$.

## High-throughput Experimentation of Diphenylmethylsilyl Deprotection

Catalytic amounts of sulfonyl fluoride can reportedly selectively deprotect diphenyl methyl silyl (DPMS) ethers in the presence of an aqueous surfactant such as TPGS 750-M.[28] Unfortunately, these conditions failed to deprotect our TBS modified substrate under surfactant-free conditions (i.e., we did not want to further complicate product chromatographic purification). We then replaced the headgroup protection to enable the use of DPMS ethers, instead of TBS ethers, to accommodate the use of benzylsulfonyl fluoride

(BSF) as a deprotection agent (Table S2). Before applying this strategy to the final deprotection steps, we optimized the deprotection conditions using a model substrate-**13**-bearing DPMS-protecting groups published according to a previously reported protocol using DPMSCl instead of TBSCl[9], and a PMB group that was expected to remain inert to the BSF. Upon completion of pilot experiments, we observed trace amounts of **14** under almost all conditions. Entry 1 seemed to be the only set of conditions that resulted in no reaction, despite these conditions being optimal in the substrates tested by Akproji et al. [5].

**Table S2.** Initial attempts to remove DPMS silyl ethers using BSF.

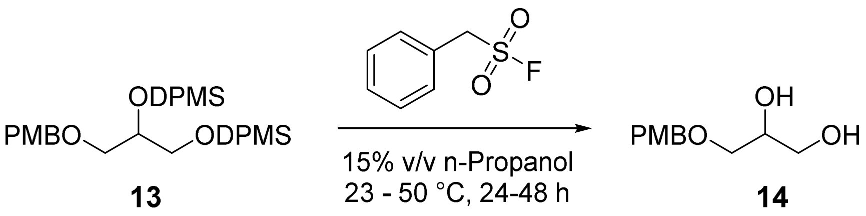


| Entry | Eq. BSF | TPGS 750-M | Solvent | Time (h) | Temp. (⁰C) | Conversion of 3[a] |
|---|---|---|---|---|---|---|
| 1 | 0.3 | Yes | $H_2O$ | 48 | 50 | No |
| 2 | 0.5 | No | THF | 24 | 23 | Slight |
| 3 | 0.5 | No | THF | 48 | 50 | Slight |
| 4 | 0.3 | No | THF | 24 | 23 | Slight |
| 5 | 0.3 | No | THF | 48 | 50 | Slight |
| 6 | 1 | No | THF | 24 | 23 | Slight |
| 7 | 1 | No | THF | 48 | 50 | Slight |

We then chose to explore the larger reaction landscape needed to identify optimal reaction conditions. We turned to a recently reported high-throughput experimentation system (HTE) to rapidly screen reaction conditions using a liquid-handling robot, in tandem with desorption electrospray ionization mass spectrometry (DESI-MS) (Figure S2).[29] From this experiment, we were able to examine 84 unique reaction conditions, including seven solvents and six BSF catalyst stoichiometries (Figure S3). In this semi-automated system, we were able to simultaneously examine each set of reaction conditions at 20 °C and 50 °C in sealed, 96-well microtiter plates. The results of this experiment indicate that the examined temperatures do not play a significant role in the reaction progress. They do, however, indicate that catalytic amounts of BSF appear to be more efficient than stoichiometric amounts of reagents. Additionally, a 3:1 THF/water solution appeared to be far superior to many of the other tested solvent systems, although the organic/water solvent systems generally seemed to outperform the purely organic solvents. Armed with these preferred conditions, identified by HTE, we attempted the synthesis of the newly designed phosphoglycerol headgroup. Unfortunately, standard deprotection conditions of PMB ether **13** with DDQ[32] produced an acidic hemiacetal byproduct that promoted hydrolysis of the DPMS-ether-protecting groups of **13** (Scheme S1A), despite the modifications to the protocol with respect to time, temperature, and DDQ stoichiometry. Even when implementing protocols that suggested more mild approaches, such as the use of an Ag(I) catalyst in the presence of 1,3,5-trimethoxybenzene[31], we were unable to produce the desired product. Fortunately, we were able to circumvent this issue by replacing the PMB ether with a phenyl acyl ester that could be cleared by DIBAL-H reduction (Scheme S1B). We were able to obtain **18** with 88% yield by reacting **17** with phenylacetyl chloride in the presence of $Et_3N$. The acetonide was successfully removed to afford **19** using a 2M HCl/THF solution with 94% yield, without significant hydrolysis of the ester. Installation of the DPMS groups to provide **20** with an 89% yield was carried out by using

DPMSCl in the presence of imidazole and $Et_3N$. Finally, **21** was afforded with 79% yield after DIBAL-H treatment.

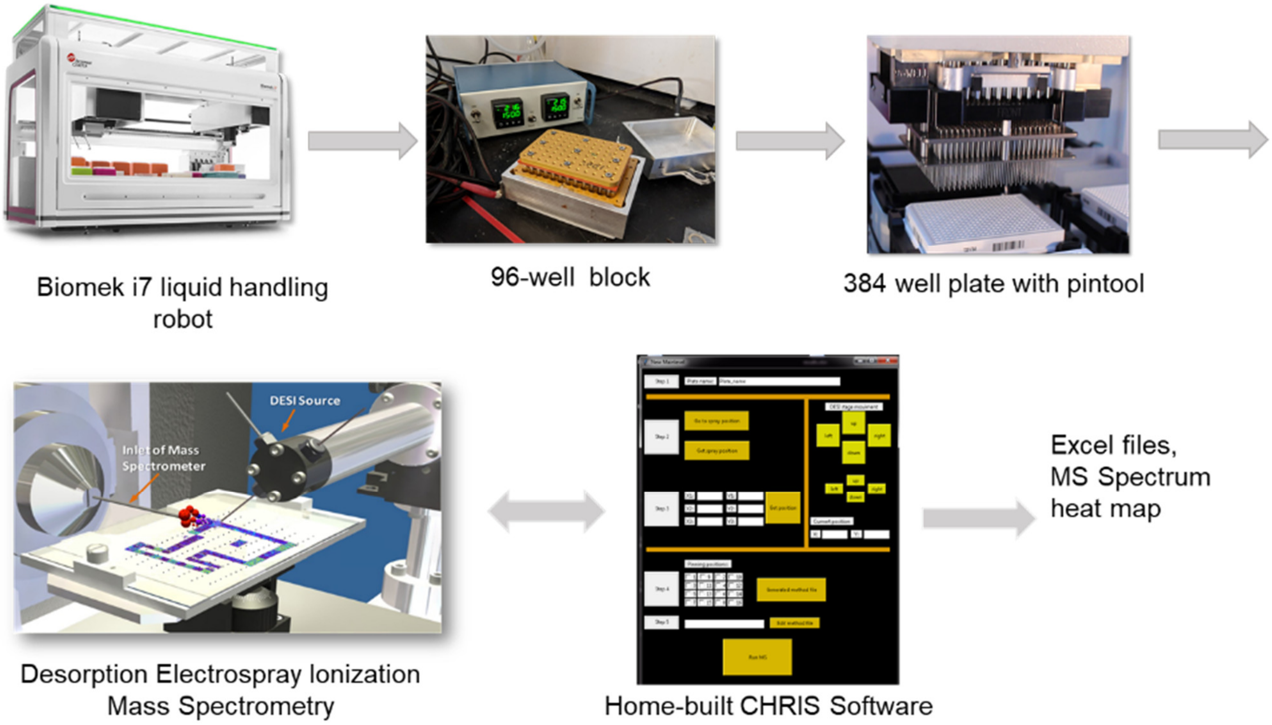


**Figure S2.** General workflow for High-Throughput Experimentation.

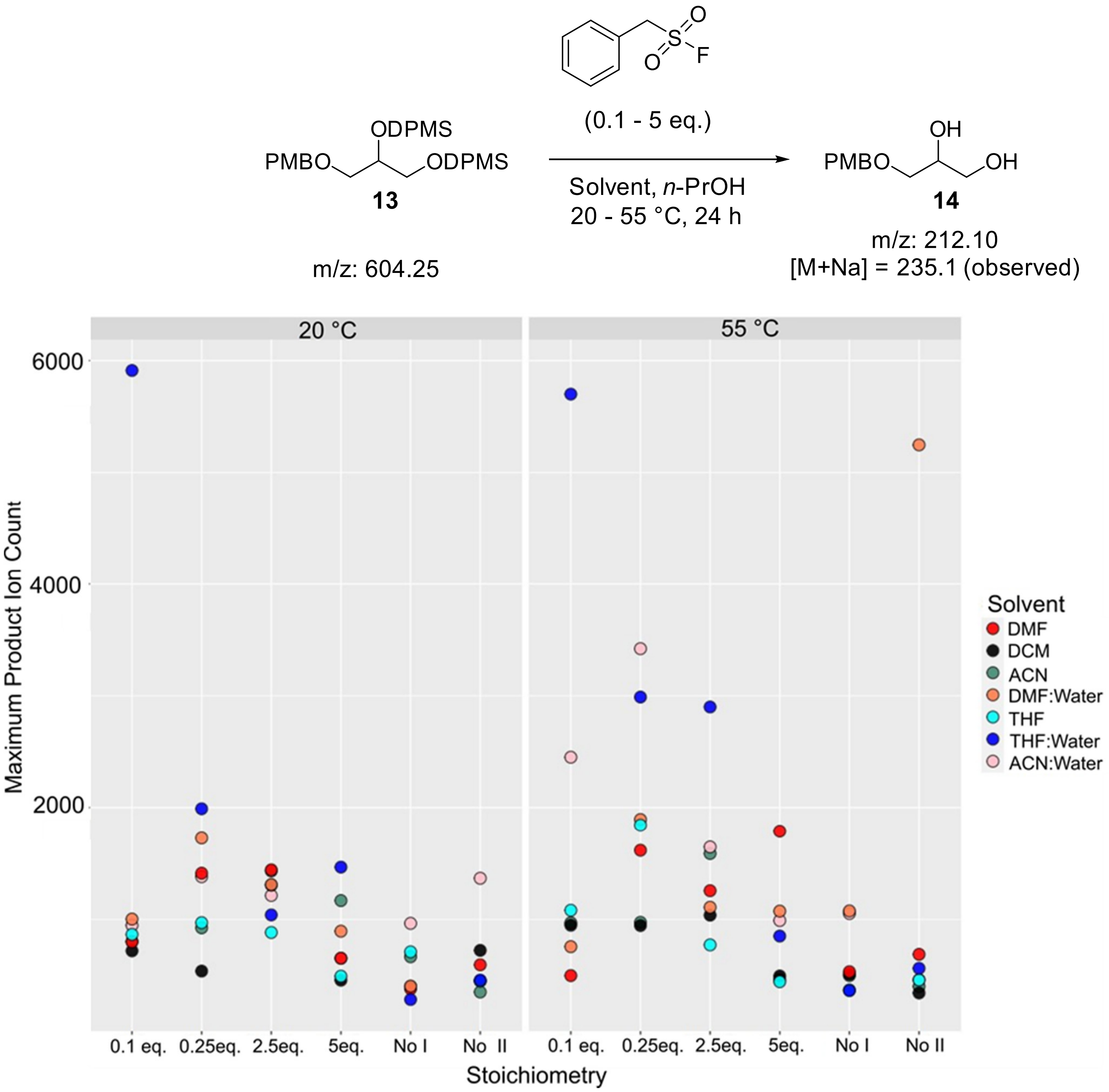


**Figure S3.** the screening of DPMS ether deprotection of BSF. The y-axis is the average maximum product ion intensity for each reaction. Each set of conditions was run in quadruplicates. The x-axis is the BSF catalyst loadings. “No I” and “No II” refer to negative controls, where reactions contain no substrate or no catalyst, respectively.

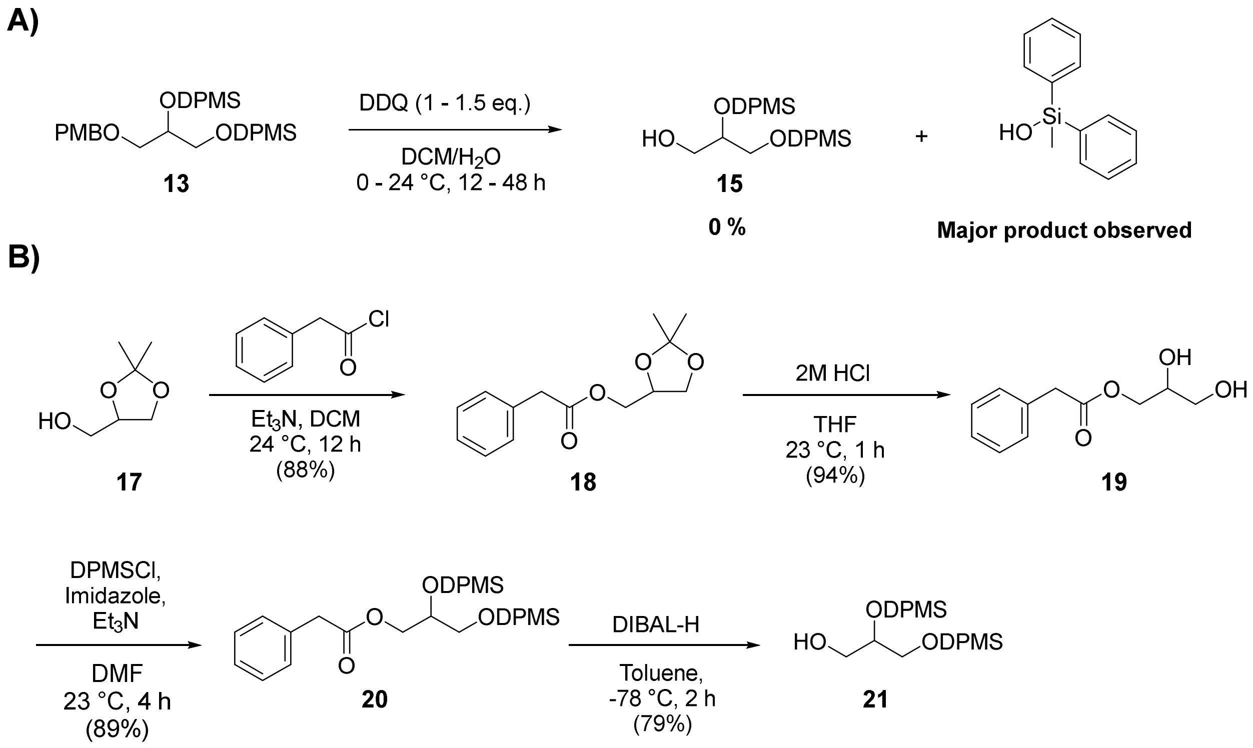


**Scheme 1.** (**A**) Hydrolysis of DPMS ethers upon reaction with DDQ. (**B**) Alternative protection/deprotection strategy employing a solketal phenyl ester intermediate.

### Development of Phosphorylated Intermediates via High-throughput Experimentation and Flow Chemistry

Before employing this transformation in flow, we deployed a round of HTE to determine the most promising reaction conditions. Ideally, we preferred the use of the asymmetric NCP reagent and rationalized that enhanced mixing in flow might not only improve the scaling issues, but also reduce the number of hydrolyzed byproducts, since the phosphoramidite could be run closer to the 1:1 stoichiometry than the 2:1 stoichiometry that we previously used. In this experiment, we used a $2^3$ factorial design of experiments with time, base, and phosphoramidite stoichiometry as variables (Figures S4 ad S5). Interestingly, our desired mass peak from phosphoramidite **22** at an *m*/*z* of 684.3 did not appear in any of our reactions. Instead, we observed a significant amount of an *m*/*z* of 239.1 corresponding to a methoxylated phosphotriester. From these findings, we can infer that **22** was undergoing substitution with methanol and cleaving the DPMS ethers in the acidic DESI-MS spray solvent. This hypothesis is consistent with Kele et al.[30] and supported by synthesizing **22** and observing a similar spectrum to that of the HTE experiment (Figure S7), suggesting that **22** degrades under the conditions of the DESI-MS analysis.

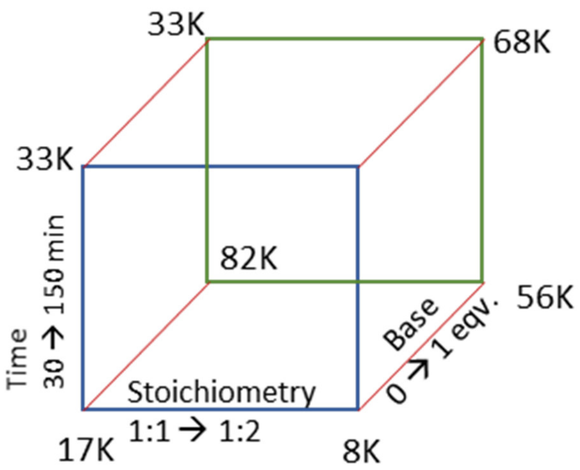


**Figure S4.** $2^3$ full factorial design with time, stoichiometry (where phosphoramidite is in excess), and base at lower and higher values. The numbers on the edge of the cube (17K, 33K, etc.) correspond to the DESI-MS average ion count response.

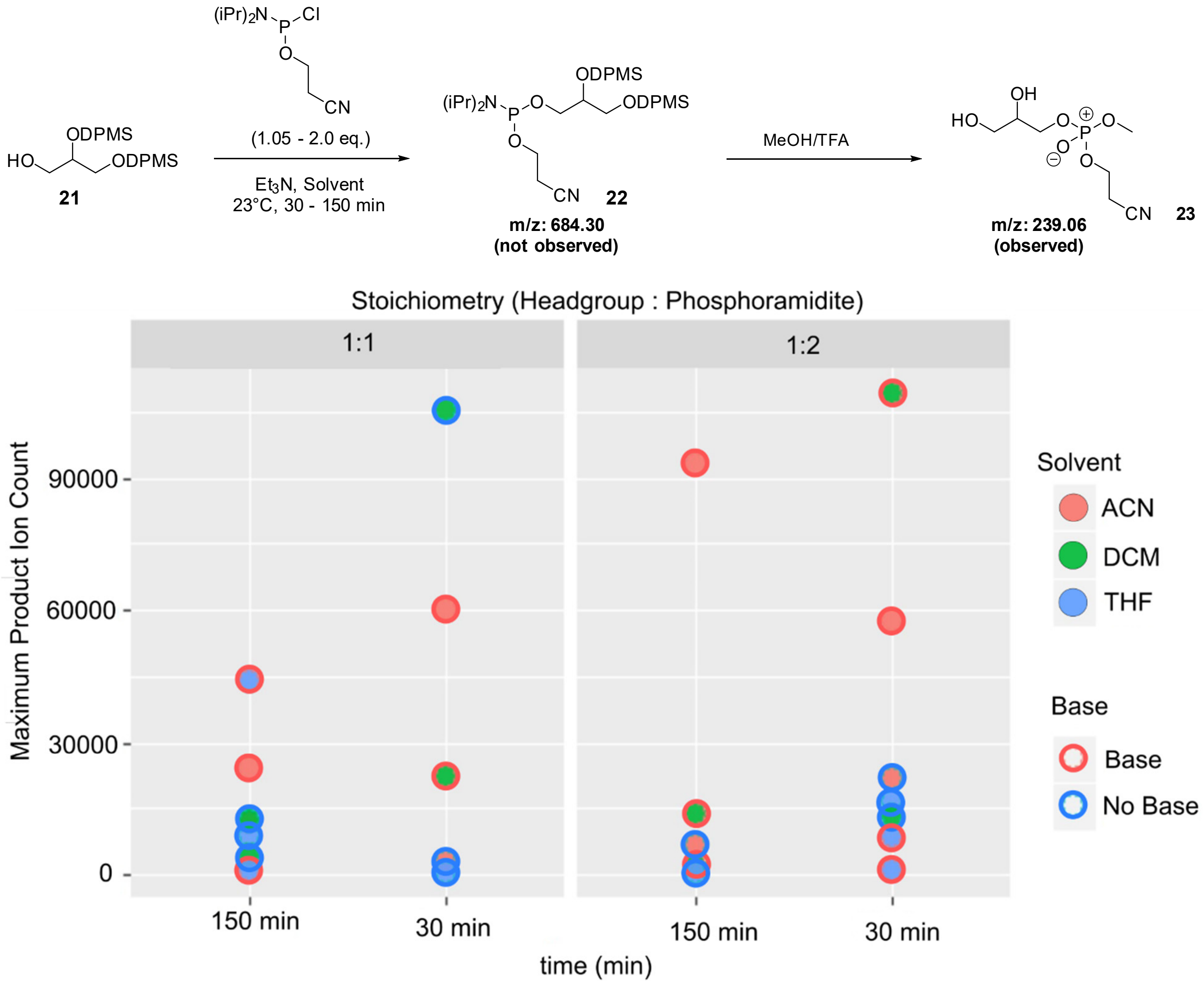


**Figure S5.** HTE of phosphoglycerol headgroup phosphorylation campaign with NCP using DESI-MS analysis.

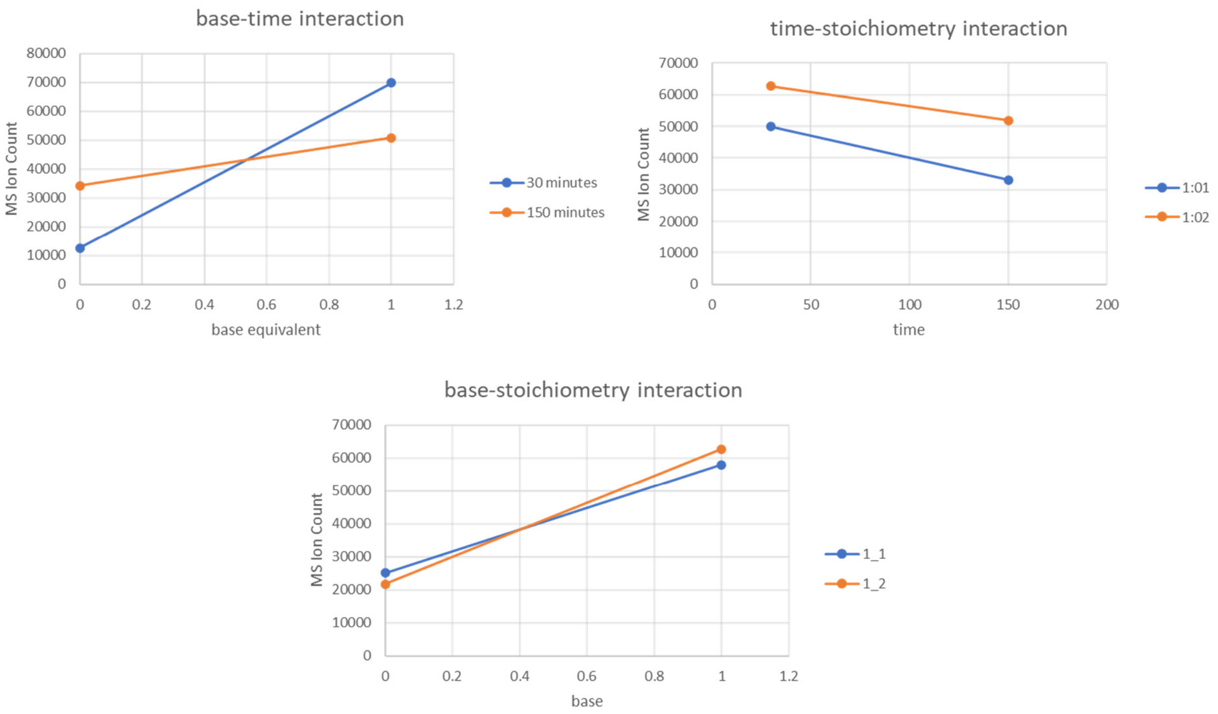


**Figure S6.** Interaction effects plot. Y-axis corresponds to the DESI-MS Ion Count Response, and interaction plot between base-time (top left), time-stoichiometry (top-right), and base-stoichiometry (bottom) is indicated.

The results demonstrated that a two-fold excess of NCP yielded a larger amount of desired product than a 1:1 stoichiometry. However, our HTE system is not currently conducive for air-sensitive reactions such as this. Therefore, it might not be rational to conclude that a two-fold excess of NCP is necessary, especially when precautions are implemented to prevent air and moisture entering the reaction can be taken at the bench. As expected, the presence of a base such as $Et_3N$ appears to be beneficial for the reaction by buffering HCl liberation after substitution, thereby inhibiting DPMS hydrolysis. After optimization of the glycerol headgroup phosphorylation conditions, we translated these results to a flow on a 100-mg scale (Figure S8). For these experiments, we used a 10-μL microchip Chemtrix reactor (Figure S10) containing two staggered-oriented ridge (SOR) mixers prior to entering the reaction zone. Based on the HTE data, it was imperative that the phosphoramidite and the base were homogenized before introducing the protected phosphoglycerol headgroup **21** to avoid hydrolysis of the DPMS ethers. We achieved this by mixing NCP and $Et_3N$ in the first SOR mixer, followed by the introduction of **21** in the second SOR mixer. A residence time of 1 min at 30 °C was optimal for producing phosphoramidite intermediate **22** in 73% yield, compared to a 40% yield for the batch reaction. Longer residence times and lower temperatures resulted in formation of the hydrolysis product and incomplete conversion of the starting material, respectively, as observed by TLC. Additionally, the need for only a slight excess of NCP compared to the two additional equivalents required for batch conditions greatly simplified the purification process, requiring only an aqueous extraction for workup.

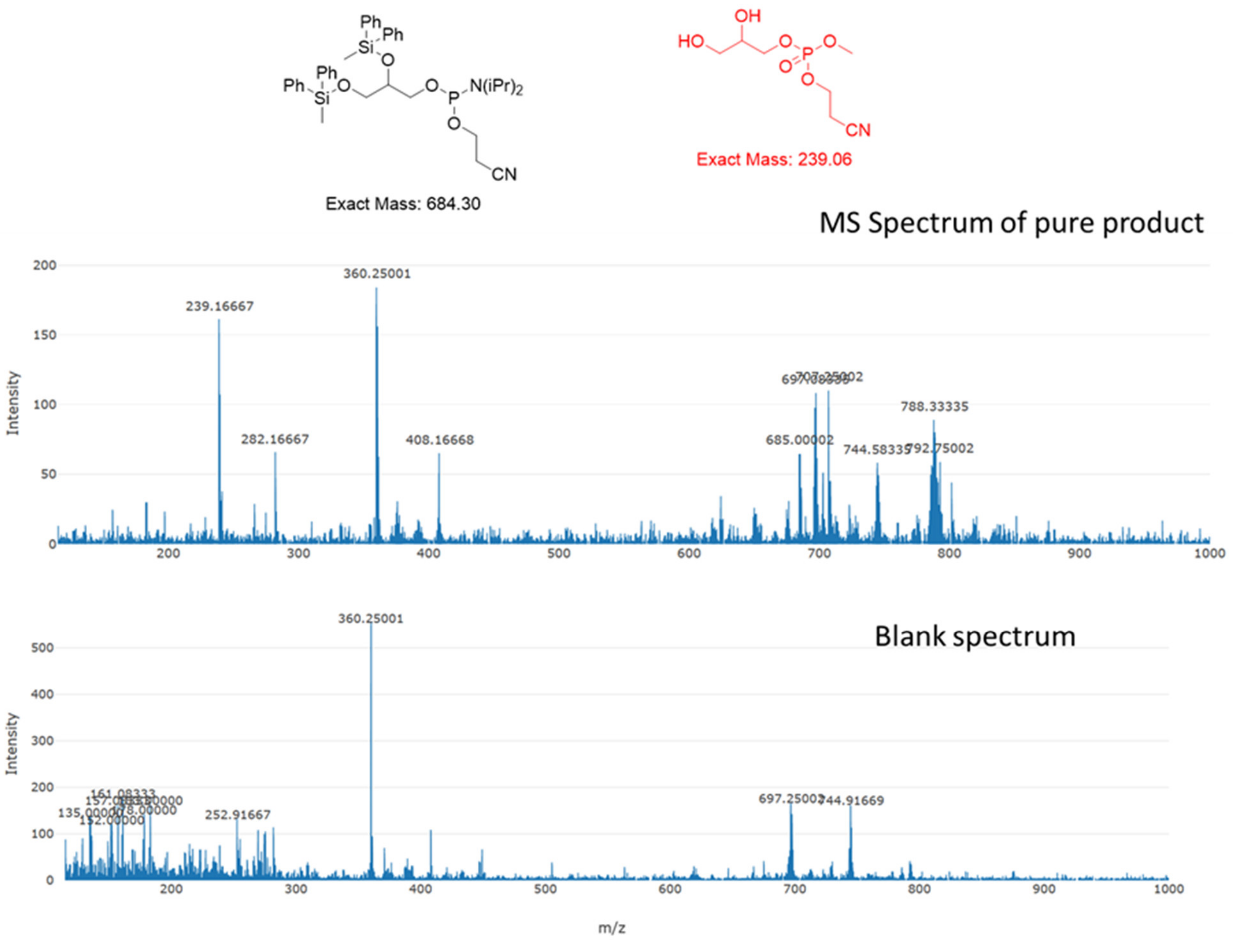


**Figure S7.** DESI-MS spectrum for the authentic product, which has been characterized by NMR (top) and the blank spectrum, where nothing was pinned. The desired product [M+H] is 685, however, the most abundant peak is at m/z 239.1. We attribute the peak at m/z 239.16 to the structure shown in red.

Next, we set out to increase the scale of the reaction from 100 mg to greater than 1 g. The order of addition was conducted similarly to the small-scale experiments, by using a T-mixer followed by a static T-mixer for increased mixing upon the introduction of **21** to induce turbulent flow before entering the temperature-controlled coiled reactor (Figure S12). Flow rates were 0.5 mL/min for each syringe, translating to a total residence time of 1 min. The stoichiometries of NCP and $Et_3N$ were 1.1 and 2, respectively. To our delight, we were able to obtain 1.1 g of our desired product **22** in 75% isolated yield with this reactor configuration.

With phosphoramidite intermediate **22** in hand, we proceeded to phosphorylate the glycerol backbone. Unlike the phosphorylation of **21**, the reaction with the backbone proved to be more challenging, as the substitution of the diisopropylamine substituent is catalyzed by mildly acidic conditions. We returned to small-scale pilot experiments in both flow and batch to optimize these reaction conditions (Table S3). Unfortunately, all tested conditions resulted in significant acyl chain migration of the glycerol backbone, wherein the acyl chain was transesterified to the more thermodynamically stable primary position from the secondary position. No desired product **22** formation was observed by TLC.

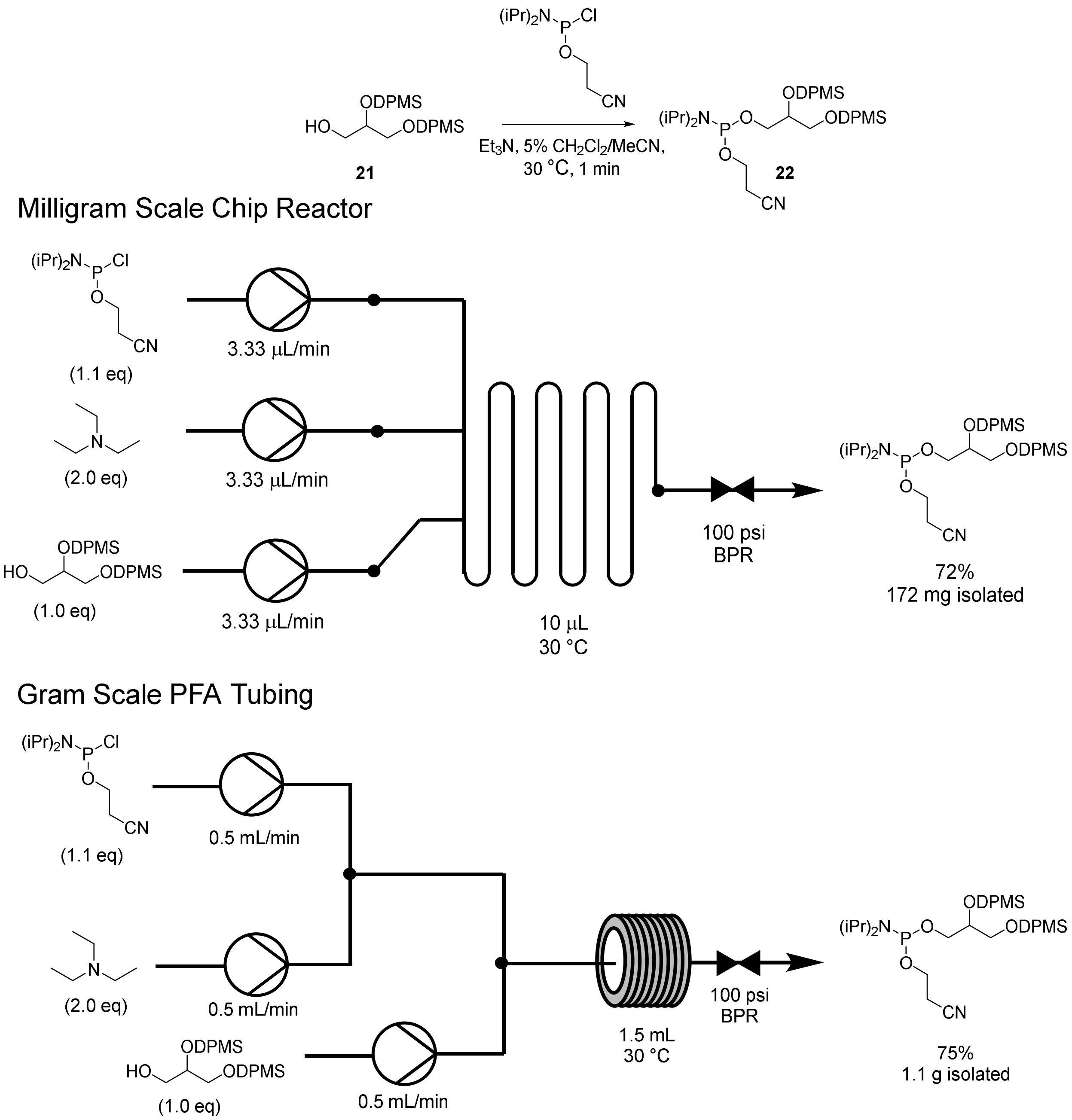


**Figure S8.** Small-scale flow schematic using a microfludidic chip (top). Gram-scale reaction using PFA tubing (bottom).

**Milligram-scale headgroup phosphorylation reactions in flow**

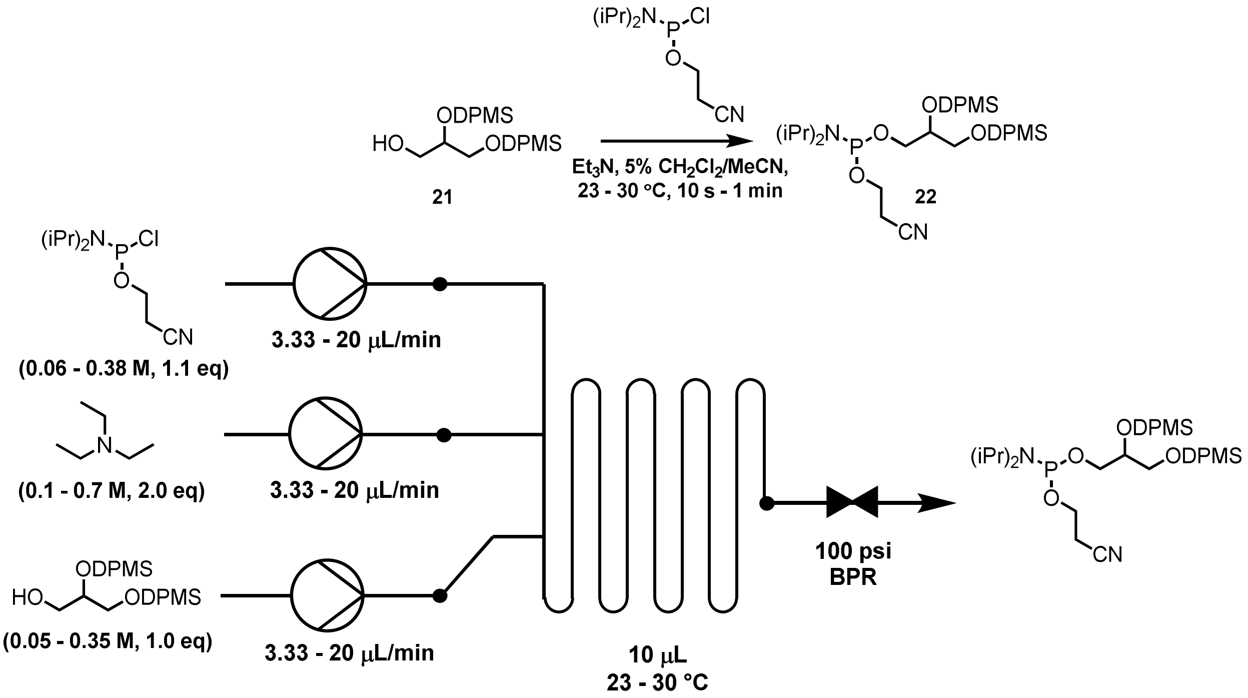


**Figure S9.** Flow schematic of milligram-scale phosphorylation of the phosphoglycerol head group.

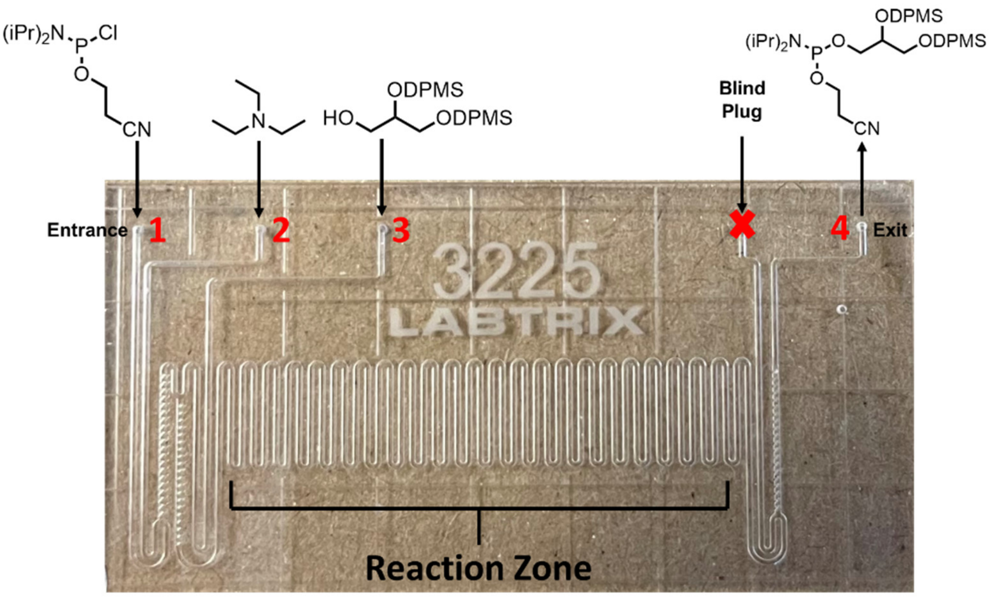


(A)

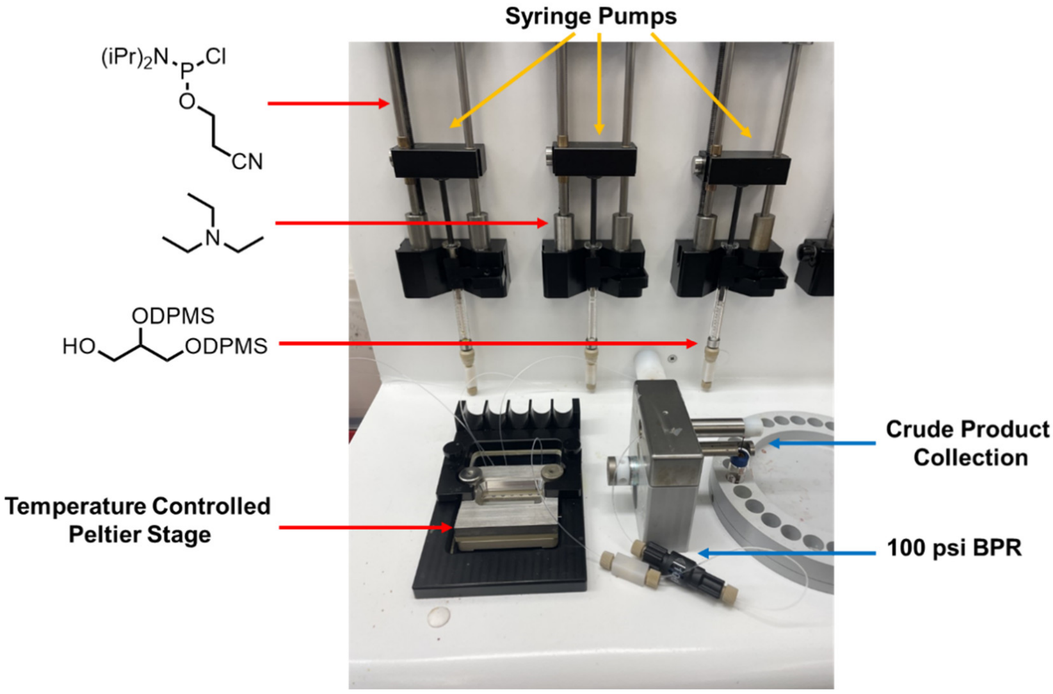


(B)

**Figure S10.** Arrangement of reagents as they enter and exit the microfluidic reactor (**A**). The "reaction zone" comprises the region between the last SOR mixer of the entrance, and the SOR mixer prior to the exit of the chip. Tubing assembly and arrangement of syringes of on the S1 (**B**).

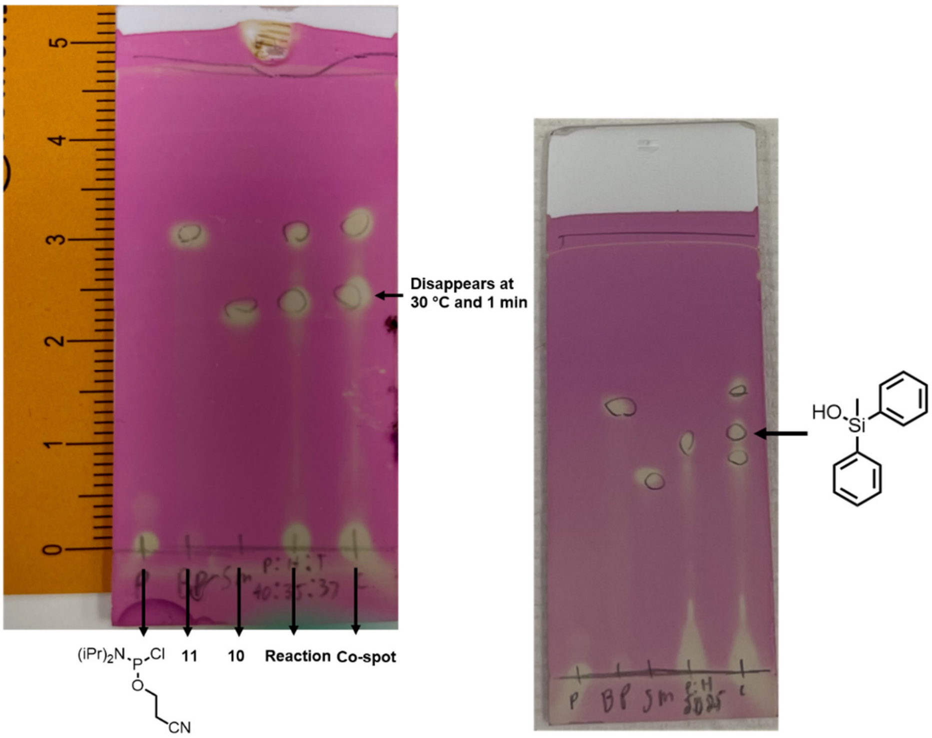


(A) (B)

**Figure S11.** Example of a TLC at a residence time of 30 s and at 23 °C with the addition of $Et_3N$ (**A**). Example of a TLC showing silyl hydrolysis product without the addition of $Et_3N$, most likely due to the formation of HCl (**B**). The order of analytes from left to right on the TLC plate in B is the same as in A.

**Gram scale headgroup phosphorylation reactions in flow**

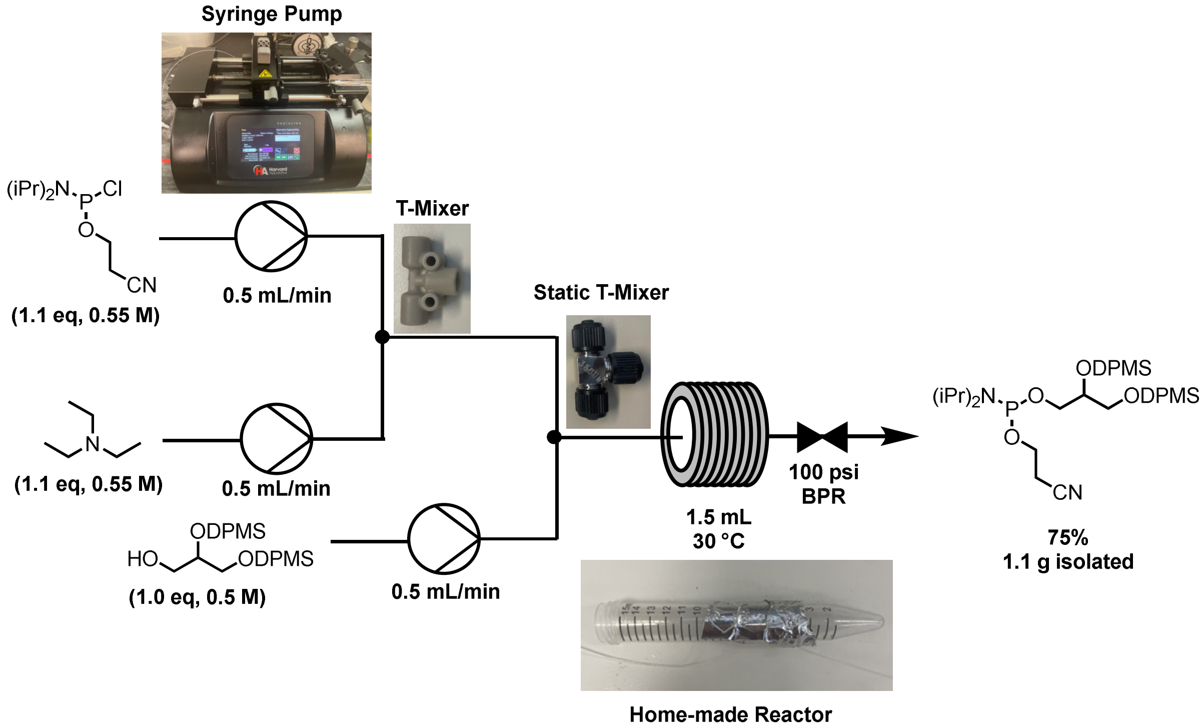


**Figure S12.** Flow schematic of gram-scale reactions containing concentration of reagents and images of components.

**Attempts to phosphorylate the glycerol backbone in a microfluidic chip**

The materials used for the assembly of the flow reactor were identical to the materials used for the milligram-scale headgroup phosphorylation in flow. The flow schematic can be seen in Figure S10. Residence times were calculated using Eq. 1. Three 1-mL, gas-tight syringes were filled with **22**, **8,** and 1*H*-tetrazole solutions made with 5% DCM in dry MeCN. Each syringe was placed on its own syringe pump on the S1. Each solution was 0.5 M in concentration, and equivalencies for each reaction condition were adjusted by modifying the flow rate for each reagent in the Chemtrix S1 software. The mixing of **22** through port 1 and 1*H*-tetrazole of port 2 occurred in the first SOR mixer, followed by the introduction of **8** through port 3 before the second SOR (Figure S14). The temperature of the reaction was controlled by adjusting the heat delivered to the Peltier stage of the S1 using the Chemtrix S1 software. A 100-psi back-pressure regulator was installed after the microfluidic chip and before the 20-mL scintillation vial connected to the flow reactor, which was placed under an Ar atmosphere prior to collecting the product. Products were monitored by TLC after collection. Only two spots appeared, in addition to the starting materials, corresponding to **16** and **24** in an 8:2 Hexane:EtOAc mobile phase.

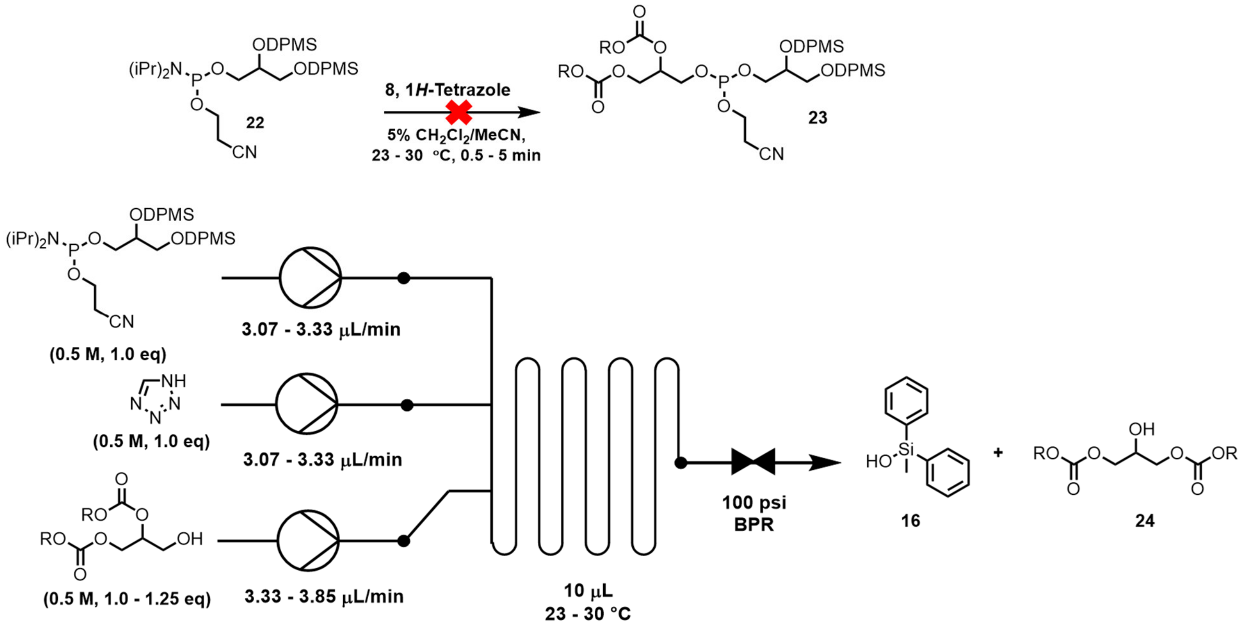


**Figure S13.** Flow schematic of phosphoglycerol backbone phosphorylation.

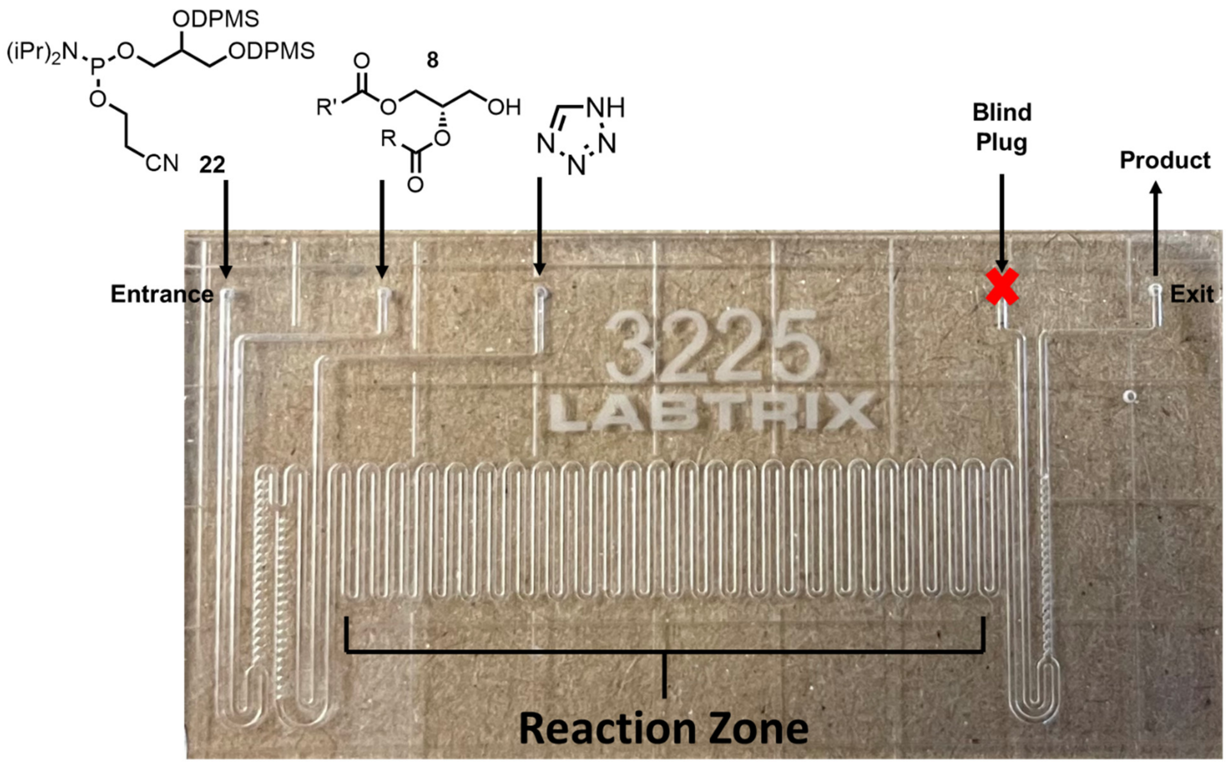


**Figure S14.** Arrangement of reagents as they enter and exit the microfluidic reactor.

## Phosphorylation of Glycerol Backbone

**Table S3.** Attempts to phosphorylate the *sn*-3 phosphoglycerol backbone under acidic conditions. [a]Acyl chain migration observed by TLC.

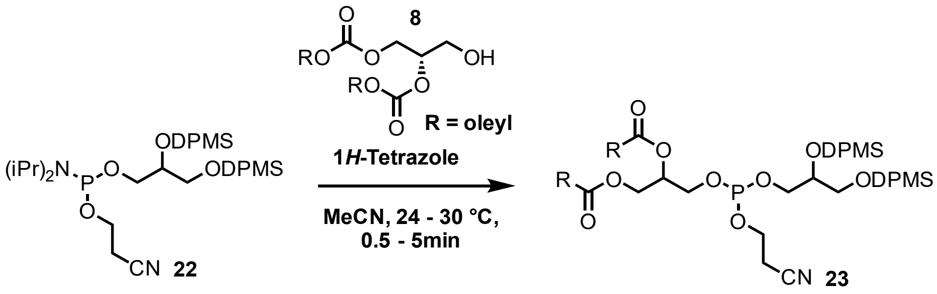


| Entry | Eq. of 12 | Residence Time (min) | Temperature (°C) | Acyl Chain Migration[a] |
|---|---|---|---|---|
| 1 | 1.1 | 0.5 | 23 | No |
| 2 | 1.25 | 0.5 | 30 | No |
| 3 | 1.1 | 1 | 23 | Yes |
| 4 | 1.25 | 1 | 30 | Yes |
| 5 | 1.1 | 5 | 23 | Yes |
| 6 | 1.25 | 5 | 30 | Yes |

**Table S4.** Gradient table for the oxidation and acetonide deprotection of **11.**

| Set of 72 mL collected | Mobile Phase |
|---|---|
| 1 | 98:2 $CHCl_3$:MeOH |
| 2 | 95:5 $CHCl_3$:MeOH |
| 3 | 90:10 $CHCl_3$:MeOH |
| 4 | 85:15 $CHCl_3$:MeOH |
| 5 | 65:25:4 $CHCl_3$:MeOH:$H_2O$ |

## Methods

### High-Throughput Experimentation General Workflow.

The desorption electrospray ionization-mass spectrometry (DESI-MS) experiments were performed using the previously published protocol by Wleklinski et al. [7], along with the Chemical Reaction Screening (CHRIS) module [7], a user-friendly, interactive, home-built software. The reagent stock solutions were prepared and transferred into the reservoirs on the deck of the liquid-handling robot (Biomek, i7, Beckman Coulter) and then transferred using the multi-channel pod and the span-8 pod to prepare the 96-well plates. The 96-well block (Analytical Sales, Inc) was comprised of glass vials inserted into an aluminum block. Once the vials were charged with reagents, the plate was sealed and heated to the desired temperature between using custom-made aluminum 100 W cartridge heaters, which were fabricated in-house (Amy Facility, Purdue University), or placed on the orbital shaker at room temperature. The reaction mixtures were then transferred to 384-well plates and then spotted onto DESI-MS plate without any further purification. The DESI-MS plate was comprised of a porous PTFE sheet (EMDMillipore, Saint Go-bain) mounted onto a glass support (Foxx Life Sciences) via Scotch spray mount (3M). Spotting was carried out using the 50-nL, magnetic, slotted, transfer pin-tool, mounted onto the liquid-handling robot. The data acquisition for DESI-MS was performed using a linear ion trap mass-spectrometer (LTQ, Thermo Scientific), fitted with a commercially available DESI Imaging source (2D Prosolia DESI stage, Waters Corporation). Data analysis was performed using the csv files obtained from the CHRIS-created workflow. CHRIS allows for DESI-MS operation and analysis of the DESI-MS plates by monitoring the ion counts of selected m/z values of interest for the starting material, product, and by-products. The ion counts for the expected m/z values for the product were analyzed by data visualization in R studio, excel and JMP Pro.

### High Throughput Experimentation of Glycerol Head-group Phosphorylation.

We chose a design of experiments (DoE) with 23 full factorial designd for our phosphorylation experiment, with times of 30 minutes and 150 minutes, stoichi-ometries of phorphoramidites at 1:1 and 1:2, and Et3N at 0 and 1 equiv. We prepared 1-mL stock solutions of 0.125 M and 0.25 M 3-((chloro(diisopropylamino)phosphaneyl)oxy)propanenitrile (phosphoramidite) and 0.125 M Et3N in DCM, THF and MeCN. From these stock solutions, respective volumes of each reagent were transferred using the Biomek i7 liquid handling robotic system to individual wells in two different 96-well blocks (Analytical Sales, Inc.) to obtain the final concentration of 0.03M of Headgroup, 0.03 M and 0.06 M (1 equiv. and 2 equiv., respectively) of phosphoramidite and 0.03 M (1 equiv.) of $Et_3N$, with a final solution volume of 150 μL. The 96-well blocks were placed on the orbital shaker for 30 or 150 minutes each. The reaction solution (60 μL) was then transferred to polypropylene 384-well plates. As a control, 60 μL of the pure product (synthesized in flow and characterized by NMR) was manually transferred to one of the wells in a 384-well plate. An aliquot of the solution (50 nL) was then drawn out using the 384 pin-tool from the 384-well plate and then stamped onto a porous PTFE surface glued to glass (DESI-slide), with 4 replicates. DESI-MS was performed using MeOH + 0.1% formic acid as the spray solvent, and the average product ion count was generated using home-built CHRIS software, which was coupled to the DESI-MS readout. The average product ion intensities were used to perform further analysis using R programming and Excel.

### Milligram Scale Synthesis of 2,3-bis((methyldiphenylsilyl)oxy)propyl(2-cyano-ethyl)diisopropylphosphoramidite (22).

A flow schematic can be seen in Figure S7. Reactions for the translation of HTE data to small-scale pilot flow experiments were carried out using three 1-mL, gas-tight syringes on a Chemtrix Labtrix S1 system (S1), which were connected to a Chemtrix Labtrix 3225

glass microfluidic reactor chip (Figure S8). Each syringe was filled with reagent solution and attached to its own individual pump on the S1. The solutions were all made in 5% DCM in dry MeCN, where the phosphoramidite concentration was 0.06 – 0.38 M, $Et_3N$ concentration was 0.1 – 0.7 M, and alcohol **10** was 0.05 – 0.35 M. SOR mixers in these chips served to induce turbulent flow in the reagents to ensure adequate mixing. PFA tubing was used to connect the microfluidic chip to the syringes and collection vial. The phosphoramidite and the $Et_3N$ solutions were mixed upon entering the chip through ports 1 and 2. After passage through the first SOR mixer, 10 was introduced into port 3 prior to the second SOR mixer and the reaction zone. The residence time ($T_r$), expressed in min, can be calculated using the following equation:

$$T_r = \frac{V_r}{V_f} \quad (1)$$

where $V_r$ is the total volume of the reactor in mL, and $V_f$ is the volumetric flow rate in μL/min. Residence times and temperature were controlled by adjusting the flow rates and heat delivered to the Peltier stage, respectively, by using the Chemtrix Labtrix software. Residence times were evaluated between 10 s and 1 min, and evaluated by TLC. The temperature varied between 23 and 30 °C, as required by the experiment. Samples were collected in 20-mL glass scintillation vials capped with a rubber septum. The vials were purged and maintained under an Ar atmosphere via an Ar ballon during collection. Complete disappearance of the starting material by TLC did not occur until the concentrations of each reactant were raised 7-fold from the initial concentrations, at a residence time of 1 min at 30 °C (Figure S9B). The tubing used before and after the glass reactor chip consisted of FEP (0.8 mm OD x 0.25 mm ID). The 100-psi back-pressure regulator was installed after the crude product exited the microfluidic chip. Check valves were attached to the male ends of each syringe to prevent backflow. After the reaction conditions were optimized, a prep-scale run (172 mg) was conducted by continuously collecting crude product solution until all three syringes were depleted. The crude reaction mixture was then diluted with 30 mL of DCM and washed with saturated $NaHCO_3$ (3 x 10 mL). The combined organic extracts were then dried with anhydrous $Na_2SO_4$ and concentrated in vacuo. The crude oil was purified using a Biotage flash purification system (hexane:EtOAc). The product was isolated as a clear oil with 75% yield (refer to Table S7 for gradient); $R_f$=0.27 (DCM:MeOH, 98:2); $^1$H NMR (500 MHz, $CDCl_3$) δ 7.66 – 7.47 (m, 8H), 7.45 – 7.28 (m, 12H), 4.01 (h, *J* = 5.3 Hz, 1H), 3.82 – 3.45 (m, 8H), 2.53 – 2.33 (m, 2H), 1.16 (dd, *J* = 6.8, 2.6 Hz, 6H), 1.08 (d, *J* = 6.8 Hz, 6H), 0.64 (s, 3H), 0.57 (d, *J* = 4.7 Hz, 3H). $^{13}$C NMR (126 MHz, $CDCl_3$) δ 135.81, 134.53, 134.49, 134.42, 129.82, 129.75, 129.72, 127.84, 127.76, 117.67, 117.62, 77.30, 77.05, 76.79, 73.60, 73.54, 73.48, 73.41, 64.84, 64.74, 64.58, 64.45, 64.18, 64.06, 58.61, 58.46, 58.41, 58.26, 43.11, 43.08, 43.01, 42.98, 24.66, 24.59, 20.28, 20.23, -2.22, -2.32, -3.18. $^{31}$P NMR (202 MHz, $CDCl_3$) δ 148.38, 148.08. QTOF-HRMS (ESI) for $C_{35}H_{49}N_2O_4PSi_2$ [M+Na$^+$]: found 685.3048, calcd 685.3041.

**Gram-Scale Synthesis of 2,3-bis((methyldiphenylsilyl)oxy)propyl(2-cyanoethyl)diisopropylphosphoramidite (22).**

Reactions of **22** on the gram scale were carried out using three 5-mL, gas-tight syringes attached to two Hamilton PHD Ultra syringe pumps. The phosphoramidite and $Et_3N$ solutions were placed in one pump (P1), and the syringe containing the alcohol solution was placed in the other pump (P2). All reagent solutions were created with 5% DCM in dry MeCN. It should be noted that the alcohol solution was slightly turbid. P1 operated at a flow-rate of 0.25 mL/min, and P2 operated at 0.5 mL/min, so that the total flowrate was 1.5 mL/min. PFA tubing was used for these flow experiments (1/16″ OD x 0.03″ ID). The phosphoramidite and $Et_3N$ were mixed in a standard T-mixer, followed by mixing with the alcohol in a static T-mixer (Figure S10). Once all three reagents were mixed, they entered the home-made reactor consisting of tubing coiled around a 15-mL Falcon Tube and held in place with aluminum tape. The total volume of the reactor was

1.5 mL. Residence times were calculated using Eq. 1. This assembly was subsequently placed in a temperature-controlled oil bath, where the temperature was set to 30 °C for the duration of the reaction. At the end of the tubing was a Luer lock assembly, where a needle was attached so that the crude reaction mixture emptied into a 20-mL scintillation vial, placed under a septum-sealed Ar atmosphere. A 100-psi back-pressure regulator was placed at the end of the reaction line. The reaction mixture was then diluted with DCM and washed with saturated $NaHCO_3$ (3 x 30 mL). The washed organic layer was dried with anhydrous $Na_2SO_4$ and concentrated under reduced pressure. The crude product was purified on a Biotage instrument flash purification system (hexane:EtOAc) (refer to Table S6 for gradient). The product was isolated as a clear oil with 75% yield; $R_f$=0.27 (DCM:MeOH, 98:2); $^1$H NMR (500 MHz, $CDCl_3$) δ 7.66 – 7.47 (m, 8H), 7.45 – 7.28 (m, 12H), 4.01 (h, *J* = 5.3 Hz, 1H), 3.82 – 3.45 (m, 8H), 2.53 – 2.33 (m, 2H), 1.16 (dd, *J* = 6.8, 2.6 Hz, 6H), 1.08 (d, *J* = 6.8 Hz, 6H), 0.64 (s, 3H), 0.57 (d, *J* = 4.7 Hz, 3H). $^{13}$C NMR (126 MHz, $CDCl_3$) δ 135.81, 134.53, 134.49, 134.42, 129.82, 129.75, 129.72, 127.84, 127.76, 117.67, 117.62, 77.30, 77.05, 76.79, 73.60, 73.54, 73.48, 73.41, 64.84, 64.74, 64.58, 64.45, 64.18, 64.06, 58.61, 58.46, 58.41, 58.26, 43.11, 43.08, 43.01, 42.98, 24.66, 24.59, 20.28, 20.23, -2.22, -2.32, -3.18. $^{31}$P NMR (202 MHz, $CDCl_3$) δ 148.38, 148.08. QTOF-HRMS (ESI) for $C_{35}H_{49}N_2O_4PSi_2$ [M+Na$^+$]: found 685.3048, calcd 685.3041.

**Synthesis of (2,2-dimethyl-1,3-dioxolan-4-yl)methyl 2-phenylacetate (18).**

Solketal (9.3 mL, 75 mmol) was placed in an oven-dried, 250-mL, multi-neck, round-bottom flask, equipped with a magnetic stir bar. The flask was dried via Schlenk techniques, and an Ar balloon was attached. Dry DCM (55 mL) and $Et_3N$ (12 mL, 86 mmol) was added to the flask, and the mixture was allowed to cool to 0 °C in an ice bath. Phenylacetyl chloride (11 mL, 83 mmol) was then added dropwise, allowed to stir for 20 min at 0 °C, and warmed up to 24 °C, where it continued to react for another 1.5 h. The crude product was then poured over 60-mL DI $H_2O$ in a 250-mL separatory funnel. The organic layer was extracted, and the aqueous layer was washed with DCM (3 x 30 mL). The combined organic extracts were dried with anhydrous $Na_2SO_4$ and concentrated under reduced pressure. The crude product was purified on a silica gel column (gradient of 9:1 to 8:2 Hexane:EtOAc) to yield the product as a clear, yellow oil with 88% yield; $R_f$=0.42 (Hexane:EtOAc, 8:2); $^1$H NMR (500 MHz, $CDCl_3$) δ 7.38 – 7.23 (m, 5H), 4.30 (qd, *J* = 6.1, 4.7 Hz, 1H), 4.15 (qd, *J* = 11.5, 5.3 Hz, 2H), 4.03 (dd, *J* = 8.5, 6.5 Hz, 1H), 3.69 (dd, *J* = 8.5, 6.2 Hz, 1H), 3.67 (s, 2H), 1.41 (s, 3H), 1.36 (s, 3H). $^{13}$C NMR (126 MHz, $CDCl_3$) δ 171.36, 133.76, 129.29, 128.96, 128.62, 127.38, 127.21, 109.82, 77.36, 77.11, 76.85, 73.54, 66.26, 64.96, 41.14, 26.66, 25.43. QTOF-HRMS (ESI) for $C_{14}H_{18}O_4$ [M+Na$^+$]: found 273.1099, calcd 273.1097.

**Synthesis of 2,3-dihydroxypropyl 2-phenylacetate (19).**

Compound **7** (3.0 g, 12 mmol) was placed in a 250-mL round-bottom, equipped with a magnetic stir bar, and dissolved in THF (33 mL). A 1M HCl solution (33 mL) was then added to the flask, and the reaction was stirred for 3 h at 24 °C. A saturated solution of $NaHCO_3$ was then slowly added, until $CO_2$ evolution ceased. The reaction mixture was then washed with EtOAc (3 x 150 mL) and dried with anhydrous $Na_2SO_4$ before being concentrated under reduced pressure. The crude product was then purified on a silica gel column (gradient of 98:2 to 96:4 DCM:MeOH) to yield the product as a white solid, with 94% yield; $R_f$=0.92 ($CHCl_3$:MeOH:$H_2O$, 65:25:4); $^1$H NMR (500 MHz, $CDCl_3$) δ 7.35 – 7.22 (m, 5H), 4.12 (dd, *J* = 5.5, 1.2 Hz, 2H), 3.85 (qd, *J* = 5.7, 3.7 Hz, 1H), 3.64 (s, 2H), 3.58 (dd, *J* = 11.6, 3.7 Hz, 1H), 3.48 (dd, *J* = 11.6, 6.1 Hz, 1H), 3.34 (s, 2H). $^{13}$C NMR (126 MHz, $CDCl_3$) δ 172.14, 172.05, 133.77, 133.68, 129.28, 129.25, 128.82, 128.69, 127.30, 77.40, 77.14, 76.89, 70.07, 65.58, 63.33, 61.59, 41.25, 41.15. QTOF-HRMS (ESI) for $C_{11}H_{14}O_4$ [M+Na$^+$]: found 233.0786, calcd 233.0784.

**Synthesis of 2,3-bis((methyldiphenylsilyl)oxy)propyl 2-phenylacetate (20).**

**8** (1.0 g, 4 mmol) and imidazole (0.95 g, 14 mmol) was placed in a 50-mL, oven-dried, multi-neck, round-bottom flask, equipped with a magnetic stir bar. The flask was cycled 3 times with vacuum/Ar, and an Ar balloon was attached to the round-bottom flask. DMF (10 mL) was added, and the mixture was stirred until everything was dissolved. The reaction was subsequently cooled to 0 °C in an ice bath, where DPMSCl (2.9 mL, 14 mmol) followed by $Et_3N$ (1.3 mL, 10 mmol) was added. The reaction was stirred in the ice bath for 1.5 h, until it was moved to a 50 °C oil bath and allowed to react for another 2.5 h. The reaction was then quenched with 40 mL of DI $H_2O$ and extracted with EtOAc (3 x 40 mL). The combined organic layers were then washed with $H_2O$, then brine. The organic layer was dried with anhydrous $Na_2SO_4$ and concentrated under reduced pressure. The crude product was then purified on a silica gel column (hexane:EtOAc) to yield the product as a turbid colorless oil, with 89% yield (refer to Table S5 for gradient); $R_f$=0.55 (Hexane:EtOAc, 8:2); $^1$H NMR (500 MHz, $CDCl_3$) δ 7.59 – 7.12 (m, 27H), 4.28 (dd, *J* = 11.3, 3.6 Hz, 1H), 4.12 (dd, *J* = 11.2, 6.3 Hz, 1H), 4.06 (qd, *J* = 6.0, 3.5 Hz, 1H), 3.69 – 3.62 (m, 2H), 3.45 (s, 2H), 0.58 (d, *J* = 23.9 Hz, 7H); $^{13}$C NMR (126 MHz, $CDCl_3$) δ 171.41, 136.17, 136.09, 135.54, 135.51, 134.44, 134.40, 134.37, 133.91, 129.93, 129.85, 129.30, 128.54, 127.91, 127.84, 127.05, 77.30, 77.04, 76.79, 71.54, 66.14, 64.41, 41.08, 31.62, 22.69, 14.16, -2.50, -3.21, -3.29. QTOF-HRMS (ESI) for $C_{37}H_{38}O_4Si_2$ [M+Na$^+$]: found 625.2203, calcd 625.2200.

**Synthesis of 2,3-Bis((methyldiphenylsilyl)oxy)propan-1-ol (21).**

9 (0/85 g, 1.5 mmol) was placed in an oven-dried, multi-neck, round-bottom flask equipped with a magnetic stir bar. The flask was dried out using Schlenk techniques and placed under an Ar atmosphere via an Ar balloon attached to the flask. Dry toluene (10 mL) was then added to the flask, and the solution was subsequently cooled to -78 °C with a dry ice/acetone bath. After 10 min of cooling, DIBAL-H (1M in hexane, 3.1 mL, 3.1 mmol) was added dropwise to the flask. The reaction was allowed to proceed at -78 °C for 1.5 h. A saturated solution of ammonium chloride (5 mL) was then added to the flask, and the reaction was slowly warmed to 23 °C. The solution was then diluted with 20 mL of EtOAc and 20 mL of DI $H_2O$. The heterogeneous solution was then filtered through a celite bed over a coarse glass frit to remove the alumina produced from the work-up. The organic layer was then collected, and the aqueous layer was washed with EtOAc (3 x 20 mL). The combined organic extracts were dried with anhydrous $Na_2SO_4$ and concentrated in vacuo. The crude product was then purified on a silica gel column and the product was isolated as a clear oil, with 79% yield (refer to Table S6 for gradient); $R_f$=0.44 (Hexane:EtOAc, 8:2); $^1$H NMR (500 MHz, $CDCl_3$) δ 7.61 – 7.50 (m, 8H), 7.42 (tdt, *J* = 5.9, 3.0, 1.4 Hz, 4H), 7.39 – 7.31 (m, 8H), 4.14 (q, *J* = 7.2 Hz, 1H), 3.96 (qd, *J* = 5.9, 2.8 Hz, 1H), 3.76 – 3.62 (m, 4H), 2.06 (s, 1H), 1.28 (t, *J* = 7.1 Hz, 1H), 0.64 (d, *J* = 1.2 Hz, 3H), 0.58 (d, *J* = 1.2 Hz, 3H). $^{13}$C NMR (126 MHz, $CDCl_3$) δ 135.99, 135.96, 135.52, 134.36, 133.99, 130.03, 129.96, 128.00, 127.93, 77.32, 77.07, 76.81, 73.53, 64.59, 64.50, 60.44, 21.09, 14.24, -2.55, -3.30. QTOF-HRMS (ESI) for $C_{29}H_{32}O_3Si_2$ [M+Na$^+$]: found 507.1781, calcd 507.1782.

## Gradient Tables Used for FPLC

**Table S5.** Purification of **20.**

| Flow Rate | Apolar:Polar Solvent | Gradient Steps (in % EtOAc) | | |
|---|---|---|---|---|
| | | Start (%) | End (%) | Length (mL) |
| 17 mL/min | Hexane:EtOAc | 0 | 0 | 72 |
| | | 0 | 1 | 72 |
| | | 1 | 1 | 72 |
| | | 1 | 2 | 72 |
| | | 2 | 2 | 72 |
| | | 2 | 3 | 72 |
| | | 3 | 3 | 72 |
| | | 3 | 4 | 72 |
| | | 4 | 4 | 72 |
| | | 4 | 5 | 72 |
| | | 5 | 5 | 63 |
| | | 5 | 10 | 9 |
| | | 10 | 10 | 72 |

**Table S6.** Purification of **21.**

| Flow Rate | Apolar:Polar Solvent | Gradient Steps (in % EtOAc) | | |
|---|---|---|---|---|
| | | Start (%) | End (%) | Length (mL) |
| 17 mL/min | Hexane:EtOAc | 0 | 1 | 72 |
| | | 1 | 1 | 72 |
| | | 1 | 2 | 72 |
| | | 2 | 2 | 144 |
| | | 2 | 3 | 72 |
| | | 3 | 3 | 144 |
| | | 3 | 4 | 72 |
| | | 4 | 4 | 144 |
| | | 4 | 5 | 72 |
| | | 5 | 5 | 72 |
| | | 5 | 6 | 72 |
| | | 6 | 6 | 144 |

**Table S7.** Purification of **22.**

| Flow Rate | Apolar:Polar Solvent | Gradient Steps (in % EtOAc) | | |
|---|---|---|---|---|
| | | Start (%) | End (%) | Length (mL) |
| 17 mL/min | Hexane:EtOAc | 5 | 5 | 90 (equilibration) |
| | | 5 | 5 | 27 |
| | | 30 | 30 | 78 |

**Table S8.** Purification of **5.**

| Flow Rate | Apolar:Polar Solvent | Gradient Steps (in % MeOH) | | |
|---|---|---|---|---|
| | | Start (%) | End (%) | Length (mL) |
| **17 mL/min** | **DCM:MeOH** | 0 | 0 | 72 |
| | | 0 | 1 | 72 |
| | | 1 | 1 | 72 |
| | | 1 | 8 | 72 |
| | | 8 | 8 | 288 |

**Table S9.** Purification of **6.**

| Flow Rate | Apolar:Polar Solvent | Gradient Steps (in % EtOAc) | | | |
|---|---|---|---|---|---|
| | | Start (%) | End (%) | Length (mL) | |
| **15 mL/min** | **Hexane:EtOAc** | 0 | 5 | 576 | Note: First 72 mL of this gradient step are initial waste |
| | | 5 | 5 | 72 | |
| | | 5 | 7 | 360 | |
| | | 7 | 20 | 9 | |
| | | 20 | 20 | 135 | |

(Note: This gradient is run a second time on the same product, in order to get full separation.).

**Table S10.** Purification of **7.**

| Flow Rate | Apolar:Polar Solvent | Gradient Steps (in % EtOAc) | | |
|---|---|---|---|---|
| | | Start (%) | End (%) | Length (mL) |
| **17 mL/min** | **Hexane:EtOAc** | 0 | 1 | 144 |
| | | 1 | 2 | 144 |
| | | 2 | 3 | 144 |
| | | 3 | 4 | 144 |
| | | 4 | 6 | 144 |
| | | 6 | 8 | 144 |

(Note: Used CHROMAFIL Xtra CA-45/25 filters while loading sample).

**Table S11.** Purification of **8.**

| Flow Rate | Apolar:Polar Solvent | Gradient Steps (in % EtOAc) | | |
|---|---|---|---|---|
| | | Start (%) | End (%) | Length (mL) |
| **15 mL/min** | **Hexane:EtOAc** | 0 | 0 | 72 |
| | | 0 | 1 | 72 |
| | | 1 | 2 | 72 |
| | | 2 | 3 | 72 |
| | | 3 | 4 | 72 |
| | | 4 | 5 | 72 |
| | | 5 | 6 | 48 |

## NMR of All Compounds

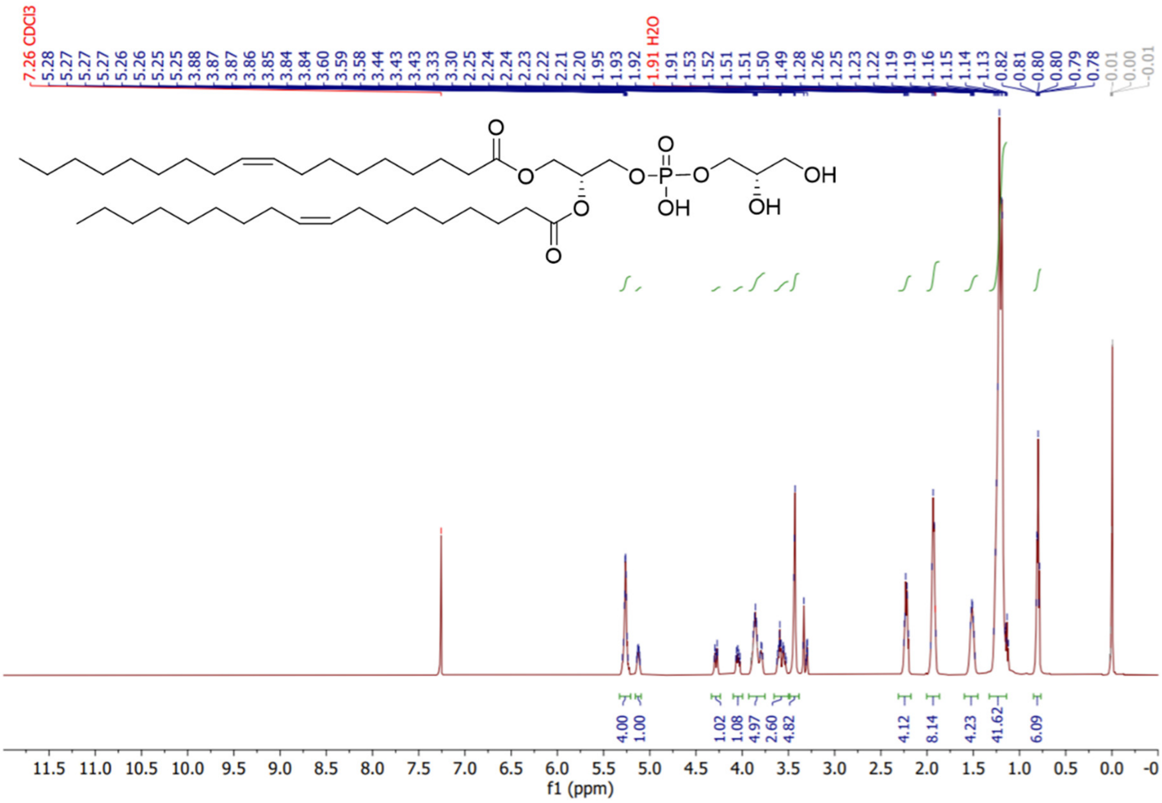


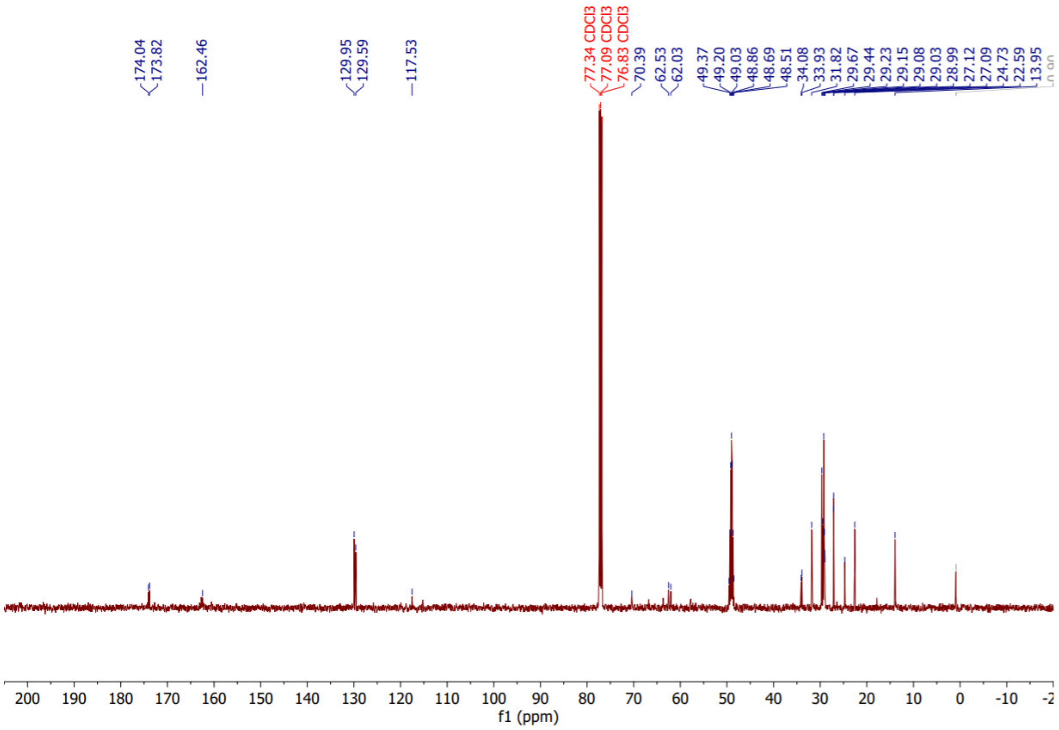

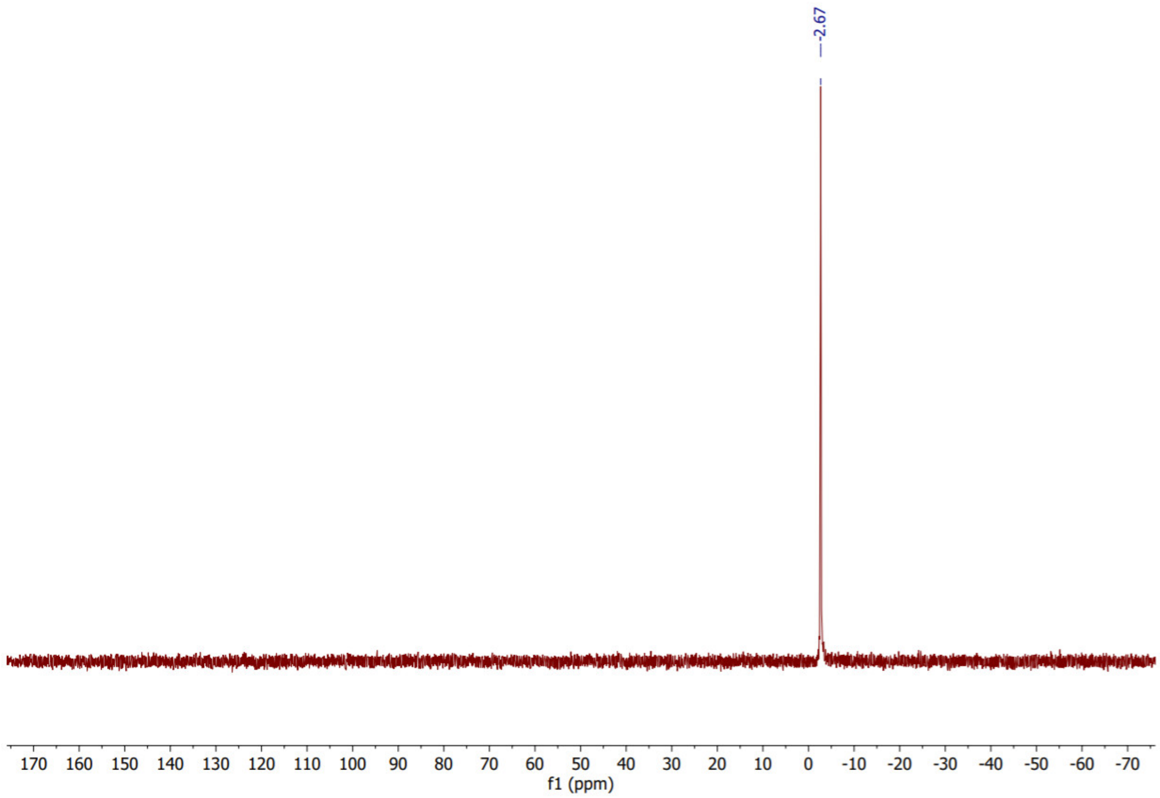


**Figure S15.** $^{1}$H, $^{13}$C, and $^{31}$P NMR of **1.**

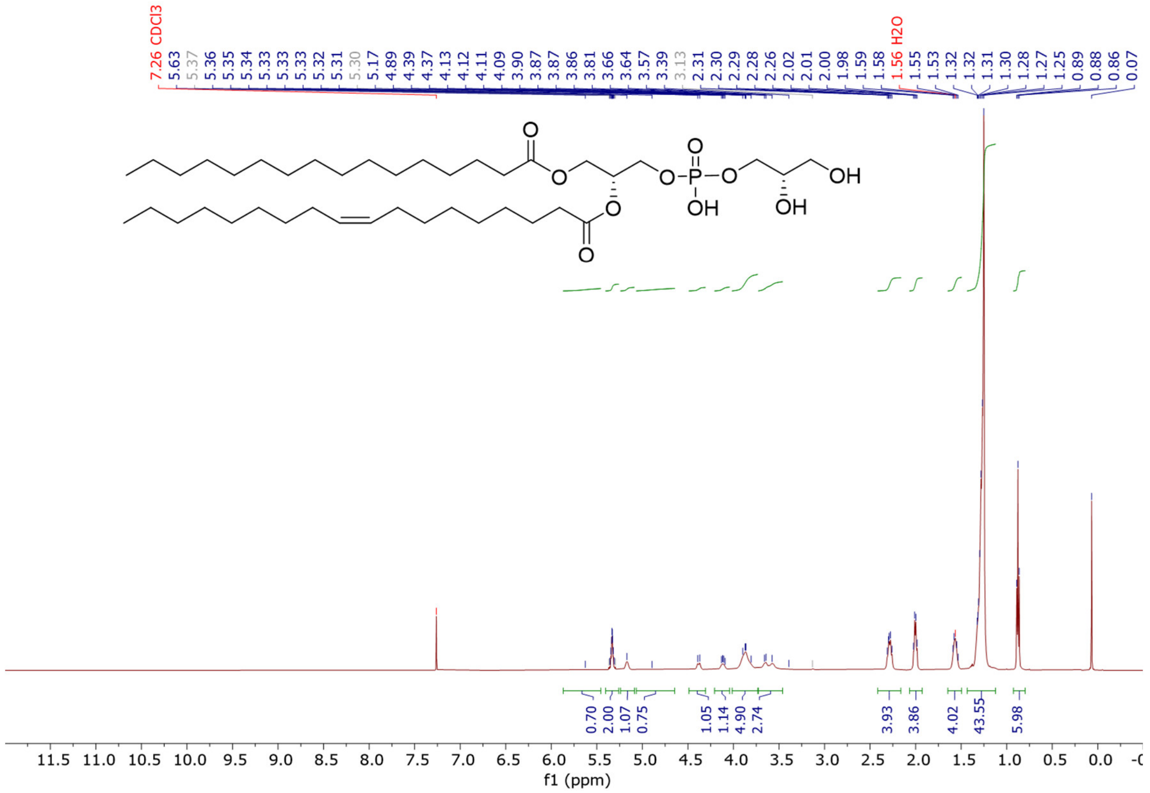

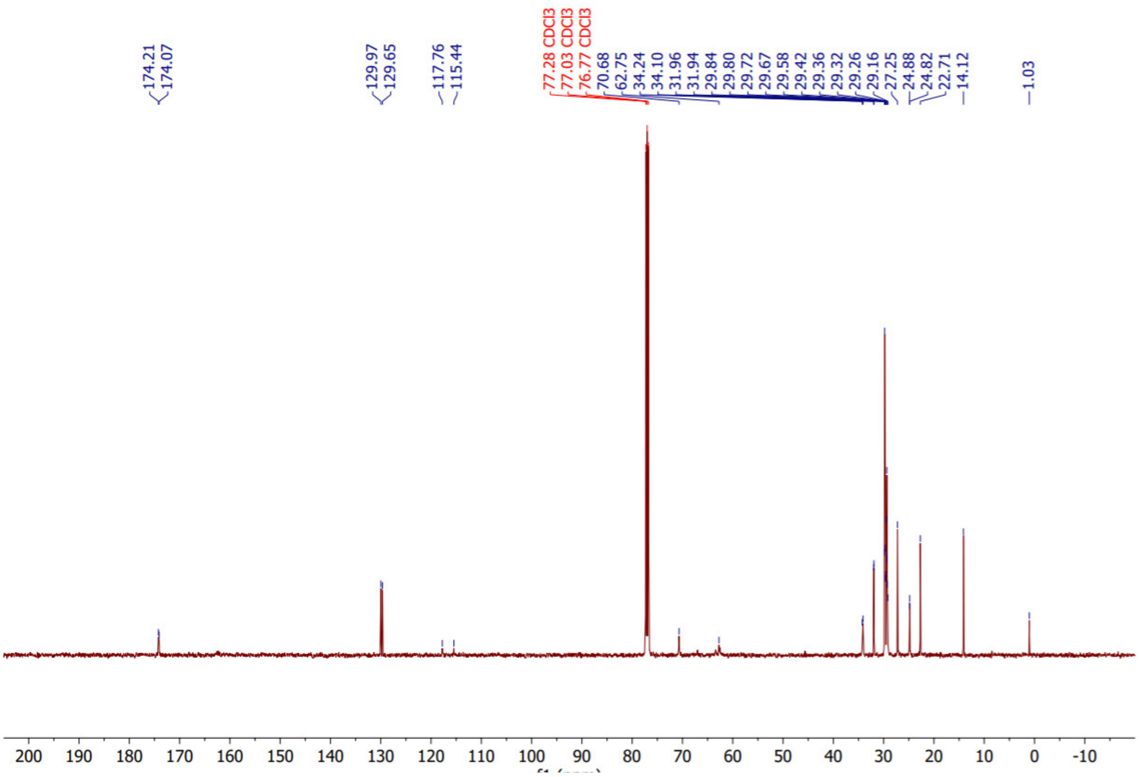


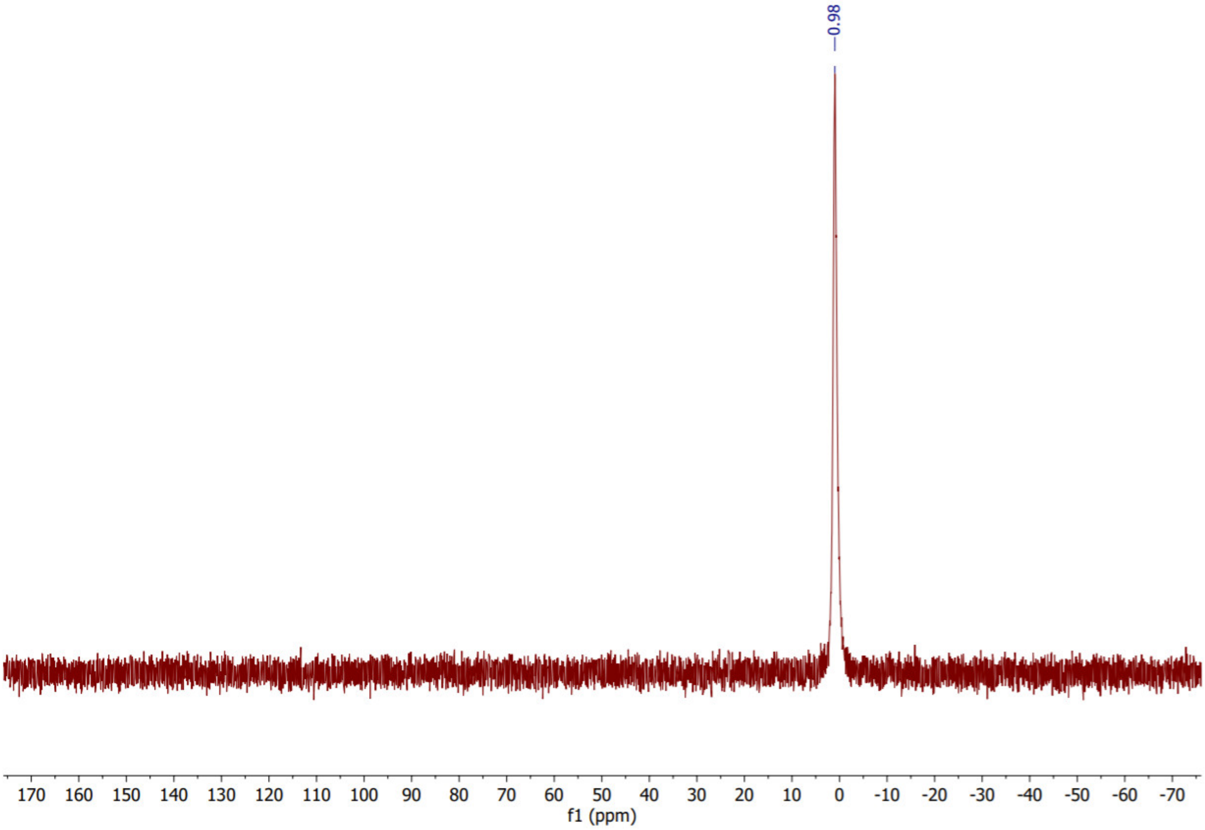


**Figure S16.** $^{1}H$, $^{13}C$, and $^{31}P$ NMR of **2.**

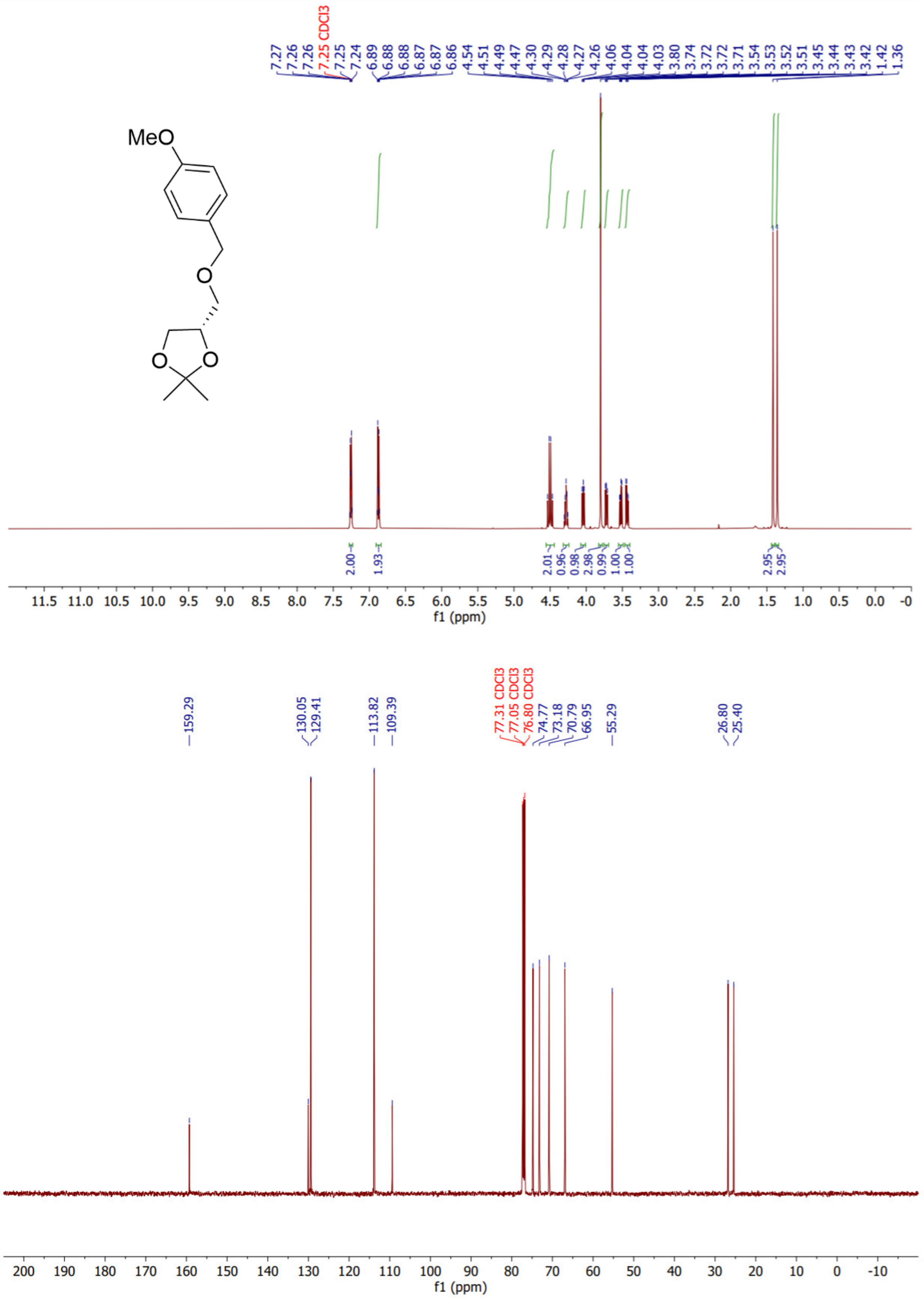


**Figure S17.** $^{1}H$ and $^{13}C$ NMR of **4.**

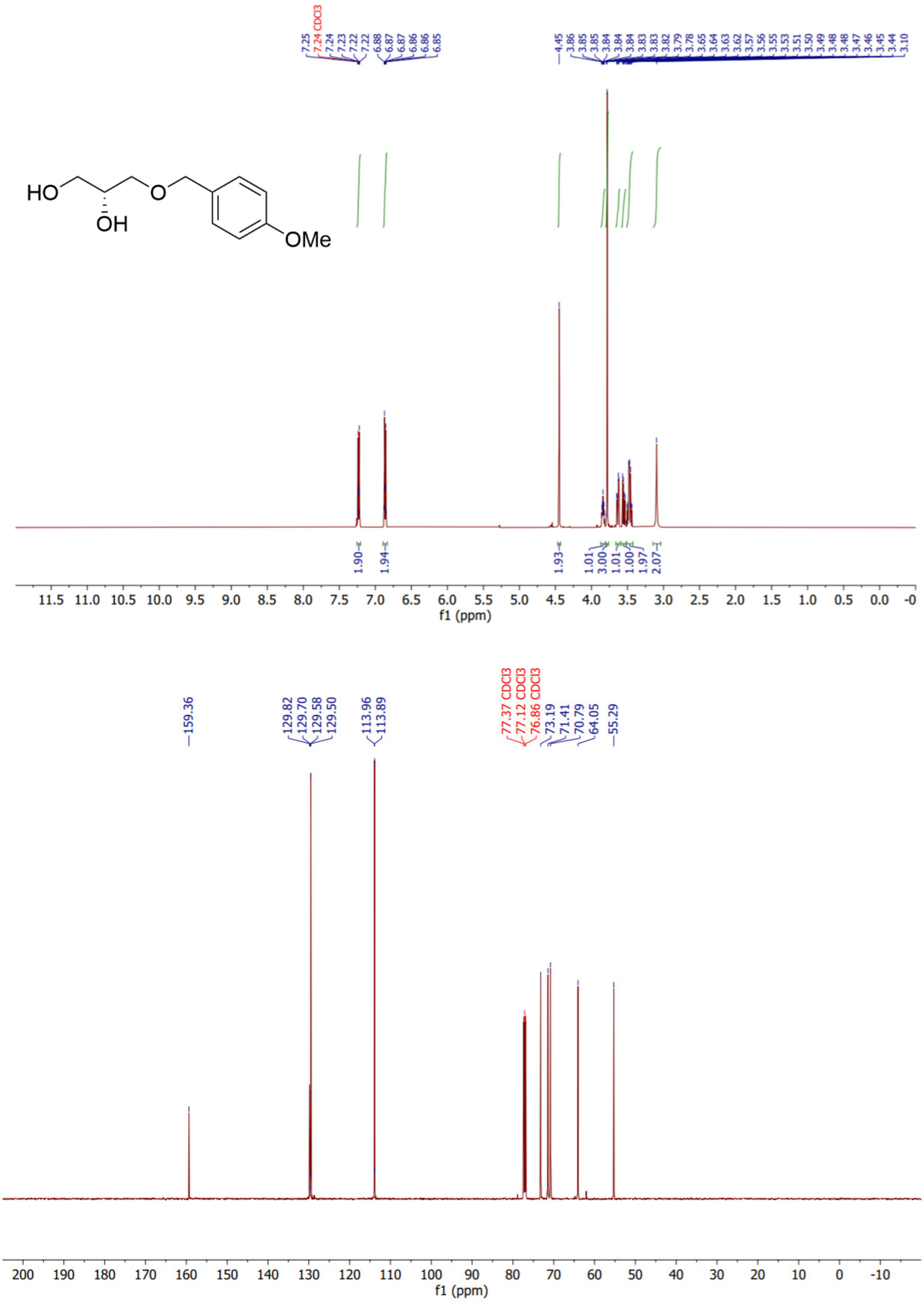


**Figure S18.** $^{1}H$ and $^{13}C$ NMR of **5.**

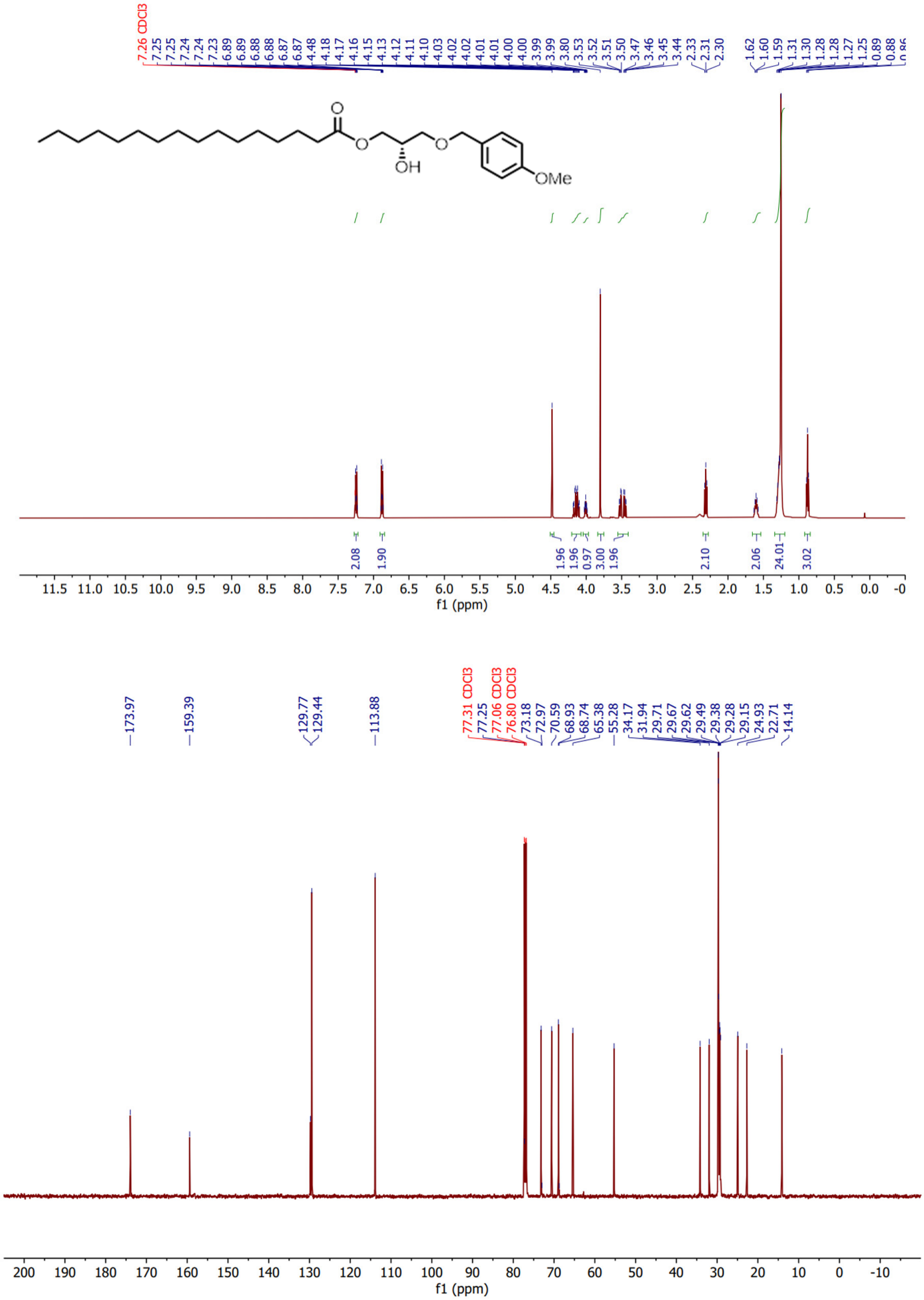


**Figure S19.** $^1$H and $^{13}$C NMR of **6a.**

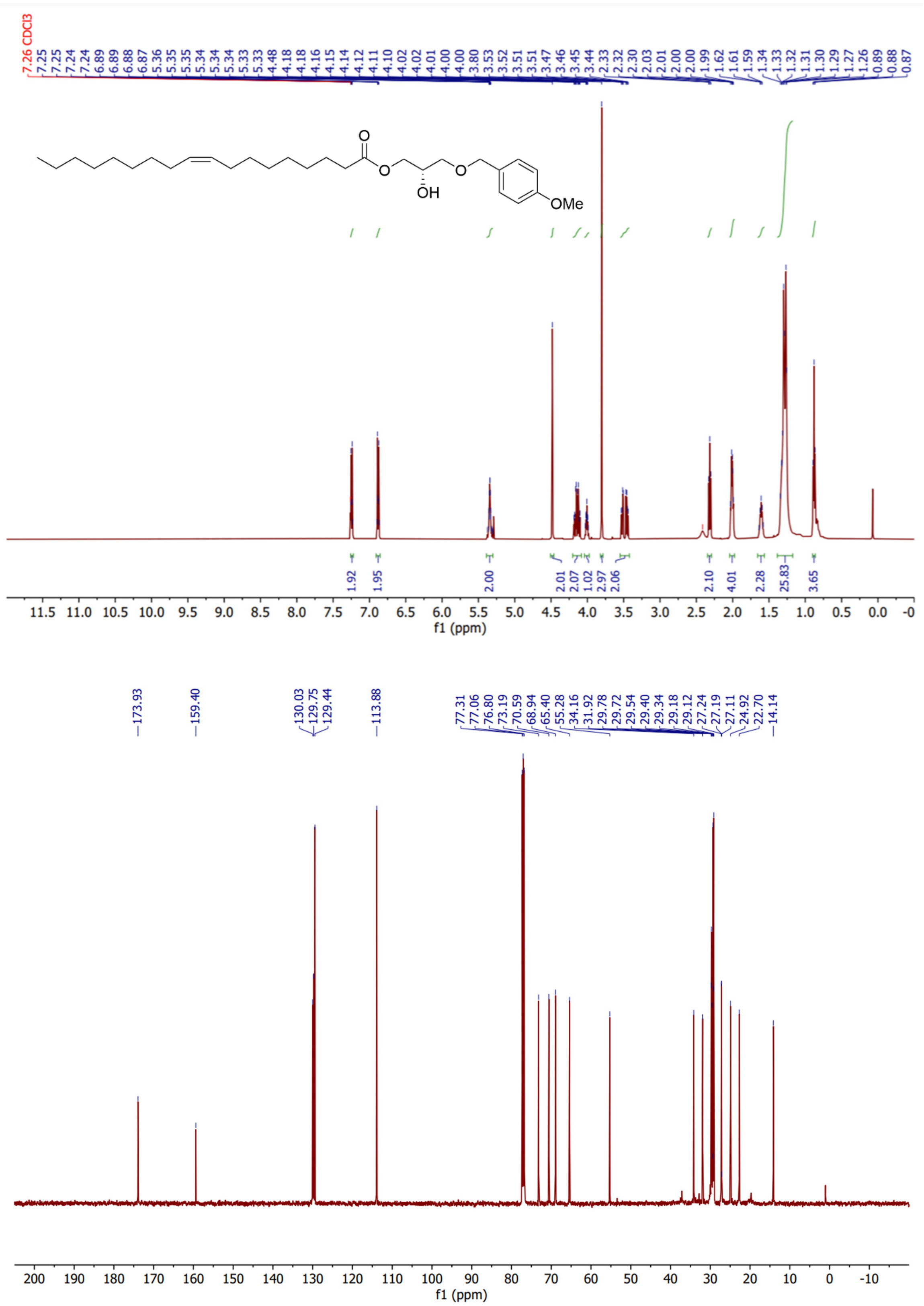


**Figure S20.** $^1$H and $^{13}$C NMR of **6b.**

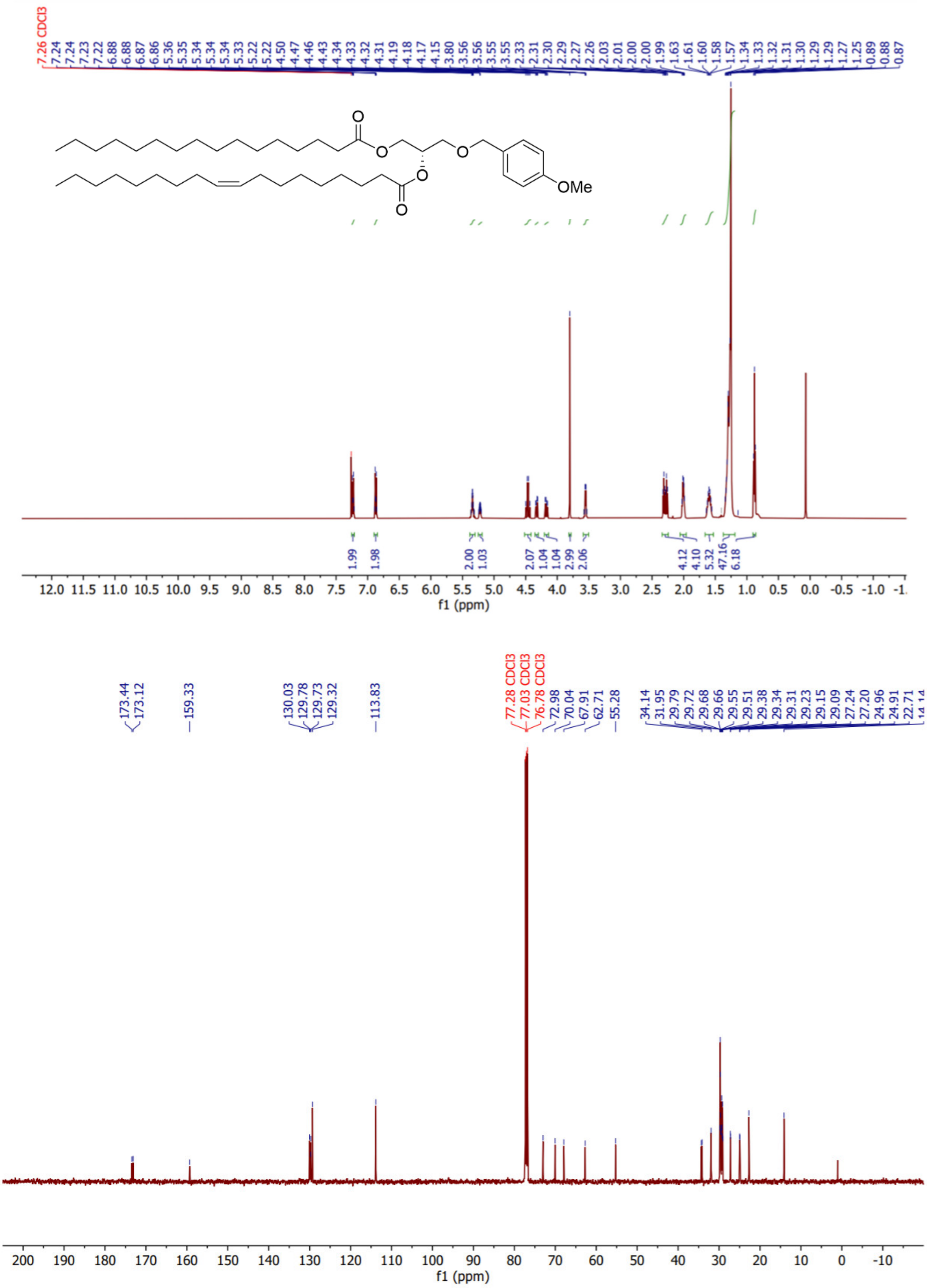


**Figure S21.** $^{1}H$ and $^{13}C$ NMR of **7a**.

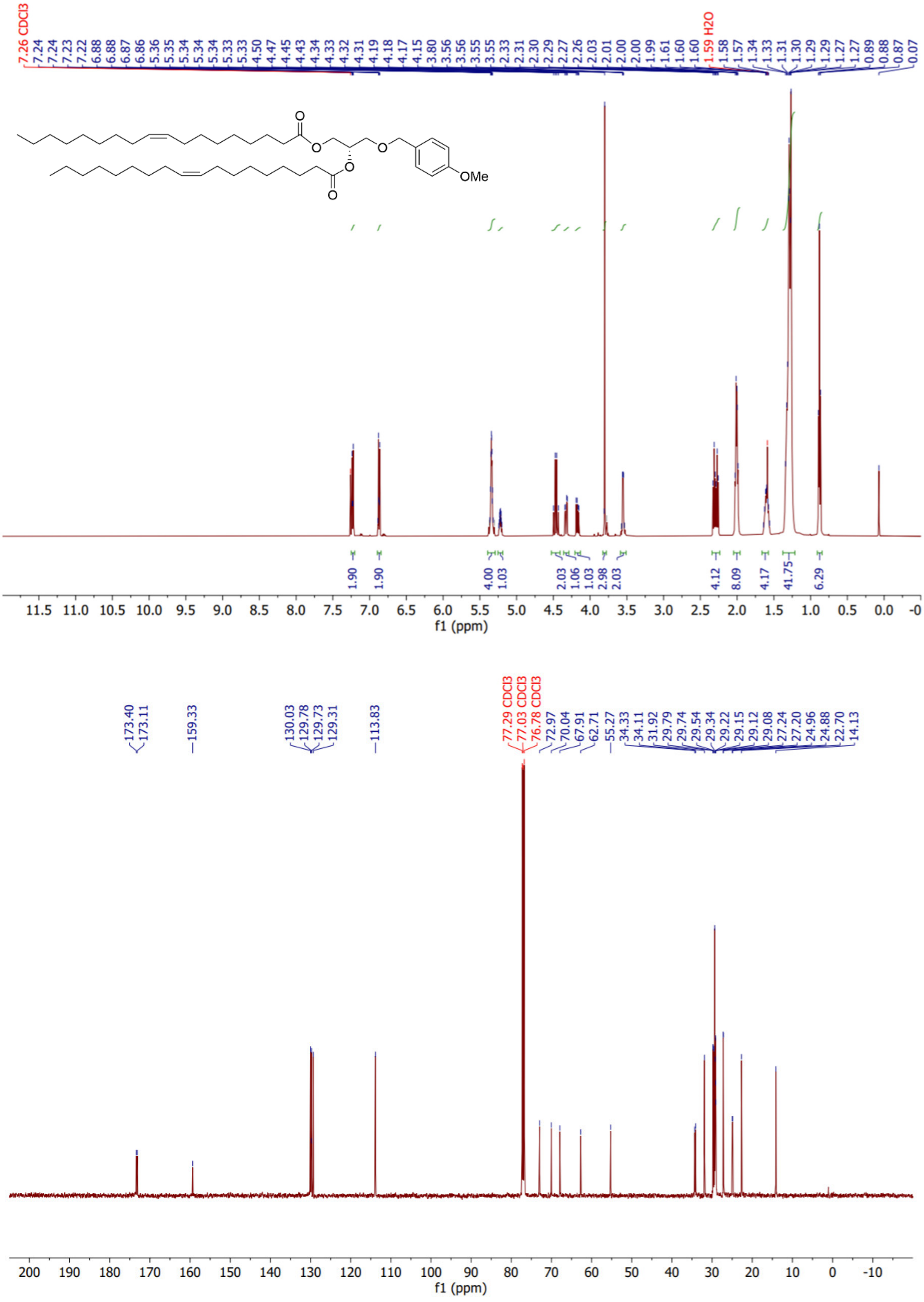


**Figure S22.** $^{1}H$ NMR and $^{13}C$ NMR of **7b.**

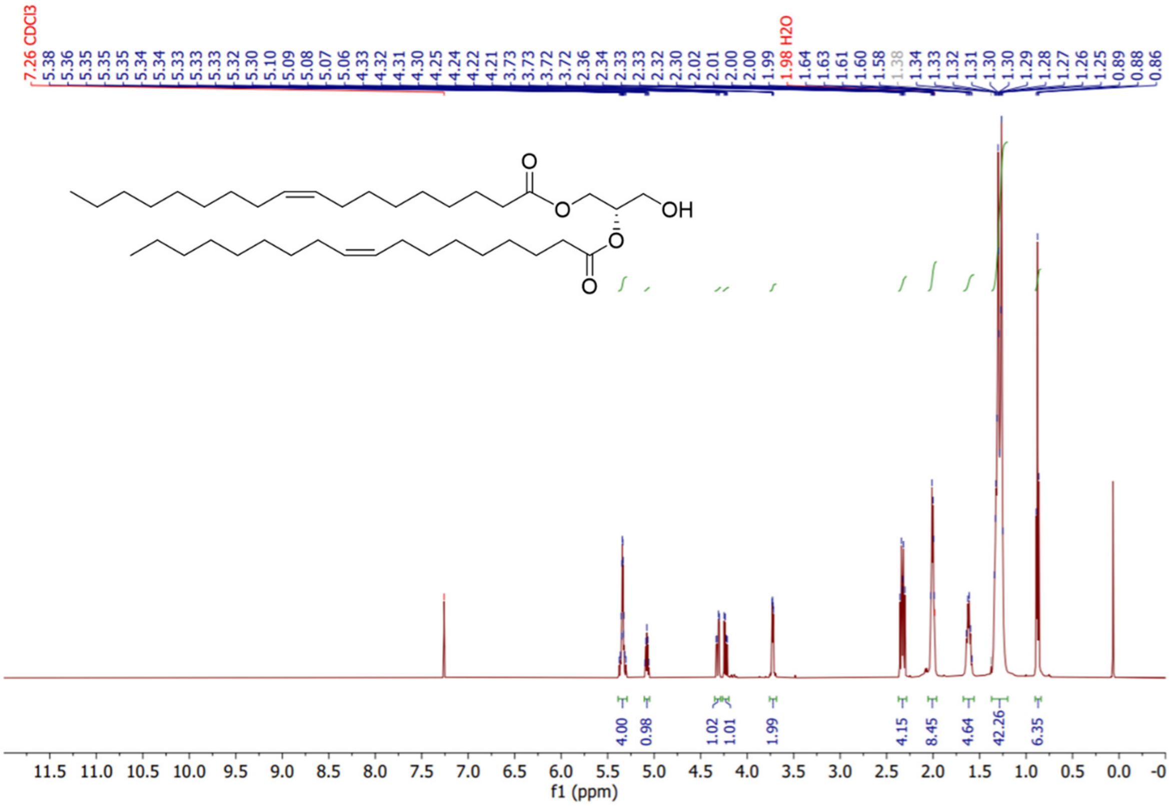

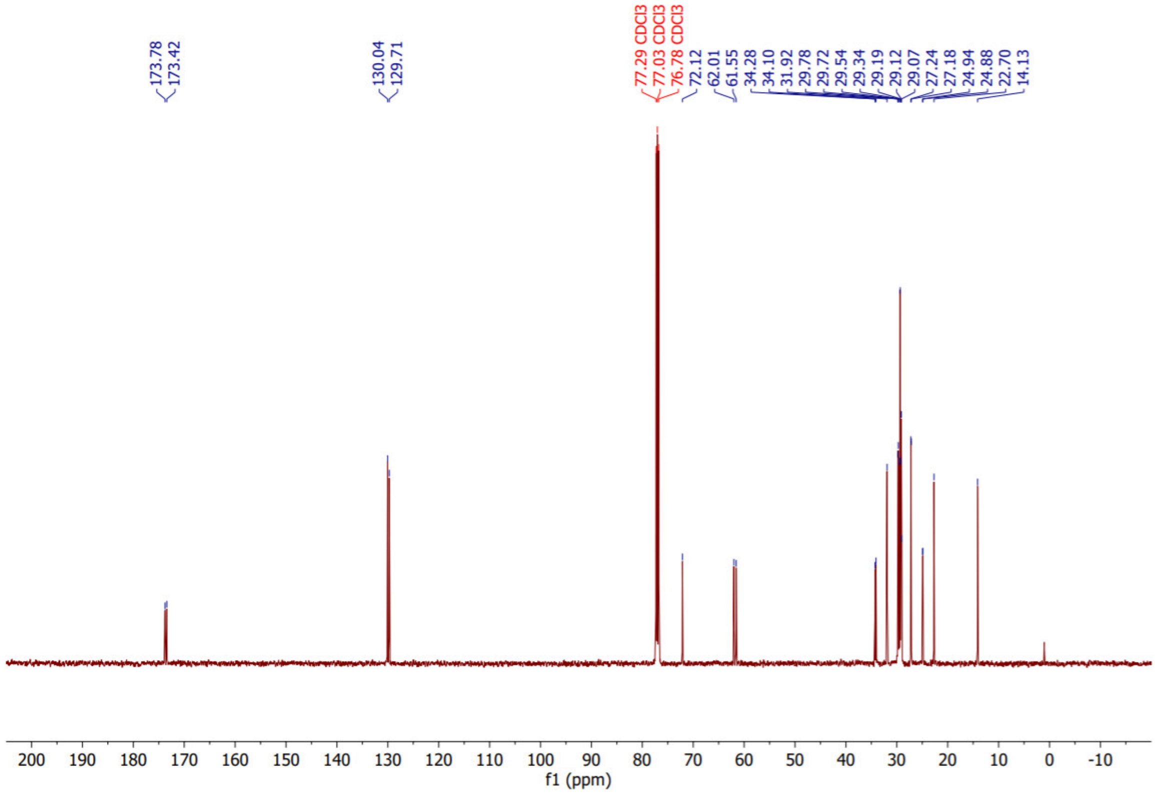

**Figure S23.** $^{1}H$ and $^{13}C$ NMR of **8b.**

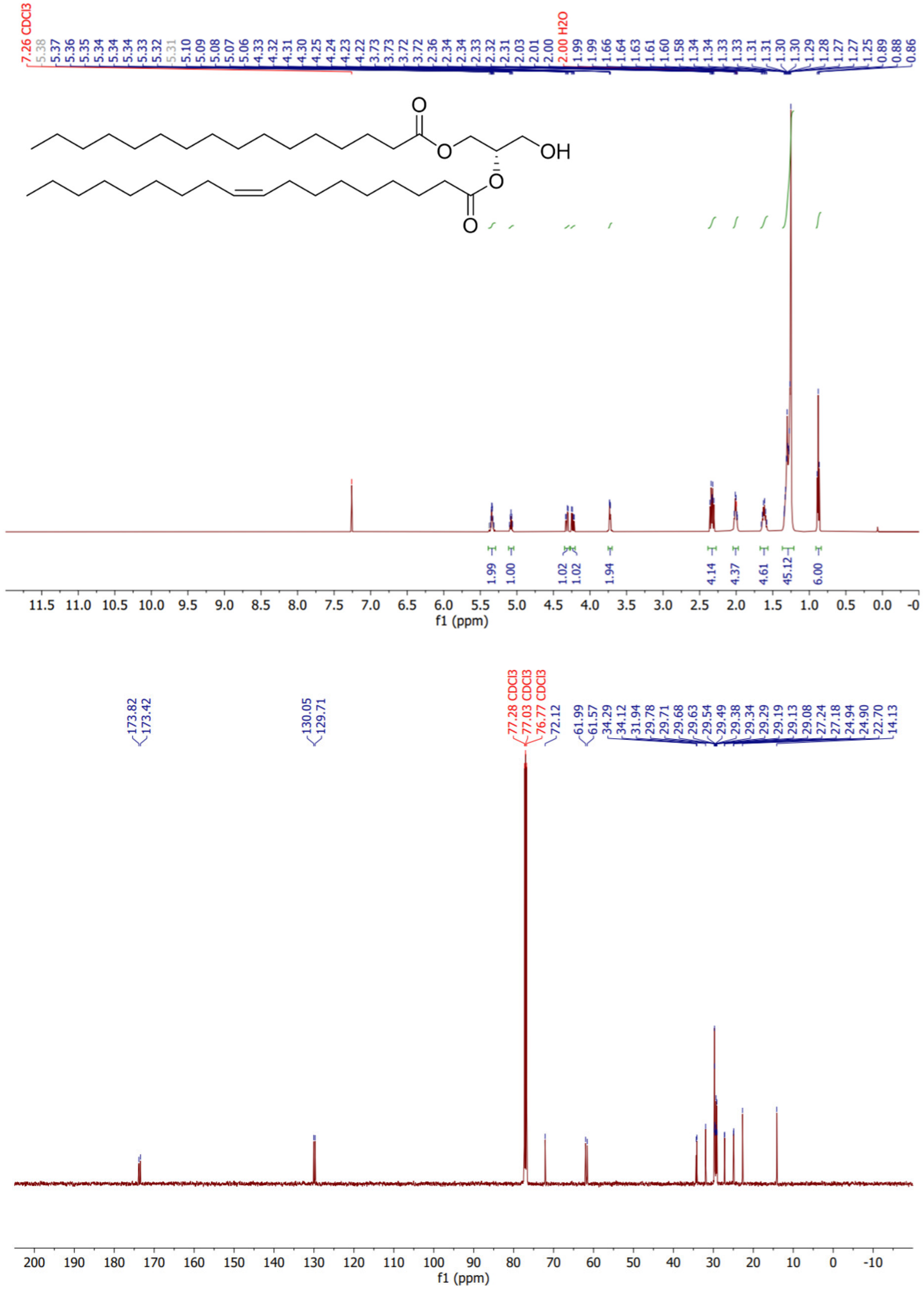


**Figure S24.** $^{1}H$ and $^{13}C$ NMR of **8a.**

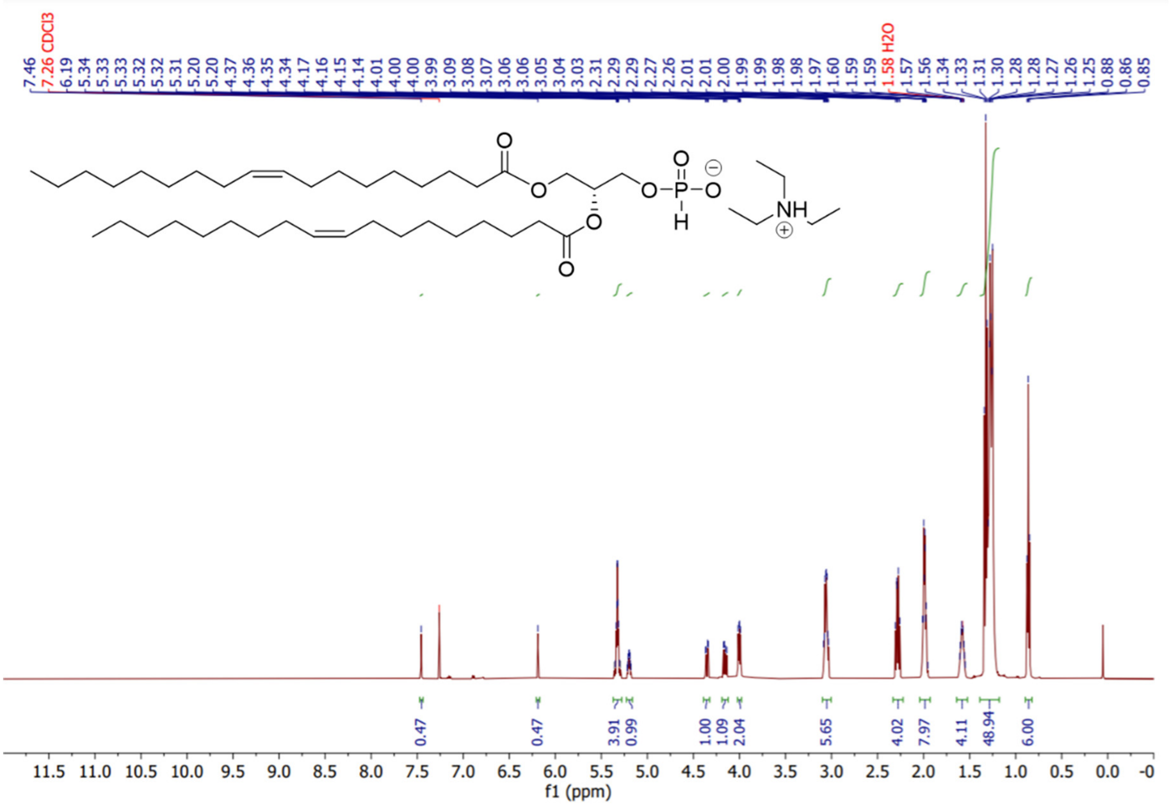
7.46
7.26 CDCl3
6.19
5.34
5.33
5.33
5.32
5.32
5.31
5.20
5.20
4.37
4.36
4.35
4.34
4.17
4.16
4.15
4.14
4.01
4.00
4.00
3.99
3.09
3.08
3.07
3.06
3.06
3.05
3.04
3.03
2.31
2.29
2.29
2.27
2.26
2.01
2.01
2.00
1.99
1.99
1.98
1.98
1.97
1.60
1.59
1.59
1.58 H2O
1.57
1.56
1.34
1.33
1.31
1.30
1.28
1.28
1.27
1.26
1.25
0.88
0.86
0.85
O
O
O
O–P–O
H
O
O
NH
0.47
0.47
3.91
0.99
1.00
1.09
2.04
5.65
4.02
7.97
4.11
48.94
6.00
11.5 11.0 10.5 10.0 9.5 9.0 8.5 8.0 7.5 7.0 6.5 6.0 5.5 5.0 4.5 4.0 3.5 3.0 2.5 2.0 1.5 1.0 0.5 0.0 -0
f1 (ppm)

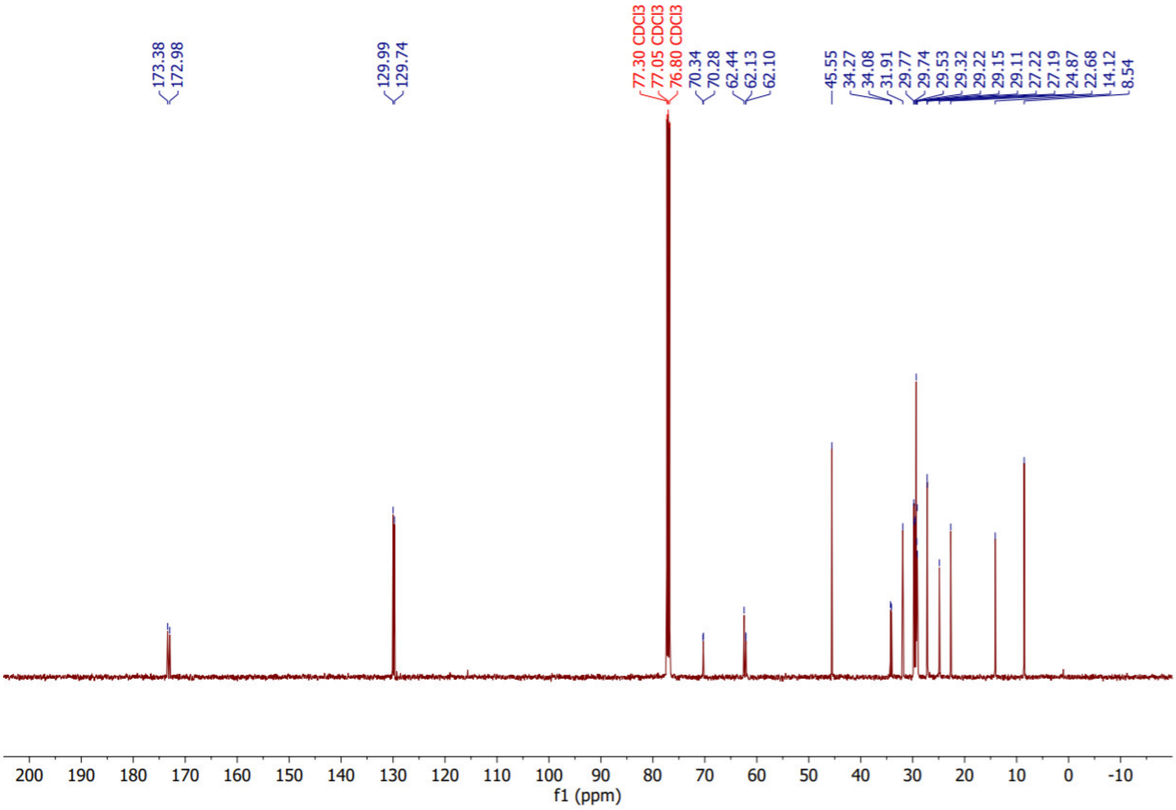
173.38
172.98
129.99
129.74
77.30 CDCl3
77.05 CDCl3
76.80 CDCl3
70.34
70.28
62.44
62.13
62.10
45.55
34.27
34.08
31.91
29.77
29.74
29.53
29.32
29.22
29.15
29.11
27.22
27.19
24.87
22.68
14.12
8.54
200 190 180 170 160 150 140 130 120 110 100 90 80 70 60 50 40 30 20 10 0 -10
f1 (ppm)

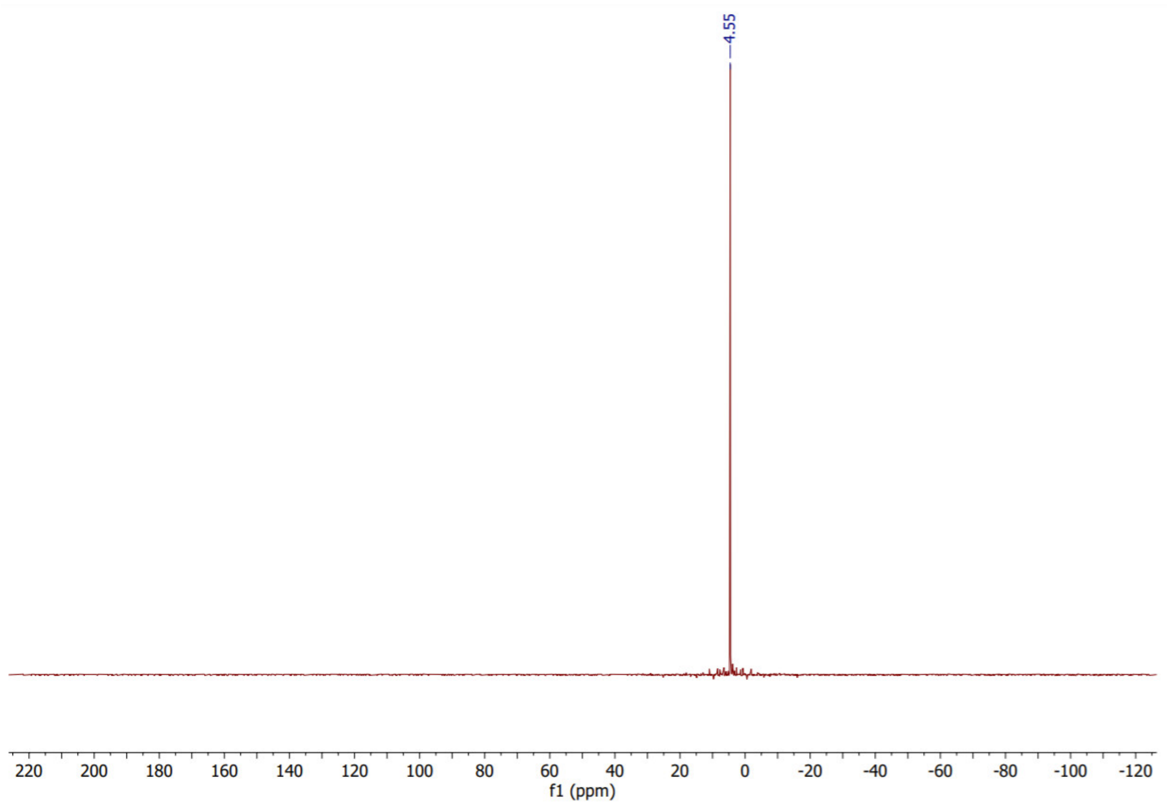


**Figure S25.** $^{1}H$, $^{13}C$, and $^{31}P$ NMR of **9b.**

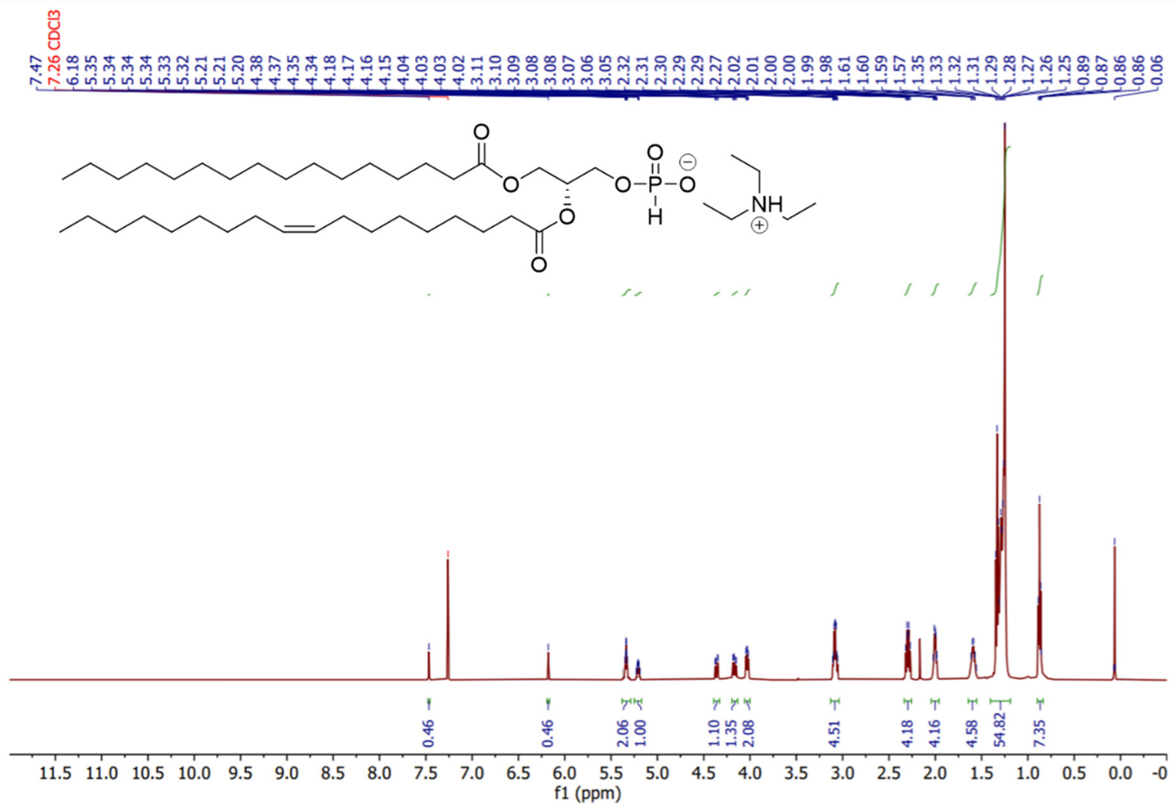

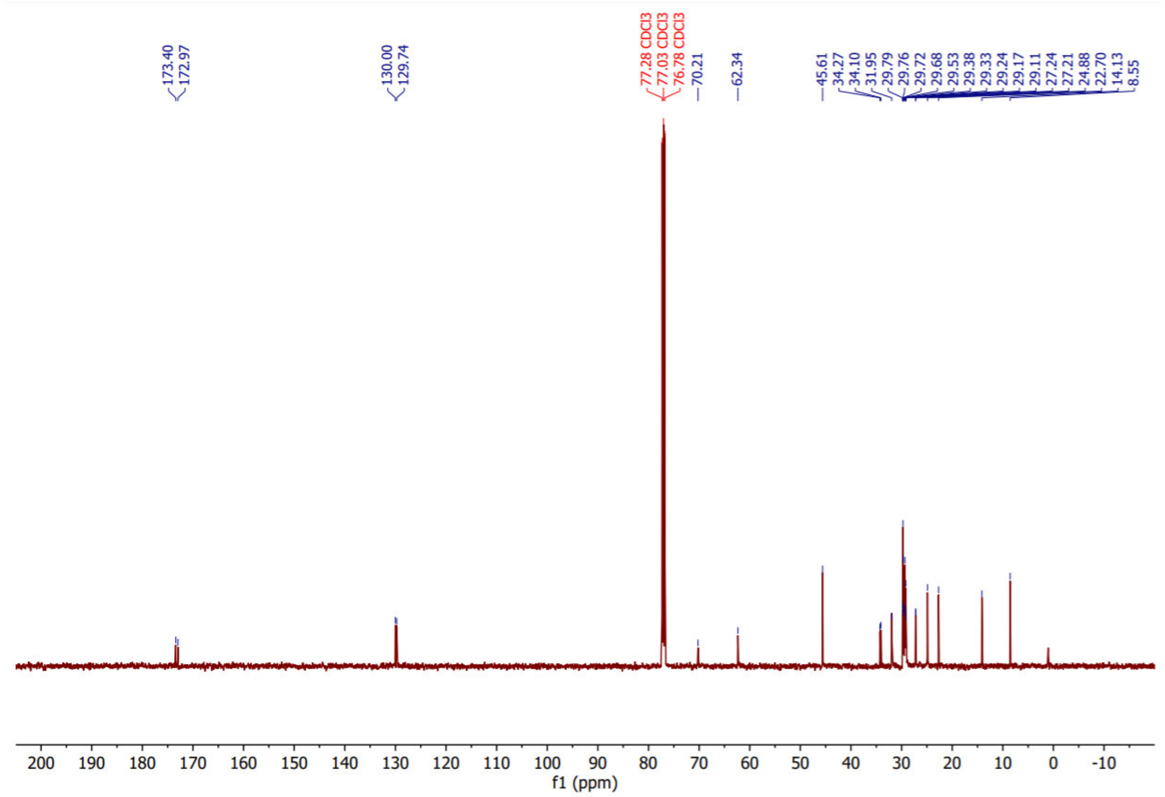


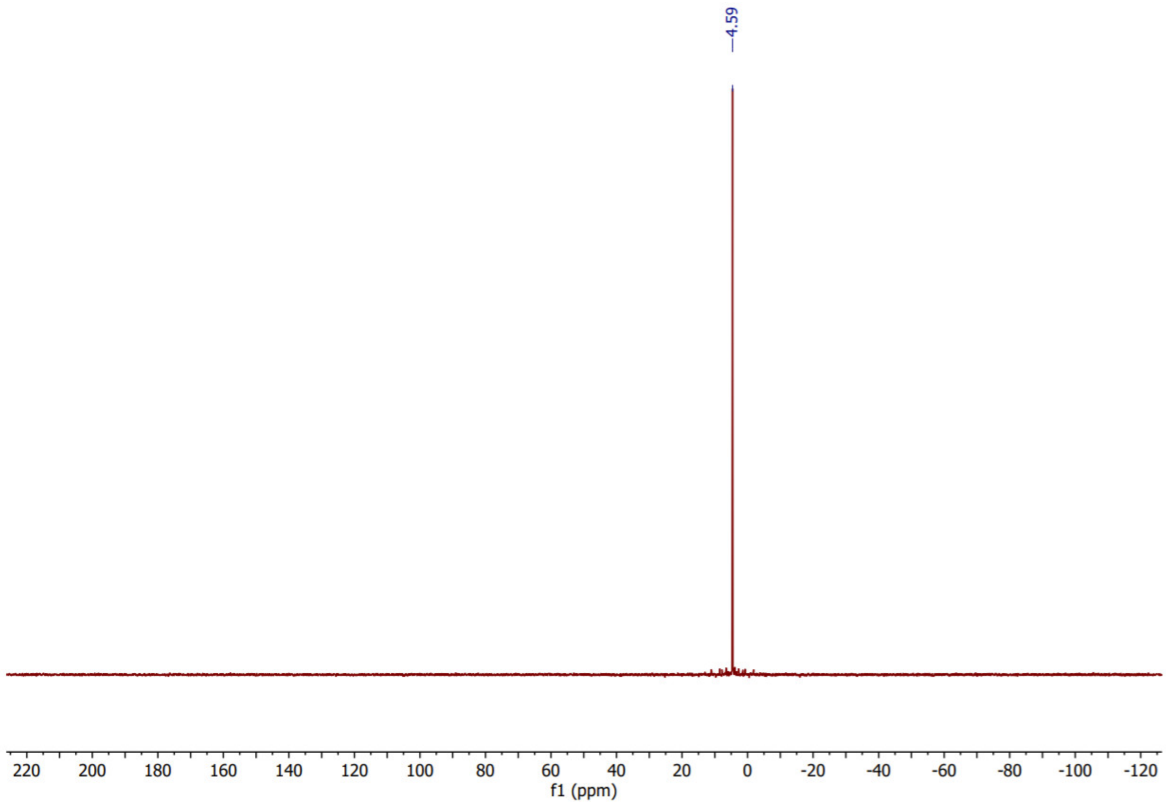


**Figure S26.** $^{1}$H, $^{13}$C, and $^{31}$P NMR of **9a.**

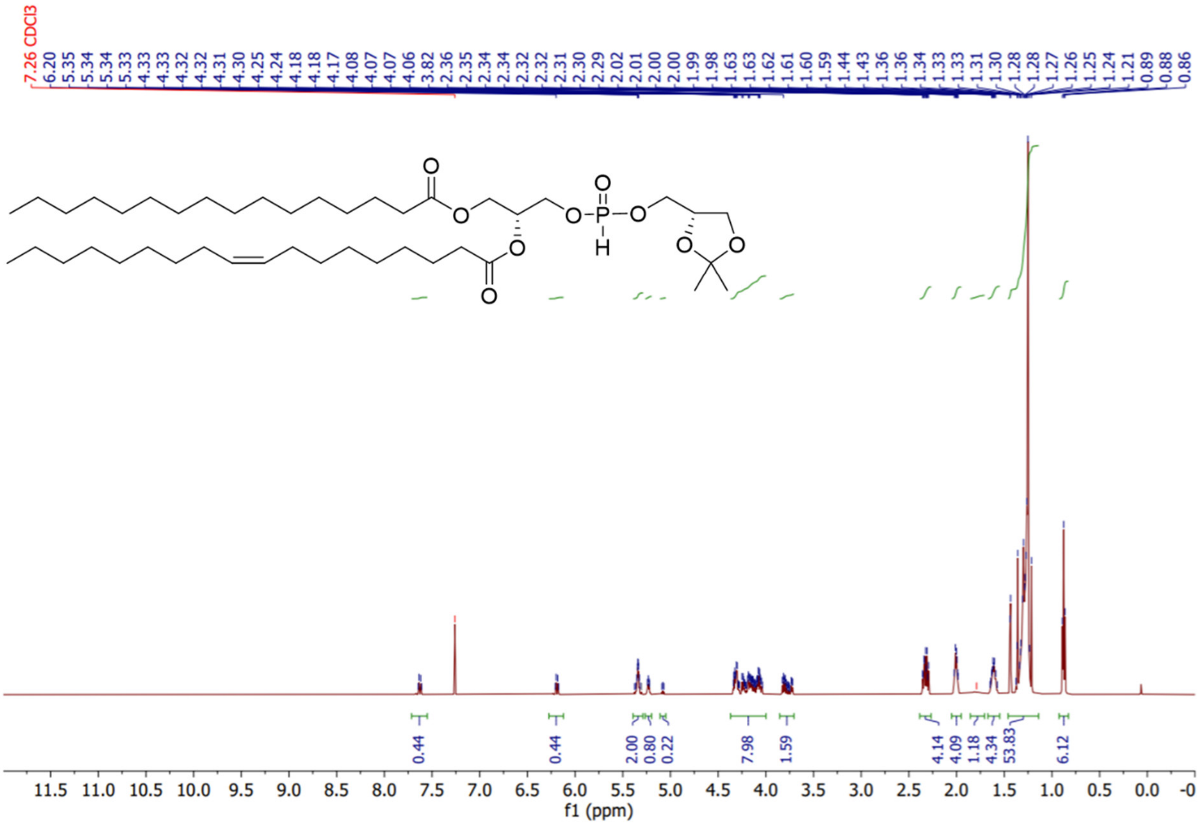
7.26 CDCl3
6.20 5.35 5.34 5.34 5.33 4.33 4.33 4.32 4.32 4.31 4.30 4.25 4.24 4.18 4.18 4.17 4.08 4.07 4.07 4.06 3.82 2.36 2.35 2.34 2.34 2.32 2.32 2.31 2.30 2.29 2.02 2.01 2.00 2.00 1.99 1.98 1.63 1.63 1.62 1.61 1.60 1.59 1.44 1.43 1.36 1.36 1.34 1.33 1.33 1.31 1.30 1.28 1.28 1.27 1.26 1.25 1.24 1.21 0.89 0.88 0.86
0.44 0.44 2.00 0.80 0.22 7.98 1.59 4.14 4.09 1.18 4.34 53.83 6.12
f1 (ppm)

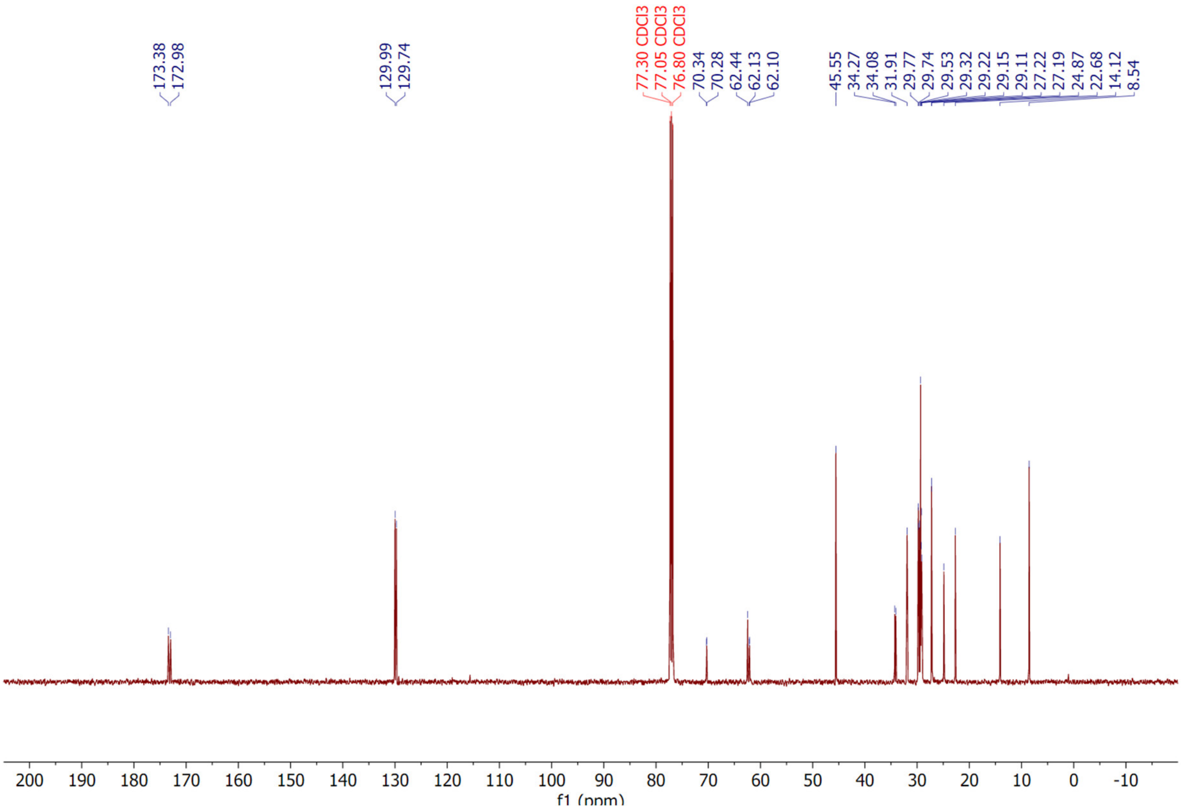
173.38 172.98 129.99 129.74
77.30 CDCl3
77.05 CDCl3
76.80 CDCl3
70.34 70.28 62.44 62.13 62.10 45.55 34.27 34.08 31.91 29.77 29.74 29.53 29.32 29.22 29.15 29.11 27.22 27.19 24.87 22.68 14.12 8.54
f1 (ppm)

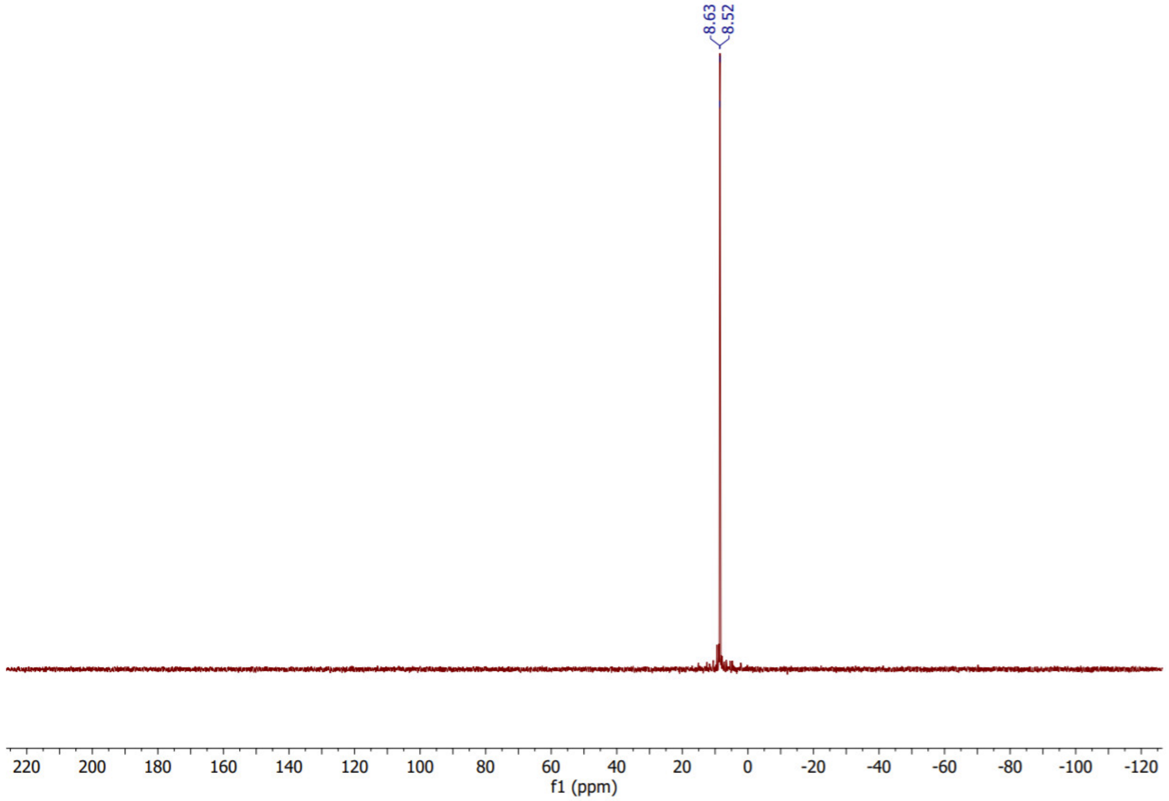


**Figure S27.** $^{1}$H, $^{13}$C, and $^{31}$P NMR of **11a.**

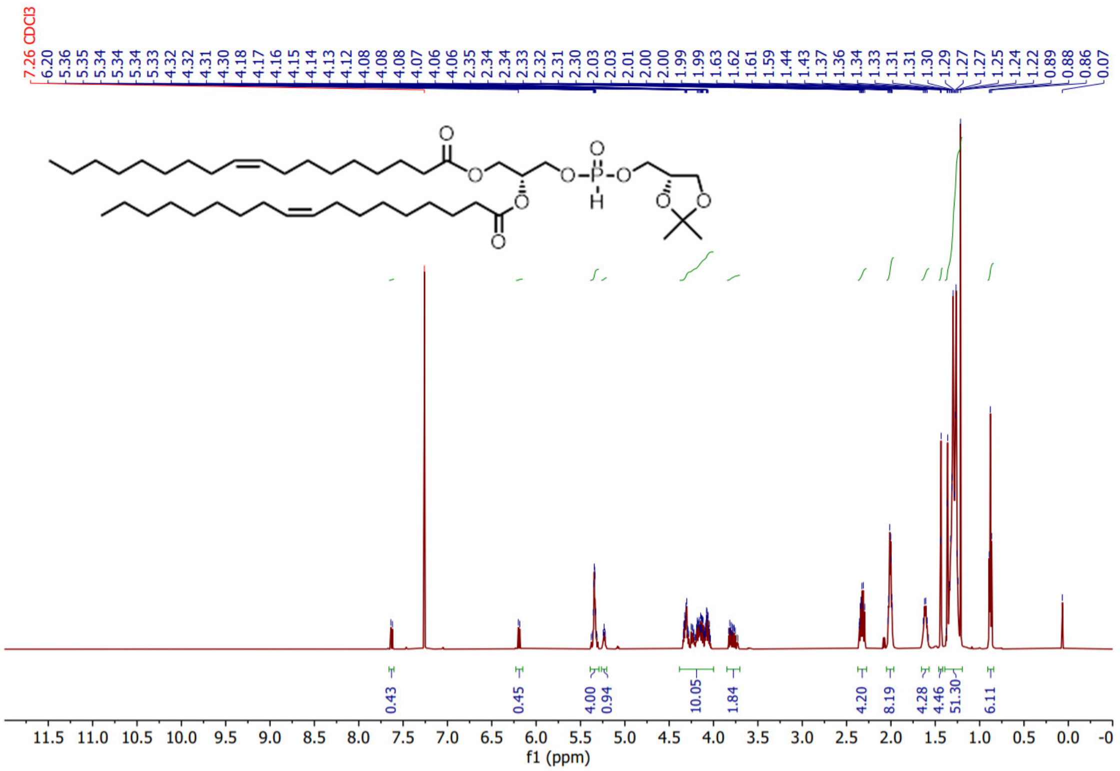

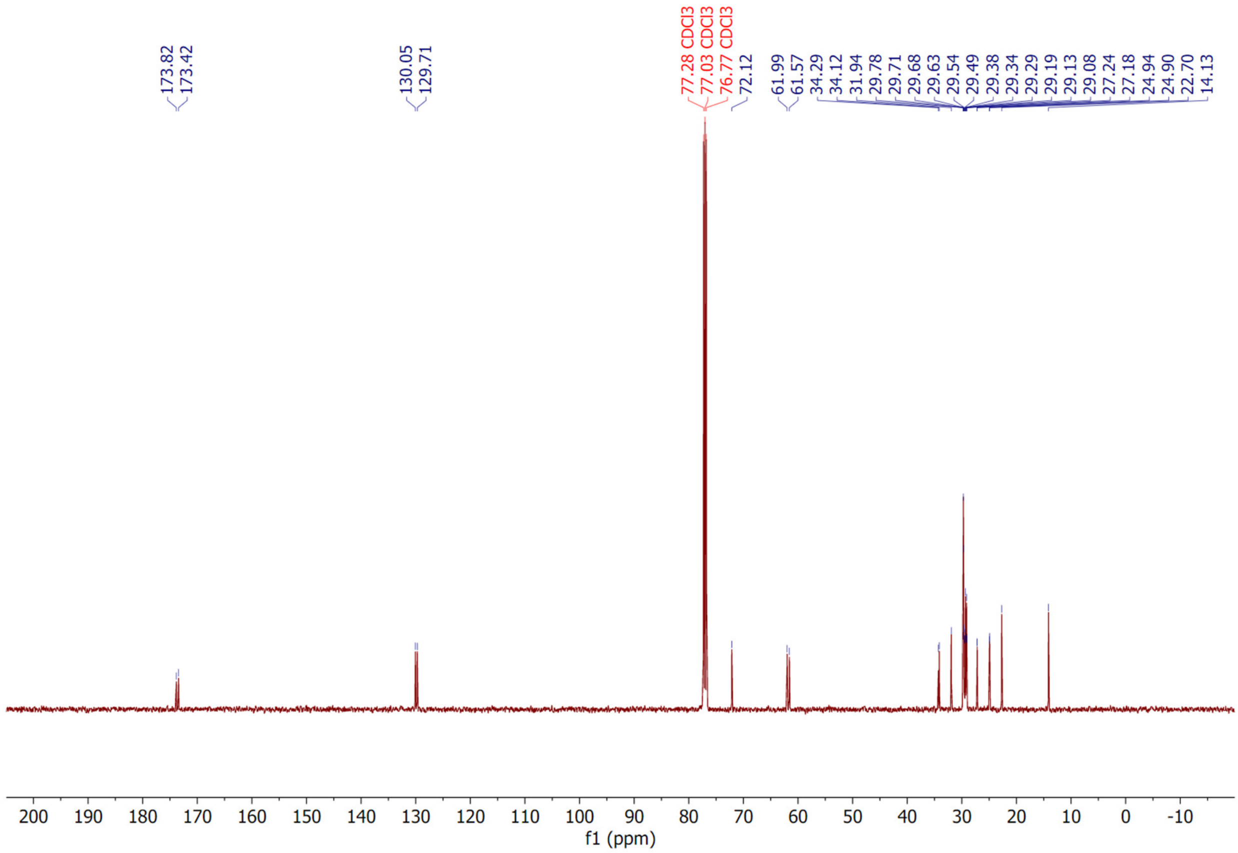


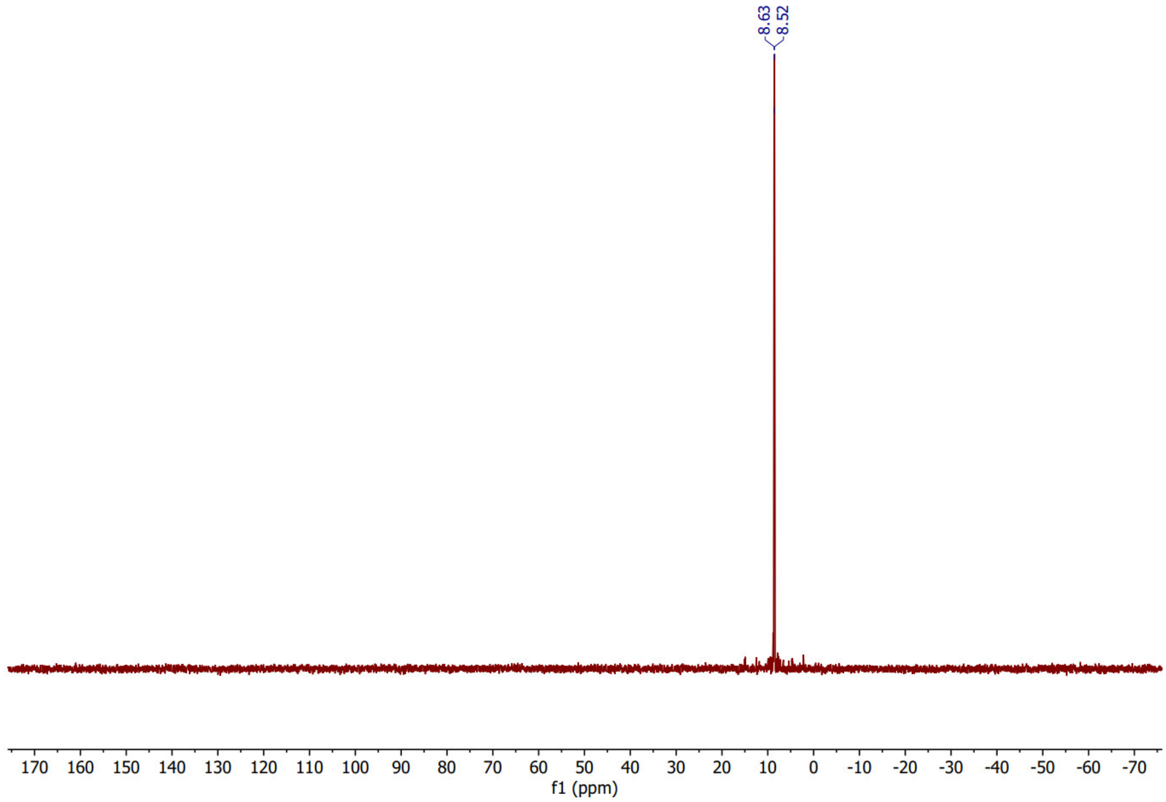


**Figure S28.** $^{1}$H, $^{13}$C, and $^{31}$P NMR of **11b.**

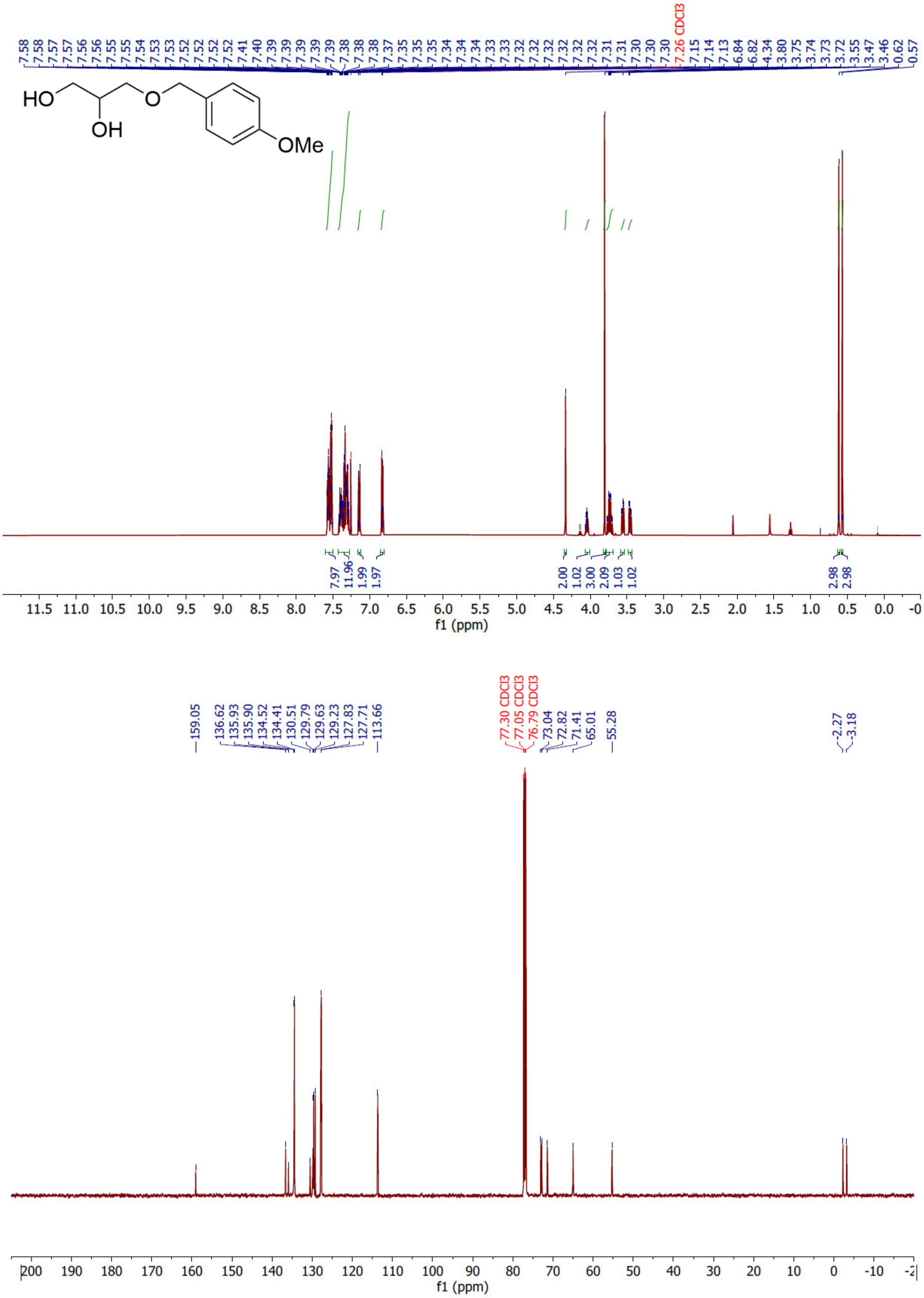


**Figure S29.** $^{1}H$ and $^{13}C$ NMR of **14.**

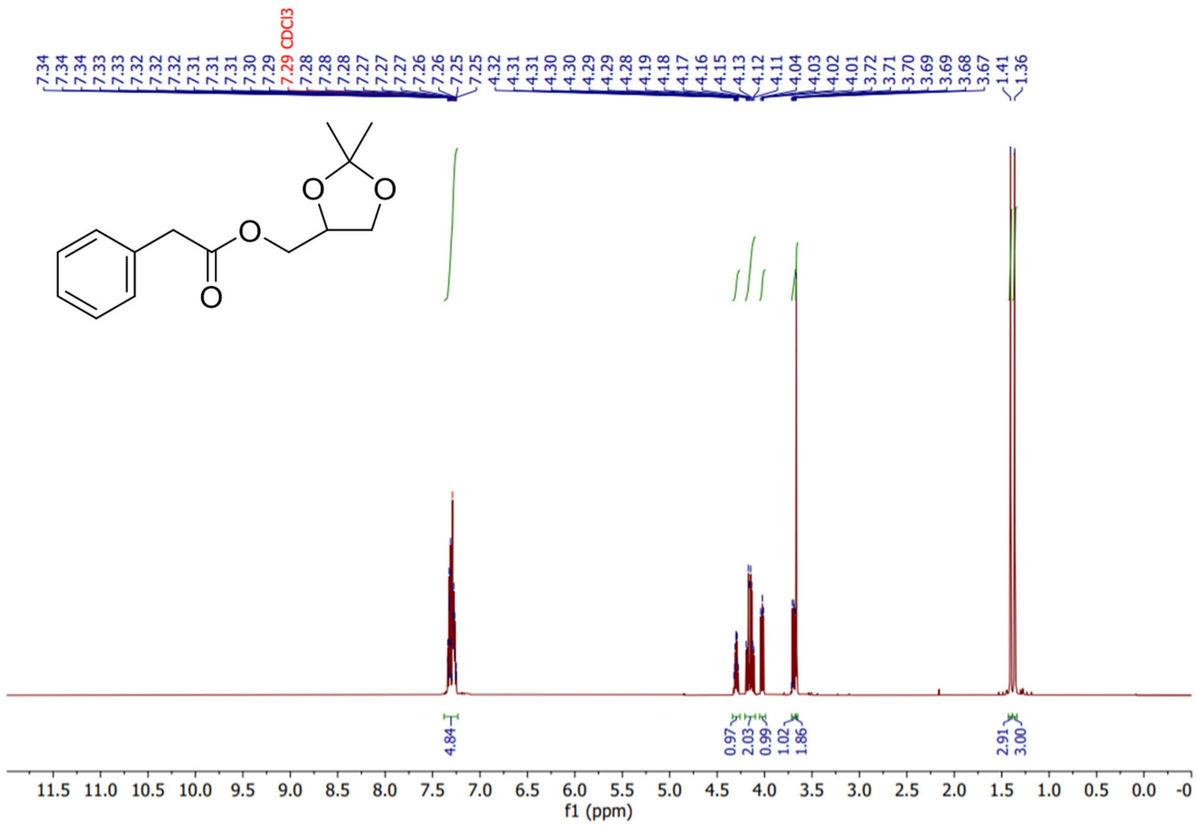

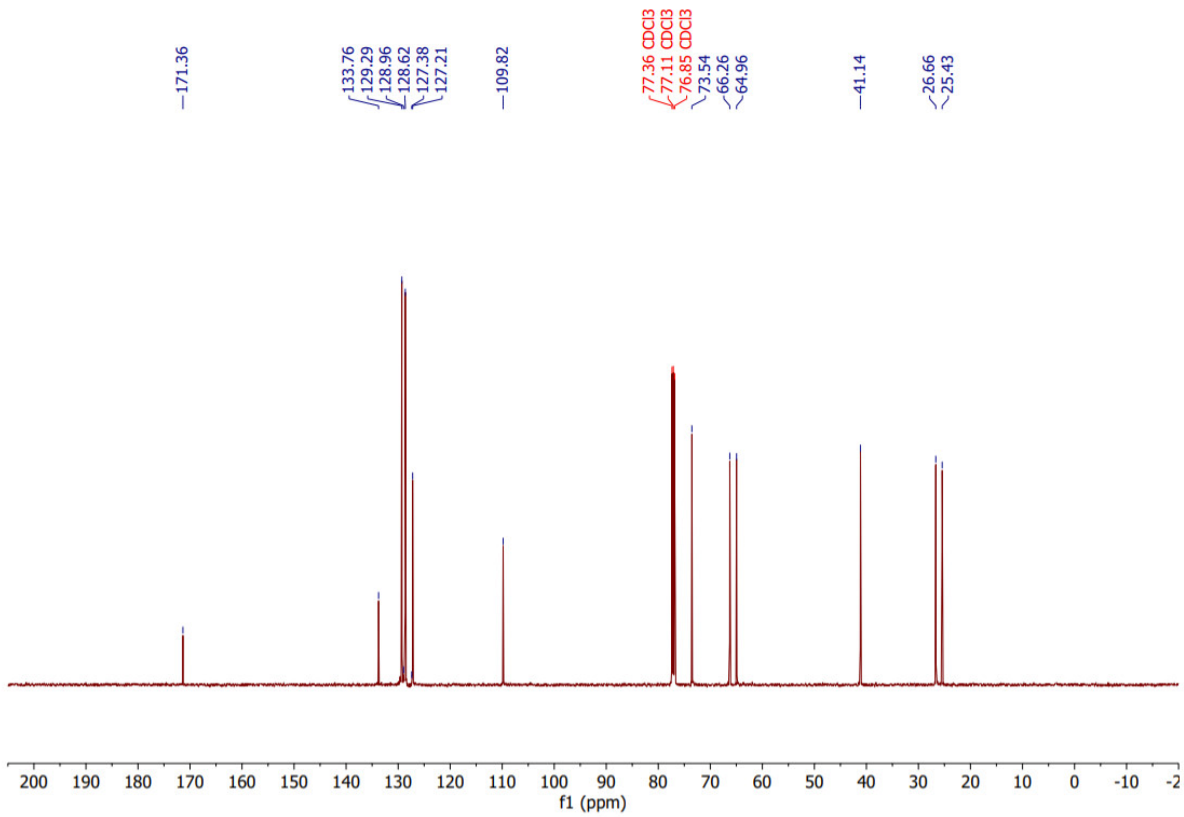

**Figure S30.** $^{1}H$ and $^{13}C$ NMR of **18.**

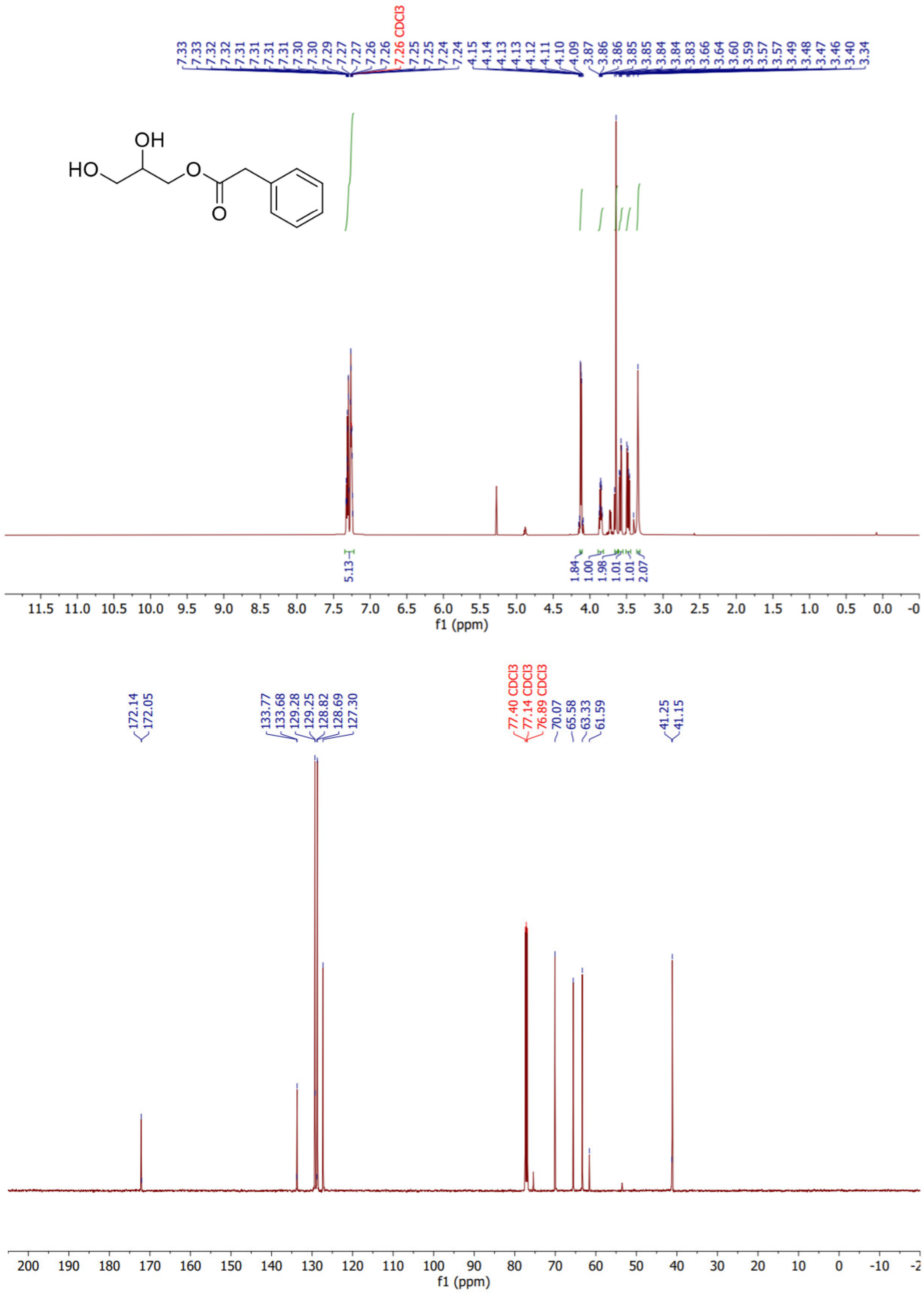


**Figure S31.** $^{1}H$ NMR and $^{13}C$ NMR of **19.**

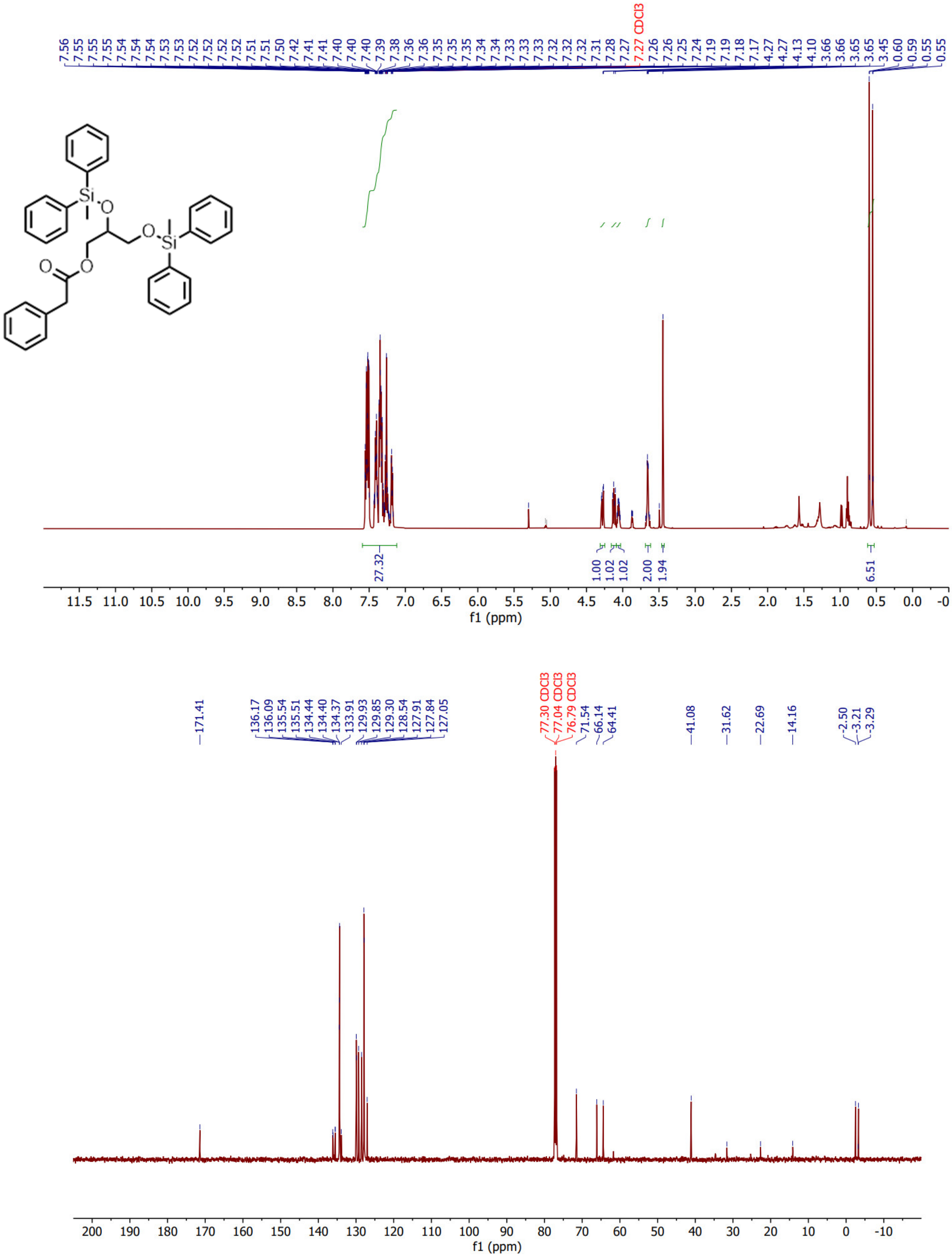


**Figure S32.** ¹H NMR and ¹³C NMR of **20.**

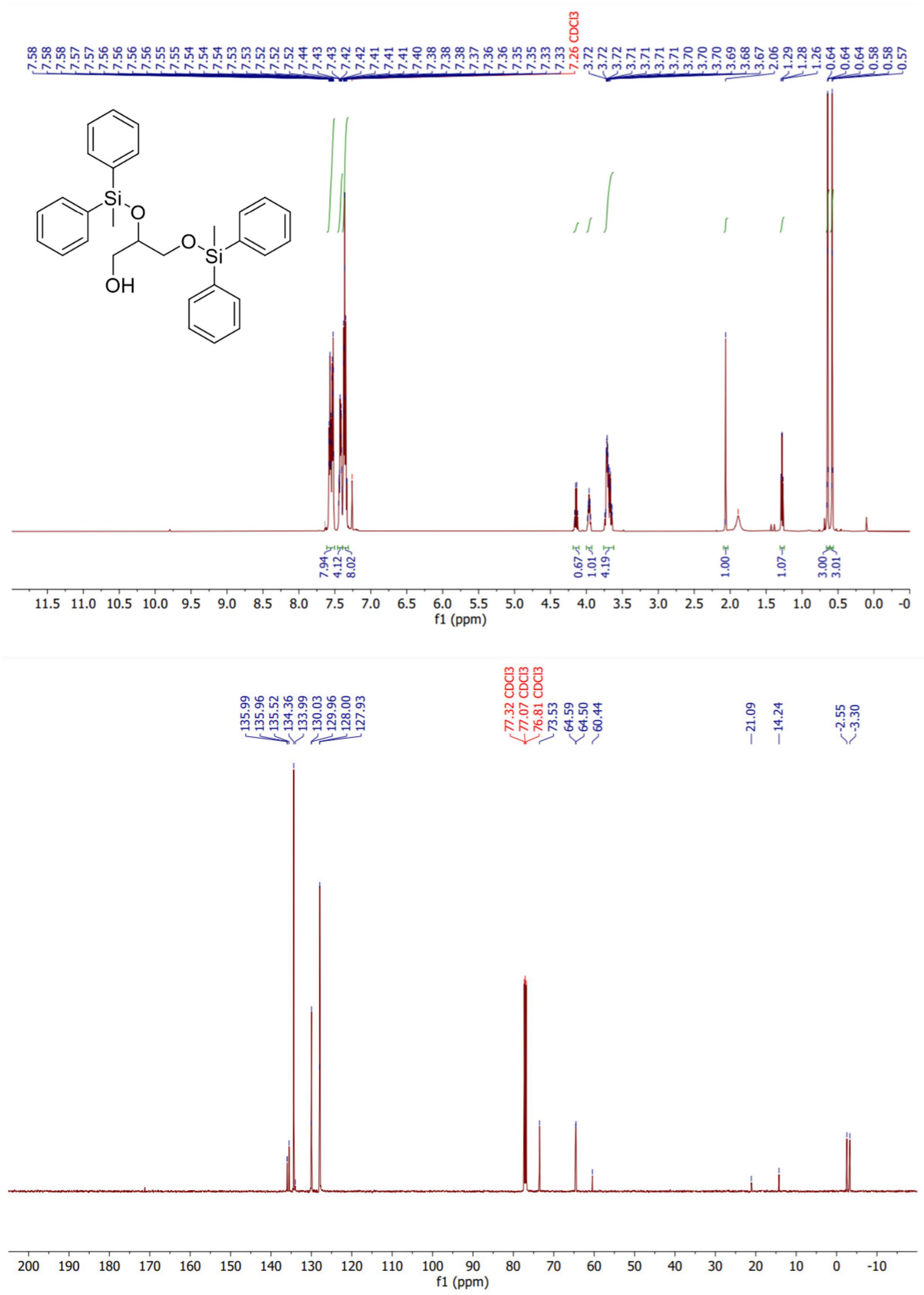


**Figure S33.** $^{1}H$ NMR and $^{13}C$ NMR of **21.**

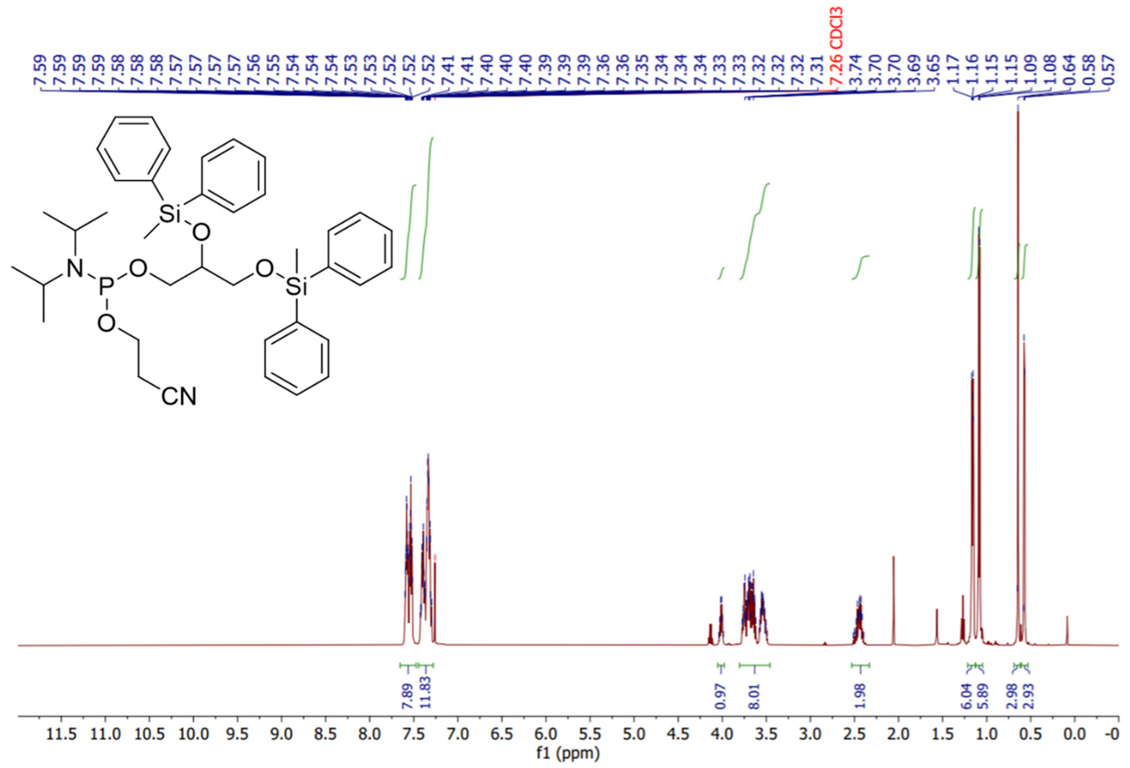
7.59 7.59 7.59 7.59 7.58 7.58 7.58 7.57 7.57 7.57 7.57 7.56 7.55 7.54 7.54 7.54 7.54 7.53 7.53 7.52 7.52 7.52 7.41 7.41 7.40 7.40 7.40 7.39 7.39 7.39 7.36 7.36 7.35 7.34 7.34 7.34 7.33 7.33 7.32 7.32 7.32 7.31
7.26 CDCl3
3.74 3.70 3.70 3.69 3.65 1.17 1.16 1.15 1.15 1.09 1.08 0.64 0.58 0.57
Si
O
N
P
O
O
O
Si
CN
7.89
11.83
0.97
8.01
1.98
6.04
5.89
2.98
2.93
11.5 11.0 10.5 10.0 9.5 9.0 8.5 8.0 7.5 7.0 6.5 6.0 5.5 5.0 4.5 4.0 3.5 3.0 2.5 2.0 1.5 1.0 0.5 0.0 -0
f1 (ppm)

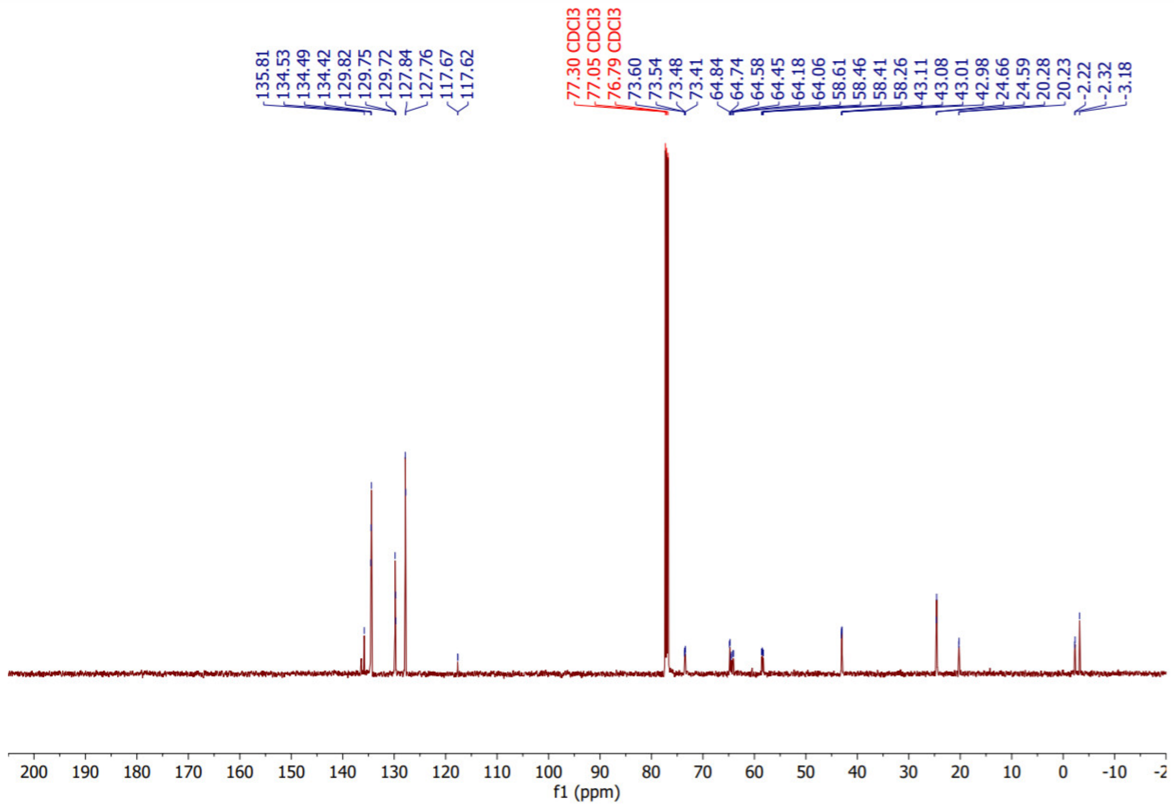
135.81 134.53 134.49 134.42 129.82 129.75 129.72 127.84 127.76 117.67 117.62
77.30 CDCl3
77.05 CDCl3
76.79 CDCl3
73.60 73.54 73.48 73.41 64.84 64.74 64.58 64.45 64.18 64.06 58.61 58.46 58.41 58.26 43.11 43.08 43.01 42.98 24.66 24.59 20.28 20.23 -2.22 -2.32 -3.18
200 190 180 170 160 150 140 130 120 110 100 90 80 70 60 50 40 30 20 10 0 -10 -2
f1 (ppm)

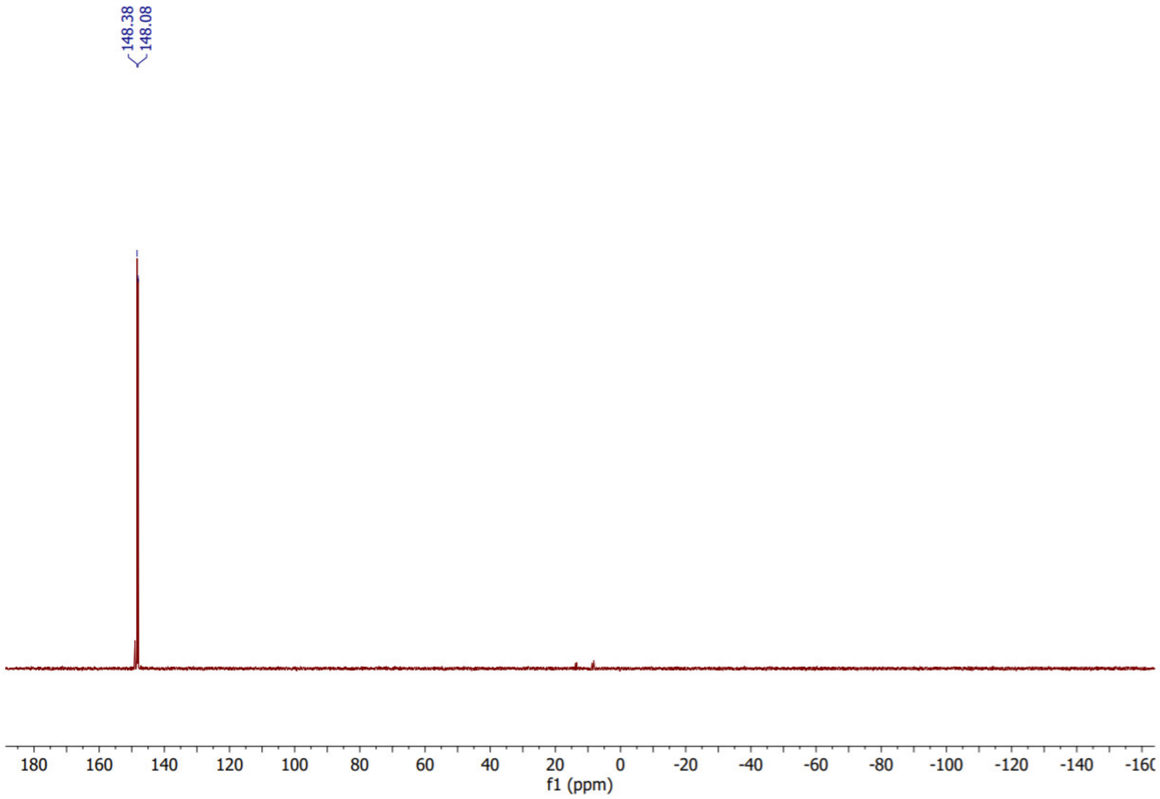


**Figure S34.** $^{1}$H NMR, $^{13}$C, and $^{31}$P NMR of **22.**
